# Supplementary material for: Neutralizing antibody immune correlates in COVAIL trial recipients of an mRNA second COVID-19 vaccine boost
Source: Nat Commun. 2025 Jan 17;16:759. doi: 10.1038/s41467-025-55931-w (PMC11748719; doi:10.1038/s41467-025-55931-w)
Supplement: Supplementary file 1 — Supplementary Information [file 41467_2025_55931_MOESM1_ESM.pdf]

COVAIL Manuscript Study Group

George Washington University, Washington D.C.

David J. Diemert, MD; Elissa Malkin, DO; Jeffrey M. Bethony, PhD; Aimee Desrosiers, PA-C; Marc Siegel, MD

University of Rochester VTEU, Rochester, NY

Angela R. Branche, MD; Ann R. Falsey, MD; Edward Walsh, MD; Patrick Kingsley, BS; Michael Peasley, BS

Emory University Hope Clinic, Decatur, GA

Nadine G. Rouphael, MD; Cecilia Losada, MD; Daniel S. Graciaa, MD; Hady Samaha, MD; Paulina A Rebolledo, MD; Zanthia Wiley, MD.

Brigham and Women's Hospital, Harvard Medical School, Boston, MA

Lindsey R. Baden, MD; Amy C. Sherman, MD; Stephen R. Walsh, MD; Alexandra Tong, BS; Rebecca Rooks, BS

Saint Louis University, St. Louis, MO

Sharon E. Frey, MD; Getahun Abate, MD, PhD; Zacharoula Oikonomopoulou, MD; Daniel F. Hoft, MD, PhD; Irene Graham, MD

Departments of Molecular Virology and Microbiology and Medicine, Baylor College of Medicine, Houston, TX

Jennifer A. Whitaker, MD; Hana M. El Sahly, MD; Wendy A. Keitel, MD; C. Mary Healy, MD; Robert L. Atmar, MD

Department of Medicine, Division of Infectious Diseases and Global Public Health, University of California San Diego, La Jolla, CA

Susan J. Little, MD; Thomas C.S. Martin, MD; Nicole Carter, MPH; Steven Hendrickx, RN

Center for Childhood Infections and Vaccines (CCIV) of Children's Healthcare of Atlanta and Emory University Department of Pediatrics, Atlanta, GA

Evan J. Anderson, MD; Christina A. Rostad, MD; Satoshi Kamidani, MD; Etza Peters, RN

Duke Human Vaccine Institute, Duke University School of Medicine, Durham, NC

Emmanuel B. Walter, MD, MPH; Michael J. Smith, MD, MSCE; M. Anthony Moody, MD; Kenneth E. Schmader, MD

University of Illinois at Chicago-Project WISH, Chicago, IL

Richard M. Novak, MD; Benjamin G. Ladner, MD; Andrea Wendrow, RPh; Jessica Herrick, MD

University of Texas Medical Branch, League City, TX

Richard Rupp, MD; Laura Porterfield, MD

Kaiser Permanente Washington Health Research Institute, Seattle, WA

Lisa A. Jackson, MD, MPH; Maya Dunstan, MS, RN; Rebecca Lau, PharmD; Barbara Carste, MPH

Department of Medicine, University of Washington, Seattle, WA

Tara M. Babu, MD, MSCI; Anna Wald, MD, MPH; Taylor Krause, BA; Kirsten Hauge, MPH

NYU VTEU Manhattan Research Clinic at NYU Grossman School of Medicine, New York, NY

Angelica C. Kottkamp, MD; Mark J. Mulligan, MD; Tamia Davis, NP; Celia Engelson, NP; Vijaya Soma, MD

Zuckerberg San Francisco General, University of California San Francisco, San Francisco, CA

Anne F. Luetkemeyer, MD; Chloe Harris, BA; Azquena Munoz Lopez, BS

Morehouse School of Medicine, Atlanta, GA

Lilly C. Immergluck, MD; Erica Johnson, PhD; Austin Chan, MD

Washington University School of Medicine, St. Louis, MO

Rachel M. Presti, MD, PhD; Jane A. O'Halloran, MD, PhD; Ryley M. Thompson

NYU VTEU Long Island Research Clinic at NYU Long Island School of Medicine, Mineola, NY

Martín Bäcker, MD; Andrew B. Fleming, MD; Asif Noor, MD

University of Iowa College of Medicine, Iowa City, IA

Patricia L. Winokur, MD; Jeffery Meier, MD; Jack Stapleton, MD

Howard University College of Medicine, Howard University Hospital, Washington D.C.

Siham M. Mahgoub, MD; Celia Maxwell, MD; Sarah Shami, PharmD

University of Alabama at Birmingham, Birmingham, AL

Paul A. Goepfert, MD

Tulane University School of Medicine, New Orleans, LA

Dahlene N. Fusco, MD; Arnaud C. Drouin, MD; Florice K. Numbi, MD

University of Maryland, Baltimore, MD

Kirsten E. Lyke, MD

IDCRC Principal Investigators

David S. Stephens, MD; Kathleen M. Neuzil, MD

IDCRC Leadership Operations Center

Monica M. Farley, MD; Jeanne Marrazzo, MD; Sidnee Paschal Young

IDCRC Clinical Operations Unit

Jeffery Lennox, MD; Robert L. Atmar, MD; Linda McNeil FHI360

IDCRC Statistical and Data Science Unit

Elizabeth Brown, PhD

IDCRC Laboratory Operations Unit – Fred Hutchinson Cancer Center, Seattle, WA

Christine M. Posavad, PhD; Megan A. Meagher, BS; Julie McElrath, MD; Mike Gale, PhD

FHI360, Durham, NC

Kuleni Abebe, MSc

The Emmes Company, LLC, Rockville, MD

Mat Makowski, PhD; Heather Hill, MS; Jim Albert, MS; Holly Baughman; Lisa McQuarrie, MS; Kalyani Telu, MS; Jinjian Mu, PhD; Lisa J. McQuarrie, MSc

Clinical Monitoring Research Program Directorate, Frederick National Laboratory for Cancer Research, Frederick, MD

Teri C. Lewis, BS; Lisa A. Giebeig, MS; Theresa M. Engel, MFS; Caleb J. Griffith, MPH; Wendi L. McDonald, BSN; Alissa E. Burkey, MS; Lisa B. Hoopengardner, MS; Jessica E. Linton, MS; Nikki L. Gettinger, MPH

Department of Surgery and Duke Human Vaccine Institute, Duke University School of Medicine, Durham, NC

David C. Montefiori, PhD; Amanda Eaton, MBA

Smith's Laboratory, Cambridge, UK

Derek J. Smith, PhD; Antonia Netzl; Samuel H. Wilks, PhD; Sina Türeli, PhD

Division of Microbiology and Infectious Diseases, National Institute of Allergy and Infectious Diseases, National Institutes of Health, Bethesda, MD.

Mamodikoe Makhene, MD; Mohamed Elsafy, MD; Rhonda Pikaart-Tautges, BS; Janice Arega, MS; Binh Hoang, RPh; Dan Curtin; Hyung Koo, BSN; Elisa Sindall, BSN; Sonja Crandon, BSN; Marciela M. DeGrace, PhD; Diane J. Post, PhD; Seema U. Nayak, MD; Paul C. Roberts, PhD; John H. Beigel, MD

COVAIL Manuscript Study Team Members

Emory University Hope Clinic, Decatur, GA

Nadine G. Rouphael, MD; Cecilia Losada, MD; Daniel S. Graciaa, MD; Hady Samaha, MD; Cassie Grimsley Ackerley, MD; Kristen E. Unterberger, PA; Amy Anderson, BSN; Mary Atha, ACNP; Kareem Bechnak, BSN; Sarah Bechnak, BSN; Mary Bower, BSN; Laura Clegg, RN; Matthew Collins, MD, PhD; Francine Dyer, RN; Srilatha Edupuganti, MD; Rebecca Fineman, BS; Tigisty Girmay, MSN; Rebecca Gonzalez, PharmD; Natalie Gray, BS; Evan Gutter, MPH; Lisa Harewood; Chris Huerta, MSc; Brandi Johnson, BS; Lauren Johnson, MPH; Colleen Kelley, MD; Alexandra Koumanelis, BA; Deborah Laryea, BSN; Hollie Macenczak, BSN; Nour Makkaoui, MD; Michele McCullough, MPH; Tuong-Vy Ngo, PharmD; Eileen Osinski, BS; Julia Paine, BS; Bernadine Panganiban, BS; Rose Pope, RN; Paulina Rebolledo, MD; Susan Rogers, RPh; Erin Scherer, PhD; Veronica Smith, NP-C; Andre Stringer, BS; Jessica Traenkner, PA; Dongli Wang, BS; Alahna Watson, BA; Stacey Wheeler, RN; Jean Winter; Jianguo Xu, PhD

Brigham and Women's Hospital, Harvard Medical School, Boston, MA

Lindsey R. Baden, MD; Amy C. Sherman, MD; Stephen R. Walsh, MD; Alexandra Tong, BS; Rebecca Rooks, BS; Jane A. Kleinjan, NP; Jon A. Gothing, NP; Andres A. Avila Paz, BA; Muneerah M. Aleissa, PharmD, MPH; Bethany Evans, BA; August Heithoff, BS; Natalie E. Izaguirre, MS; Hannah Jin, MPH; Urwah Kanwal, BS; Austin Kim, BS; Julia E. Klopfer, BS; Christina Montesano, BS; John Almeida, BA; Emily S. Koleske, BS; Hannah Levine, BS; Nicholas P. Morreale, BS; Omolola Ometoruwa, BS; Jun Bai Park Chang, BS; Anna F. Piermattei, BA; Djenane M. Pierre, BS; Megan Powell, BA; Kevin Zinchuk, PharmD; Stephanie Pickford, PharmD; Charles M. Kelly III, PharmD; Xiaofang Li, PhD; John Kupelian, BS ; Kimberly Dufresne, BS; Xiaoguang Fan, MD, PhD; Xi Zhang, PhD; Esther Arbona-Haddad, MD; Jose Humberto Licona, MD

Center for Childhood Infections and Vaccines (CCIV) of Children's Healthcare of Atlanta and Emory University Department of Pediatrics, Atlanta, GA

Evan J. Anderson, MD; Christina A. Rostad, MD; Satoshi Kamidani, MD; Etza Peters, RN; Larry Anderson, MD; Julia Bartol; Leisa Bower, RN; Natsuko Campbell, RN; Lisa Harewood; Hui-Mien Hsiao; Laila Hussaini, MPH; Inara Jooma; Gidget Kettle, RN; Marcia Lewis, RN; Wensheng Li; Cindy Lubbers, RN; Lisa Macoy, RN; Molly Morrison, Heather Nurse, RN; Anna Siaw-Anim; Kathleen Stephens, RN; Madeline Taylor; Ashley Tippett, MPH; Lauren Nolan, PA

Zuckerberg San Francisco General, University of California San Francisco, San Francisco, CA

Anne F. Luetkemeyer, MD; Chloe Harris, BA; Azquena Munoz Lopez, BS; Daniel Berrner; Dennis Dentoni-Lasofsky, MSN; John Dwyer, RN; Suzanne Hendler, BSN; Elvira Gomez, MPH; WeyLing Phuah, PharmD; Jaime Velasco, BA; Veronica Viar, MS

George Washington University, Washington D.C.

David J. Diemert, MD; Elissa Malkin, DO; Jeffrey M. Bethony, PhD; Aimee Desrosiers, PA-C; Marc Siegel, MD; Nikita Schroll-McLaughlin, MS; Jonathan Manning, BA; Jane Ryu, MS; Hanna-Grace Rabanes, MPH; Khadija Khan, MPH; Laura Vasquez, MPH; Caroline Thoreson, PA-C; Larissa Scholte, PhD; Rafaela Thur, DVM; Peyton St. John, BS; Dorinne Mettle-Amuah, PharmD

University of Iowa College of Medicine, Iowa City, IA

Patricia L. Winokur, MD; Jeffery Meier, MD; Jack Stapleton, MD; Laura Stulken, PA; Theresa Hegmann, PA; Deb Pfab, RN; Elizabeth Morgan, RN; Susan Herman, RN; Angel Peguero, CMA; Michelle Rodenburg; Alfred J. Carr; Delilah Johnson

Washington University School of Medicine, St. Louis, MO

Rachel M. Presti, MD, PhD; Jane A. O'Halloran, MD, PhD; Michael Klebert, RN, PhD; Ryley M. Thompson; Alem Haile; Kim Gray, NP; Chapelle Ayres; Delaney Carani, RN; Michael Royal; John Tran; Laura Blair; Anita Afghanzada; Natalie Schodl

NYU VTEU Manhattan Research Clinic at NYU Grossman School of Medicine, New York, NY

Angelica C. Kottkamp, MD; Tamia Davis, NP; Celia Engelson, NP; Vijaya Soma, MD; Abdulwahab Abdulai; Ashanay Allen; Natella Aronova, NP; Philip Aziz, PharmD; Emily Beato; Samuel Bliss, PharmD; Jacqueline Callahan, RN; Ellie Carmody, MD; Amanda Dontino, BS; Aimee Edwin, RN; Shelby Goins; Sarah Haiken; Ramin Herati, MD; Abdonnie Holder; Janice Hong; Trishala Karmacharya; Manpreet Kaur, PharmD; Hye-Youn Kim; Alexander McMeeking, MD; Mark Mulligan, MD; Wai Ng; Edward Nirenberg; Irma Noriega, NP; Samuel Nweke; Lalitha Parameswaran, MD; Levonne Phillip, MPH; Stephanie Rettig, MPH; Marie Samanovic-Golden, PhD; Madalyn Saporito; Pamela Suman; Meron Tasissa; Michael Tuen; Julia Wagner, MPH; James Wilson; Doris Wong, PharmD; Grace Yip, BS; Samantha Yip, RN; Heekoung Youn, RN; Lisa Zhao

University of Rochester VTEU, Rochester, NY

Angela R. Branche, MD; Ann R. Falsey, MD; Edward E. Walsh, MD; Patrick Kingsley, BS; Arthur Zemanek, BSN, MS; Katherine Elena, BSN; Spencer Obrecht, BSN; Ian Shannon, BSN; Amy Kaychalo, BS, MS; Erin Nowicki; Sharon Moorehead; Kari Steinmetz, BA; Doreen Francis, RN; Tanya Smith, BS; William Hamilton, BS; Jeanne Holden-Wiltse, MPH, MBA; Christopher Lane, MS; Michael Peasley, BS; Samuel Diehl, BS; Kyle Richards, PharmD; Stephen Bean, PharmD; Nicole Dornbush, PharmD; Carol Cole, PharmD

Saint Louis University, St. Louis, MO

Sharon E. Frey, MD; Getahun Abate, MD, PhD; Zacharoula Oikonomopoulou, MD; Daniel F. Hoft, PhD, MD; Irene Graham, MD; Azra Blazevic, DVM, MPH; Tamara Blevins, MS; Kathleen Chirco, BSN; Sabrina M. DiPiazza, BSN, MA; Stanley Doublin; Heather Hoertel Douds, MSNS, BSN; Carol G. Duane, PhD, RN; Eric Eggemeyer, BA; Linda M. Eggemeyer-Sharpe, BSN; Lauren Nicole Foreman, BSN; Sarah Louise George, MD; Geoffrey J. Gorse, MD; Michelle Harris, PharmD; Helay Hassas, PharmD; Rong Hou, MD; Ryan Clark Kerr, BSN; Kate Elizabeth Liefer, BSN; Melissa J. Loyet, RN; Lainey Mejia-Jauregui, BS; Keith Meyer, BS; Tracy Renee Montauk, BSN; Karla J. Mosby, RN; Amanda Nethington, BS; Huan Ning, MD; Nicole Purcell; Joan M. Siegner, BSN, MA; Janice M. Tennant, BSN, MPH; Mei Xia, PhD; Kiana Wilder, BA; Yinyi Yu, BS; Cassandra Nicole Zehenny, BSN

University of Texas Medical Branch, League City, TX

Richard Rupp, MD; Laura Porterfield, MD; Amber Stanford, PA-C; Robert Cox, RN; Kristin Pollock, RN; Diane Barrett, MS; Gerrianne Casey, RN; Amy McMahan, LVN; Cori Burkett, PA-C; Essie Cox

NYU VTEU Long Island Research Clinic at NYU Long Island School of Medicine, Mineola, NY

Martín Bäcker, MD; Sarah J. Pastolero, RN; Kimberly Byrnes, RN; Andrew B. Fleming, MD; Asif Noor, MD; Sigridh A. Muñoz-Gómez, MD; Steven E. Carsons, MD; Sajumon K. Joseph, FNP; Sophie Danziger; Monica Benitez; Maung Aung; Louis Ragolia, PhD; Alicia Vasile, RPh; April Correll, RPh; Christopher Hall; Thomas Palaia; Miloni H Thakker, MD; Lavern Harvey; Lisa Zhao; Diana Badillo, MD.

University of Illinois at Chicago-Project WISH, Chicago, IL

Richard M. Novak, MD; Benjamin G. Ladner, MD; Andrea Wendrow, RPh; Jesica Herrick, MD; Alfredo J. Mena Lora, MD; Scott A. Borgetti, MD; Diana L Bahena, APRN; Regina Harden, BA; Renyce

Powell; David C. M. Chan, PharmD; Rebeca F. Gasari, PharmD; Michael Pacini, PharmD; Margarita M. Villarreal, CPhT; Rodrigo Reyes, ADN; Samuel M. Rene, MPH; Shannon M Whitted, BSN; Habiba Sultana, MBBS; Nanu Kunwar, BS; Tasmin Sultana, MBBS; Md R. Amin, PhD; Mahmood Ghassemi, PhD, Liam Morrissy, BS; Nia O'Neal, BS; Chasity Serrano, BS; Charlie Peterson, BA

Duke Human Vaccine Institute, Duke University School of Medicine, Durham, NC

Emmanuel B. Walter MD, MPH; Michael J. Smith MD, MSCE; M. Anthony Moody, MD; Kenneth E. Schmader, MD; Susan Doyle; Lynn S Harrington BSN; Lori Hendrickson BSN; Amy O'Berry MSN; Sherry Huber BSN; Janet Wootton RN, RSCN; Kelly Clark BA; Lani Banez; Stephanie Smith BA; Byron Hauser BS; Ally Odom BA; Emily Randolph BA; Krystina Yoder BA; Kathlene Chmielewski; Luis Ballon BA; Aubree Latorre; Breana Montgomery; Antony Tritz MS; Thad Gurley, MS; Margaret Pendzich

Kaiser Permanente Washington Health Research Institute, Seattle, WA

Lisa A. Jackson, MD, MPH; Maya Dunstan, MS, RN; Rebecca Lau, PharmD; Barbara Carste, MPH; Wesley A. Andersen, RPh, MHA, MA; Lee Barr, RN; Cassandra Bryant, BS; Joe Choe, BS; Lynn Gross, PA-C; Erika Kiniry, MPH; Bonnie Y Lam, PharmD; De Vona Lang; Stella Lee, BA; Paula J Lins, PA-C, MPH; Amy Mohelnitzky, PA-C; Marilyn Nguyen, BS; Matthew Nguyen, MPH; Melissa Resendiz Rivas, BA; Melissa Boothe Scheer, PA-C; Janice Suyehira, MD; Stacie Wellwood, LPN; Maryann K Woodford, PA-C

Department of Medicine, Division of Infectious Diseases and Global Public Health, University of California San Diego, La Jolla, CA

Susan J. Little, MD; Thomas C.S. Martin, MD; Nicole Carter, MPH; Steven Hendrickx, RN; Ajay Bharti, MD; Alyssa Phillips; Aurora Verduzco Gonzalez, NP; Cheryl Dullano; Chris Houston; Dawn Rosenblum, RN; DeeDee Pacheco; DeLys Brooks; Fang Wan; Helene Le, CPhiT; JC Alcantar; Jill Blumenthal, MD; Joseph Lencioni, MABMH; Kory Hess; Letty Muttera, PharmD; Marlene Arredondo; Megan Smyth; Megan Taylor; Melinda Stafford, PharmD; Michelle Orsburn, MD; Michelle Truong; Niamh Higgins, PharmD, MSc, AAHIVP; Nimish Patel, PharmD, PhD, AAHIVP; Rebecca Gonzalez; Vivian Maldonado

Morehouse School of Medicine, Atlanta, GA

Lilly C. Immergluck, MD, MS; Erica Johnson, PhD; Austin Chan, MD; Fatima Ali, MPH; Sonja Jackson; Noor Mohamed, PharmD; LaKesha Tables, MD, MPH; Norberto Fas, MD; Kay Woodson, PharmD; Saadia Khizer, MD; Jacquelyn Ali, MSA; Abdullah Warsama; Eric Gaines; Sierra Jordan Thompson; Cristina Wilson; Trisha Parker, MPH; Xiting Lin; LaTeshia Thomas Seaton, APRN; Derrick Wilson

Howard University College of Medicine, Howard University Hospital, Washington D.C.

Siham M. Mahgoub, MD; Celia Maxwell, MD; Sarah Shami, PharmD; Edward Bauer, BS; Yuanxiu Chen, MD, PhD; Megan Ware-Pressley, MHA; Debra Ordor, RN; Linda Fletcher, RN; Emmanuel Baidoo, BS; David Jaspan, RPh, MBA; Adetokunbo Adedokun, PharmD, MPH, BCPS; Michelle Strobeck, BS; Michael A. Riga; Ashley Karen Bautista, BS

Departments of Molecular Virology and Microbiology and Medicine, Baylor College of Medicine, Houston, TX

Jennifer A. Whitaker, MD; Hana M. El Sahly, MD; Wendy A. Keitel, MD; C. Mary Healy, MD; Robert L. Atmar, MD; Pedro A. Piedra, MD; Jesus Banay; Kathy Bosworth; Janet Brown, RPh; Kayla Burrell; Jeremy Castro; Tykel Eddy; Marcena Eubanks; Cathy Faw, RPh; Rachel Froebe; Alix Halter, RN; Janey John, MSN, APRN, FNP-C; Chanei Henry, AAS; Vanessa Martinez; Carol Mundell, RN; Brandie

Phillips, RN; Alicia Prevost-Barthe, RN; Connie Rangel, RN; Yolanda Rayford, MS; Yvette Rugeley; Maria Shlyapobersky; Tina Sierra; Elizabeth Silguero; Lisreina Toro; Dawn Turner, RN; Chianti Wade-Bowers, RN; Jessica Woods, RN; Robert L. Atmar, MD

Departments of Medicine, Epidemiology, and Laboratory Medicine & Pathology, University of Washington, Vaccines and Infectious Diseases Division, Fred Hutchinson Cancer Center, Seattle, WA

Tara M. Babu, MD, MSCI; Anna Wald, MD, MPH; Taylor Krause, BA; Kirsten Hauge, MPH; Jina Taub, ARNP; Dana Varon, ARNP; Britt Murphy, ARNP; Morissa Pertik, PA-C; T. Nui Pholsena, ARNP; Alyssa Braun, BS; ; Jessica Heimonen, MPH; Amy Link, BS; Lindsey McClellan, BS; Jessica Moreno, BS; Chloe Wilkens, BS; Matt Seymour, MPH; Lawrence Hemingway, BS; Jean Mernaugh, BS; Chris McClurkan, BS; Kerry Laing, PhD; Meredith Potochnic, PharmD; Joong Kim, PharmD; Bao-Chao Vo, PhT

University of Alabama at Birmingham, Birmingham, AL

Paul A. Goepfert, MD; Jenna Weber, RN; Savannah Spaulding, RN; Heather Logan, CRNP; Faye Heard; Foreamben Patel; Michelle Chambers

Tulane University School of Medicine, New Orleans, LA

Dahlene N. Fusco, MD; Arnaud C. Drouin, MD; Florice K. Numbi, MD; Hamada F. Rady, PhD; Crystal A. Ward, MSN; Quinn M. Powers, MS; William E. Casey, BS; Brian P. Logarbo, MD; Shae P. Williams, BS; Emily Callegari, MSN

IDCRC Principal Investigators

David S. Stephens, MD; Kathleen M. Neuzil, MD

IDCRC Leadership Operations Center

Monica M. Farley, MD; Jeanne Marrazzo, MD; Sidnee Paschal Young

IDCRC Clinical Operations Unit

Jeffery Lennox, MD; Robert L. Atmar, MD; Linda McNeil FHI360

IDCRC Laboratory Operations Unit – Fred Hutchinson Cancer Center and University of Washington, Seattle, WA

Christine M. Posavad, PhD; Megan A. Meagher, BS; Michael Stirewalt, MBA; John Hural, PhD; Weston Lawler, BA; Lexi Tanser, MA; Julie McElrath, MD, PhD; Mike Gale, PhD

IDCRC Statistical and Data Science Unit

Elizabeth Brown, PhD

University of Maryland, Baltimore, MD

Kirsten E. Lyke, MD

FHI360, Durham, NC

Kuleni Abebe, MSc

The Emmes Company, LLC, Rockville, MD

Mat Makowski, PhD; Heather Hill, MS; Jim Albert, MS; Holly Baughman; Lisa McQuarrie, MS; Kalyani Telu, MS; Jinjian Mu, PhD; Lisa J. McQuarrie, MSc

Clinical Monitoring Research Program Directorate, Frederick National Laboratory for Cancer Research, Frederick, MD

Teri C. Lewis, BS; Lisa A. Giebeig, MS; Theresa M. Engel, MFS.; Caleb J. Griffith, MPH; Wendi L. McDonald, BSN; Alissa E. Burkey, MS; Lisa B. Hoopengardner, MS; Jessica E. Linton, MS; Nikki L. Gettinger, MPH; Aroussiak Bowen; Beth R. Baseler, MS; Vanessa S. Eccard-Koons, MS; Charles W. R. Hofsommer, JD; Thomas C. Sova, JD; Gary A. Krauss

Department of Surgery and Duke Human Vaccine Institute, Duke University School of Medicine, Durham, NC

David C Montefiori, PhD; Amanda Eaton, MBA; Francesca Suman, MS.

Smith's Laboratory, Cambridge, UK

Derek J Smith, PhD; Antonia Netzl; Samuel H Wilks, PhD; Sina Türeli, PhD; Ana Mosterín Höpping, PhD; Samuel Turner; Sarah James, MD; Poppy Roth

Division of Microbiology and Infectious Diseases, National Institute of Allergy and Infectious Diseases, National Institutes of Health, Bethesda, MD.

Marina Lee, PhD; Mamodikoe Makhene, MD; Mohamed Elsafy, MD; Rhonda Pikaart-Tautges, BS; Janice Arega, MS: Binh Hoang, RPh; Dan Curtin; Hyung Koo, BSN; Elisa Sindall, BSN; Aya Nakamura, RN, MS; Audria Crowder, BS; Guinevere Chun, RN, BSN, MSHS; Frank Kenny, PhD MPH; Seemi Patel, RHP, PharmD; Sonia Gales, MS; Ahsen Khan, JD; Walla Dempsey, PhD; Robert Jurao-RN, BSN; Sonja Crandon, BSN; Seema U. Nayak, MD; Marciela M DeGrace, PhD; Diane J Post, PhD; Paul C Roberts, PhD; John H Beigel, MD; SAVE Program

Methods

Baseline risk score

The baseline risk score is defined as the logit of the predicted COVID-19 outcome probability from a regression model estimated using the ensemble algorithm superlearner (i.e. stacking), where this logit predicted outcome is scaled to have empirical mean zero and empirical standard deviation one. The settings of superlearner (i.e., loss function, cross-validation technique, library of learners) that are used for implementation of superlearner for building a baseline risk score are described in Section 8.4 of the Statistical Analysis Plan. To quantify the predictive power of the risk score, the cross-validated prediction performance was characterized by point estimate and 95% confidence interval estimates of the cross-validated area under the ROC curve (CV-AUC).

The following baseline demographic input variables were included for building the risk scores: age in years, indicator of age ≥ 65, sex assigned at birth (Male/Female), ethnicity, race, the number of days from last prior vaccination until enrollment, the indicator that the number of days from last prior vaccination until enrollment is greater than the median value, and the type of last vaccine received prior to the booster. The baseline risk score was intended to help capture exposure information or elevated risk information while allowing variables with a distinctive immunological meaning to be included separately, facilitating the ability to interpret associations/effects attached to these immunological variables. SARS-CoV-2 naïve/non-naïve status, vaccination type information, and D1 titers were not included as input variables into the baseline risk score, as they were treated in a special way for various correlates analyses.

Force of infection score

The Coronavirus Resource Center's database provides COVID-19 case numbers and incidence rates in the U.S. from 23 January 2020 through 09 March 2023. Each COVAIL study participant's FOI score is computed as the average of daily COVID-19 incidence rates (number of cases per 100,000 persons) over all days spanning from 7 days after their D15 visit through to 188 days post enrollment. The JHU database contains data at the geographic level of U.S. state and some territories, so the participants' geographic locations (at the level of state or the District of Columbia) were factored into the calculation.

FOI scores were not calculated for participants who missed their D15 visit, which does not impact correlates analyses given that D15 visit attendance is required for inclusion. Additionally, 200 participants had follow-up periods that extended beyond the JHU database's final day of 09 March 2023. (The latest 188 days post booster date for a COVAIL participant was 04 May 2023.) The case numbers for these missing dates were extrapolated by using the case numbers for those days from the prior year (10 March 2022 through 04 May 2022). In almost all geographic regions, these extrapolations were comparable with the preceding observed case counts, suggesting that they are a reasonably good fit.

The final FOI score used for covariate adjustment is standardized to have empirical mean 0 and empirical standard deviation 1.

Exposure-proximal correlates analysis

First, each participant's $\log_{10}$ antibody titer trajectory was predicted based on the antibody decay model. We computed the expected $\log_{10}$ antibody for participants at daily increments conditional on their $\log_{10}$ antibody titer measurements prior to any breakthrough infection. Here, we obtained the fitted values conditional on the random intercept for each participant. Participants who were determined to have been asymptomatically infected between D22 and D91 had their trajectory from D92 onward predicted based on their D91 measurement and the rate of decay estimated based on baseline seropositive participants.

In the second step, a proportional hazards regression model was formulated. The operational time scale was calendar time, with March 30, 2022, taken to be time zero. Participants entered the risk set at D15 following their booster dose and were censored at D1 if they developed COVID-19 prior to D15. The hazard for the model for participant *i* is shown in Equation (2):

$$\lambda_i(t) = \lambda_0(t) \exp\left\{X_i^T \beta + \gamma \log_{10}\left(\widehat{Ab}_i\left(t - \tau_i^{D15}\right)\right)\right\} I\left\{t > \tau_i^{D15}\right\}, \quad (2)$$

where τ_i^{D15} is the calendar time of D15 post-booster for participant *i*, $\boldsymbol{\beta}$ is a vector of parameters whose values correspond to log hazard ratios for baseline covariates, and $\widehat{Ab}_i(t - \tau_i^{D15})$ is the predicted antibody for participant *i* at time $(t - \tau_i^{D15})$ post-D15. The calendar-time-based Cox analysis adjusted for the risk score.

Bootstrap confidence intervals and p-values were determined as follows. A Bayesian bootstrap with Dirichlet weights (α =1) was used, where the weights were generated separately by naive/non-naive status and multiplied by the number of naive/non-naive participants, respectively, to maintain the contribution from naive and non-naive individuals in the weighted datasets. For each of 1000 weighted versions of the analysis dataset, we a) train the antibody decay models, b) predict antibody titers for all relevant calendar days as described above, and c) estimate the exposure-proximal Cox proportional hazards model described above. We present percentile confidence interval estimates as in Equation (3):

$$\left(\exp\left(\widehat{\gamma_b^{0.025}}\right),\ \exp\left(\widehat{\gamma_b^{0.975}}\right)\right) \quad (3)$$

where $\widehat{\gamma_b^{0.025}}$ and $\widehat{\gamma_b^{0.975}}$ are the 2.5th and 97.5th percentiles of the bootstrap estimates of γ for a given exposure-proximal Cox model. We determine p-values as the smallest α for which 0 is not included in the 1 - α confidence interval for $\hat{\gamma}$. We also plot the estimated hazard ratio over the range of observed titers. The analysis was conducted among baseline naïve and non-naïve participants, separately.

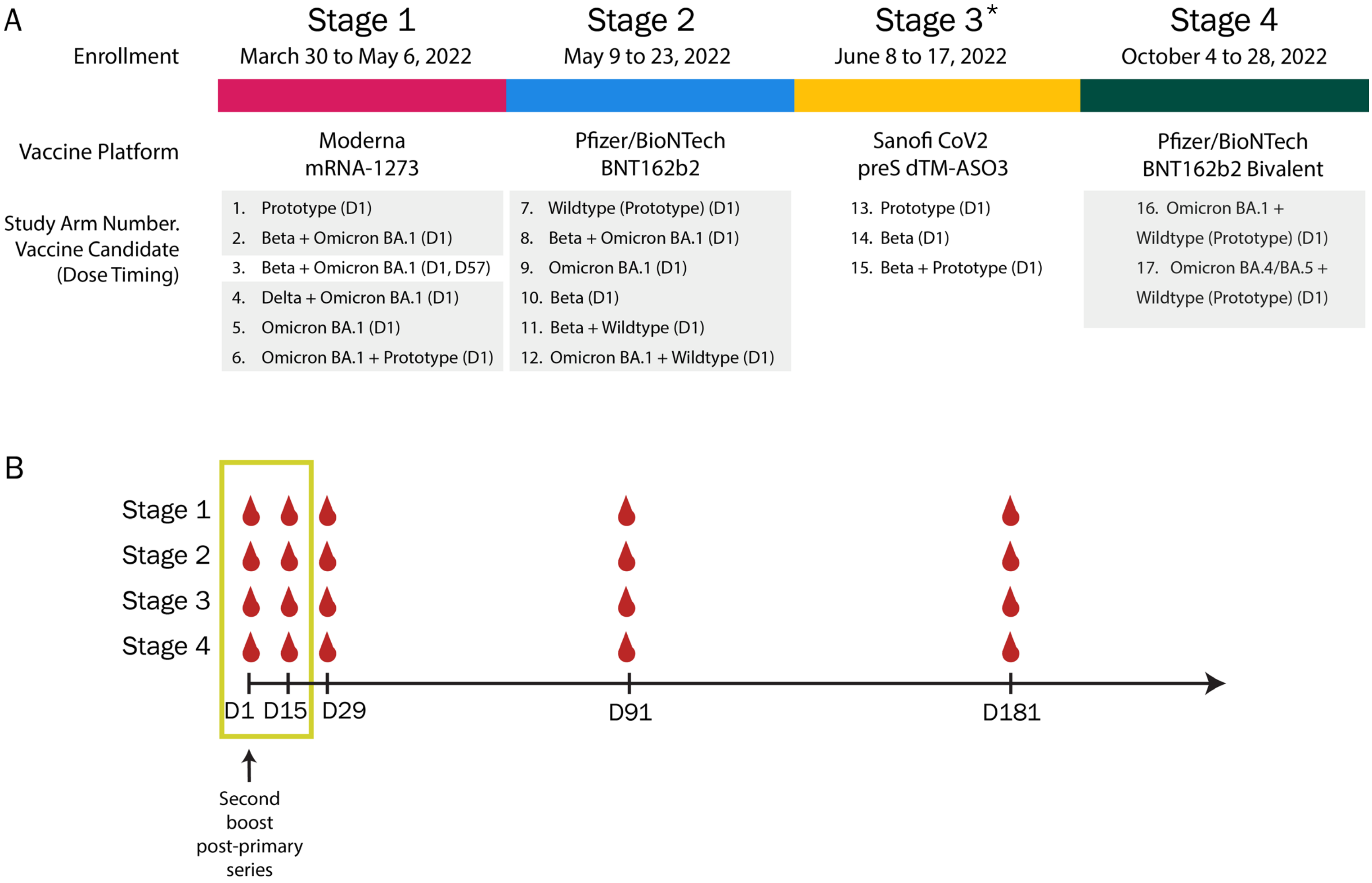

Supplementary Figure 1. COVAIL trial arms and sampling schema. A) Vaccine platform, study arm number, vaccine candidate(s), and dose (second boost post-primary series) timing for each of the four stages of the COVAIL trial. The 13 one-dose mRNA arms included in the present analysis are highlighted with a gray shaded box. B) Schematic showing serum sampling time points for measurement of immune markers for correlates studies. D1 = Day of receipt of the second boost post-primary series detailed in panel A. The vertical yellow rectangle outline highlights the two sampling visits (D1 and D15) most relevant for measuring neutralizing antibodies for the present work. The trial stages were conducted sequentially with span of enrollment dates noted.

*Note that data from Stage 3 of the study is not analyzed in the present study, but we include this stage in the overall schematic for completeness. The Stage 3 correlates results were described by Fong et al.[1]

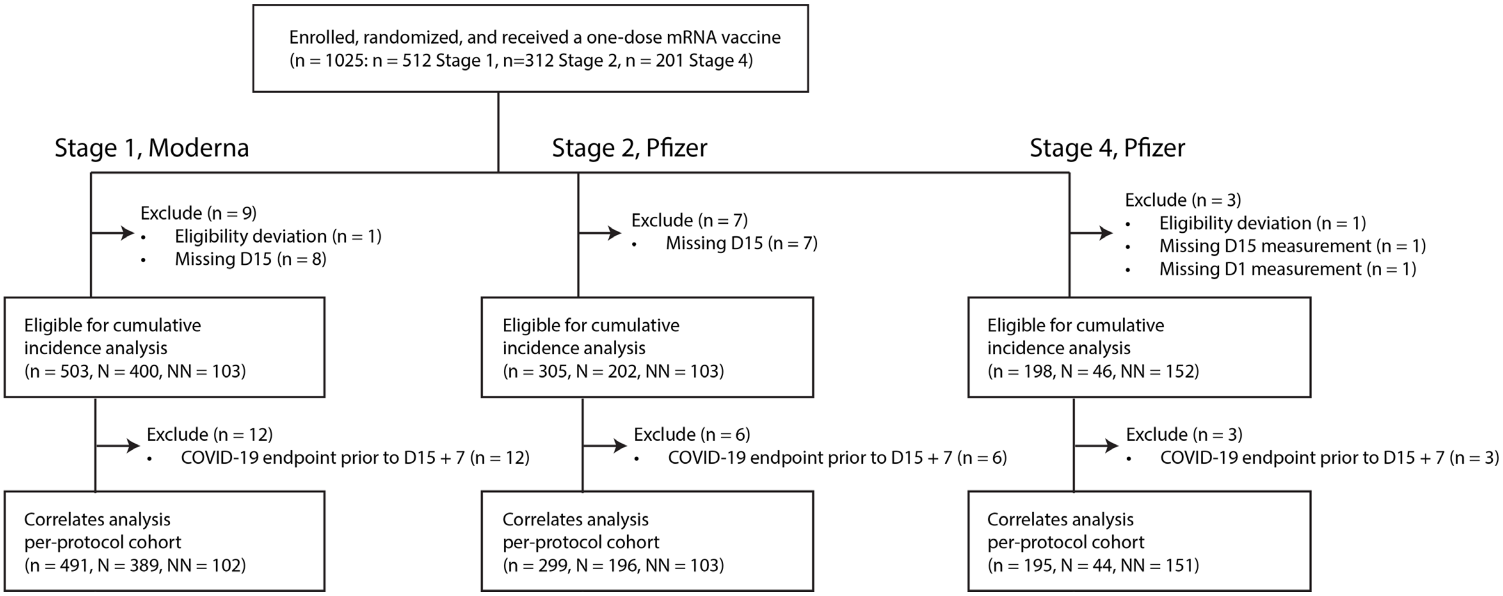

Supplementary Figure 2. Participant flow from enrollment, randomization, and receipt of a one-dose mRNA vaccine through to inclusion in the cumulative incidence analysis and then further through to inclusion in the correlates analysis per-protocol cohort. Availability of D1 or D15 measurements refers to BA.1 titer.

Supplementary Table 1. Demographic and clinical information for all study participants eligible for cumulative incidence analyses (N = 1006)

	1 Dose Beta + Omicron (Moderna)	1 Dose Delta + Omicron (Moderna)	1 Dose Omicron (Moderna)	1 Dose Omicron + Prototype (Moderna)	1 Dose Prototype (Moderna)	Beta (Pfizer-BioNTech 1)	Beta + Omicron (Pfizer-BioNTech 1)	Beta + Wildtype/Prototype (Pfizer-BioNTech 1)	Omicron (Pfizer-BioNTech 1)	Omicron + Wildtype/Prototype (Pfizer-BioNTech 1)	Wildtype/Prototype (Pfizer-BioNTech 1)	Omicron BA.1 + Wildtype/Prototype (Pfizer-BioNTech 2)	Omicron BA.4/5 + Wildtype/Prototype (Pfizer-BioNTech 2)
n	111	100	99	96	97	51	51	51	53	52	47	99	99
Age, yr (mean (SD))	52 (17)	52 (18)	52(17)	50 (18)	54 (17)	50 (16)	51 (18)	48 (18)	48 (16)	51 (16)	51 (18)	32 (8)	32 (9)
Age ≥ 65, n (%)	37 (33.3)	36 (36.0)	35 (35.4)	32 (33.3)	34 (35.1)	15 (29.4)	16 (31.4)	17 (33.3)	15 (28.3)	14 (26.9)	14 (29.8)	0 (0.0)	0 (0.0)
Sex													
Female, n (%)	64 (58)	51 (51)	53 (54)	49 (51)	49 (51)	28 (55)	26 (51)	27 (53)	30 (57)	30 (58)	24 (51)	55 (56)	56 (57)
Race, n (%)													
Asian	8 (7.2)	12 (12.0)	8 (8.1)	9 (9.4)	6 (6.2)	5 (9.8)	10 (19.6)	7 (13.7)	11 (20.8)	6 (11.5)	5 (10.6)	16 (16.2)	19 (19.2)
Black	7 (6.3)	7 (7.0)	4 (4.0)	9 (9.4)	12 (12.4)	5 (9.8)	3 (5.9)	7 (13.7)	4 (7.5)	3 (5.8)	5 (10.6)	11 (11.1)	12 (12.1)
American Indian or Alaska Native	3 (2.7)	3 (3.0)	1 (1.0)	1 (1.0)	1 (1.0)	1 (2.0)	0 (0.0)	0 (0.0)	0 (0.0)	0 (0.0)	2 (4.3)	2 (2.0)	2 (2.0)
Native Hawaiian or other Pacific Islander	0 (0.0)	0 (0.0)	0 (0.0)	0 (0.0)	0 (0.0)	1 (2.0)	0 (0.0)	0 (0.0)	0 (0.0)	0 (0.0)	0 (0.0)	1 (1.0)	0 (0.0)
White	94 (84.7)	88 (88.0)	87 (87.9)	77 (80.2)	79 (81.4)	41 (80.4)	40 (78.4)	39 (76.5)	41 (77.4)	44 (84.6)	36 (76.6)	73 (73.7)	68 (68.7)
Ethnicity, n (%)													
Not Hispanic or Latino	101 (91.0)	93 (93.0)	92 (92.9)	91 (94.8)	92 (94.8)	49 (96.1)	45 (88.2)	48 (94.1)	44 (83.0)	51 (98.1)	43 (91.5)	82 (82.8)	79 (79.8)
SARS-CoV-2-naïve, n (%)[1]	88 (79.3)	84 (84.0)	76 (76.8)	76 (79.2)	76 (78.4)	33 (64.7)	34 (66.7)	36 (70.6)	35 (66.0)	34 (65.4)	30 (63.8)	21 (21.2)	25 (25.3)
Force of Infection (FOI) standardized, mean (SD)	0.57 (0.65)	0.63 (0.63)	0.56 (0.64)	0.57 (0.59)	0.57 (0.69)	-0.06 (0.72)	-0.02 (0.68)	0.06 (0.63)	0.06 (0.69)	-0.01 (0.68)	-0.03 (0.61)	-1.53 (0.75)	-1.47 (0.74)
Risk score, mean (SD)	-1.42 (0.37)	-1.43 (0.35)	-1.42 (0.39)	-1.46 (0.40)	-1.48 (0.39)	-1.47 (0.32)	-1.44 (0.27)	-1.54 (0.37)	-1.42 (0.30)	-1.39 (0.31)	-1.49 (0.29)	-2.11 (0.51)	-2.02 (0.41)
Baseline (D1) titers (AU/ml), mean (SD)													
BA.1	2.51 (0.78)	2.43 (0.78)	2.54 (0.80)	2.55 (0.79)	2.43 (0.77)	2.43 (0.85)	2.49 (0.86)	2.58 (0.85)	2.52 (0.82)	2.56 (0.94)	2.65 (0.91)	3.05 (0.60)	3.00 (0.71)
BA.4/BA.5	2.24 (0.80)	2.17 (0.75)	2.29 (0.78)	2.24 (0.81)	2.20 (0.77)	2.17 (0.75)	2.19 (0.79)	2.19 (0.79)	2.28 (0.77)	2.28 (0.85)	2.34 (0.77)	2.89 (0.68)	2.86 (0.70)
Beta	3.05 (0.69)	2.92 (0.76)	2.97 (0.79)	2.98 (0.74)	2.92 (0.73)	2.99 (0.80)	3.02 (0.81)	3.08 (0.74)	2.95 (0.82)	3.03 (0.85)	3.12 (0.81)	3.45 (0.58)	3.45 (0.64)
Delta	3.29 (0.66)	3.25 (0.67)	3.28 (0.62)	3.25 (0.70)	3.21 (0.67)	3.27 (0.77)	3.24 (0.72)	3.37 (0.58)	3.25 (0.67)	3.31 (0.67)	3.38 (0.68)	3.53 (0.50)	3.56 (0.54)
D614G	3.61 (0.64)	3.54 (0.66)	3.59 (0.61)	3.56 (0.70)	3.52 (0.62)	3.57 (0.76)	3.57 (0.65)	3.66 (0.54)	3.60 (0.59)	3.67 (0.65)	3.70 (0.62)	3.88 (0.49)	3.89 (0.55)

Wt. Avg. [2]	2.81 (0.68)	2.73 (0.69)	2.81 (0.71)	2.80 (0.72)	2.73 (0.69)	2.75 (0.75)	2.78 (0.74)	2.84 (0.70)	2.80 (0.73)	2.85 (0.79)	2.92 (0.75)	3.27 (0.56)	3.26 (0.61)
Primary and first booster type (%)[3]													
J, J	0 (0.0)	1 (1.0)	0 (0.0)	2 (2.1)	0 (0.0)	0 (0.0)	0 (0.0)	0 (0.0)	0 (0.0)	0 (0.0)	0 (0.0)	1 (1.0)	1 (1.0)
J, M	4 (3.6)	4 (4.0)	3 (3.0)	1 (1.0)	3 (3.1)	1 (2.0)	0 (0.0)	0 (0.0)	0 (0.0)	2 (3.8)	0 (0.0)	2 (2.0)	4 (4.0)
J, P	2 (1.8)	1 (1.0)	1 (1.0)	0 (0.0)	1 (1.0)	0 (0.0)	0 (0.0)	0 (0.0)	0 (0.0)	0 (0.0)	0 (0.0)	4 (4.0)	2 (2.0)
M, M	37 (33.3)	37 (37.0)	34 (34.3)	25 (26.0)	32 (33.0)	12 (23.5)	19 (37.3)	14 (27.5)	13 (24.5)	8 (15.4)	22 (46.8)	29 (29.3)	34 (34.3)
M, P	6 (5.4)	4 (4.0)	3 (3.0)	6 (6.2)	3 (3.1)	3 (5.9)	2 (3.9)	3 (5.9)	3 (5.7)	3 (5.8)	3 (6.4)	6 (6.1)	5 (5.1)
P, M	8 (7.2)	6 (6.0)	6 (6.1)	8 (8.3)	5 (5.2)	2 (3.9)	1 (2.0)	6 (11.8)	7 (13.2)	3 (5.8)	1 (2.1)	7 (7.1)	9 (9.1)
P, P	54 (48.6)	47 (47.0)	52 (52.5)	54 (56.2)	53 (54.6)	33 (64.7)	29 (56.9)	28 (54.9)	30 (56.6)	36 (69.2)	21 (44.7)	50 (50.5)	44 (44.4)

[1]Naïve = Status of no previous COVID-19 infection at baseline, as determined by self-reported prior infection or positive N-Antibody test.

[2]Wt. Avg. = Maximum diversity weighted geometric mean of the five nAb titers D614G reference, Beta, Delta, Omicron BA.1, and Omicron BA.4/BA.5.

[3]Primary series and 1st booster vaccination: J, J = Johnson & Janssen 1 dose primary series and a Johnson & Janssen booster; J, M = Johnson & Janssen 1 dose primary series and a Moderna booster; J, P = Johnson & Janssen 1 dose primary series and a Pfizer-BioNTech booster; M, M = Moderna 2 dose primary series and a Moderna booster; M, P = Moderna 2 dose primary series and a Pfizer-BioNTech booster; P, M = Pfizer-BioNTech 2 dose primary series and a Moderna booster; P, P = Pfizer-BioNTech 2 dose primary series and a Pfizer-BioNTech booster.

The last 2 columns (“Pfizer-BioNTech 2” arms) were limited to participants 18-49 years of age and therefore there were no participants 65+ years in these arms.

Supplementary Table 2. Demographic and clinical information for baseline SARS-CoV-2 naive[1] study participants eligible for cumulative incidence analyses (N = 648)

	1 Dose Beta + Omicron (Moderna)	1 Dose Delta + Omicron (Moderna)	1 Dose Omicron (Moderna)	1 Dose Omicron + Prototype (Moderna)	1 Dose Prototype (Moderna)	Beta (Pfizer-BioNTech 1)	Beta + Omicron (Pfizer-BioNTech 1)	Beta + Wildtype/ Prototype (Pfizer-BioNTech 1)	Omicron (Pfizer-BioNTech 1)	Omicron + Wildtype/ Prototype (Pfizer-BioNTech 1)	Wildtype/ Prototype (Pfizer-BioNTech 1)	Omicron BA.1 + Wildtype/ Prototype (Pfizer-BioNTech 2)	Omicron BA.4/5 + Wildtype/ Prototype (Pfizer-BioNTech 2)
n	88	84	76	76	76	33	34	36	35	34	30	21	25
Age, yr (mean (SD))	52 (17)	53 (17)	53 (18)	50 (19)	55 (16)	50 (16)	51 (19)	50 (18)	51 (15)	53 (16)	54 (16)	35 (7.8)	34 (9.1)
Age ≥ 65, n (%)	31 (35.2)	33 (39.3)	31 (40.8)	28 (36.8)	29 (38.2)	11 (33.3)	12 (35.3)	13 (36.1)	11 (31.4)	11 (32.4)	11 (36.7)	0 (0.0)	0 (0.0)
Sex													
Female, n (%)	51 (58.0)	42 (50.0)	42 (55.3)	40 (52.6)	36 (47.4)	18 (54.5)	15 (44.1)	19 (52.8)	19 (54.3)	18 (52.9)	16 (53.3)	10 (47.6)	15 (60.0)
Race, n (%)													
Asian	6 (6.8)	11 (13.1)	4 (5.3)	7 (9.2)	5 (6.6)	3 (9.1)	7 (20.6)	6 (16.7)	7 (20.0)	4 (11.8)	2 (6.7)	3 (14.3)	5 (20.0)
Black	2 (2.3)	7 (8.3)	3 (3.9)	6 (7.9)	10 (13.2)	3 (9.1)	1 (2.9)	4 (11.1)	2 (5.7)	2 (5.9)	1 (3.3)	3 (14.3)	3 (12.0)
American Indian or Alaska Native	1 (1.1)	3 (3.6)	1 (1.3)	1 (1.3)	0 (0.0)	1 (3.0)	0 (0.0)	0 (0.0)	0 (0.0)	0 (0.0)	1 (3.3)	0 (0.0)	0 (0.0)
Native Hawaiian or other Pacific Islander	0 (0.0)	0 (0.0)	0 (0.0)	0 (0.0)	0 (0.0)	0 (0.0)	0 (0.0)	0 (0.0)	0 (0.0)	0 (0.0)	0 (0.0)	1 (4.8)	0 (0.0)
White	80 (90.9)	73 (86.9)	69 (90.8)	62 (81.6)	62 (81.6)	27 (81.8)	27 (79.4)	26 (72.2)	28 (80.0)	29 (85.3)	27 (90.0)	13 (61.9)	16 (64.0)
Ethnicity, n (%)													
Not Hispanic or Latino	82 (93.2)	79 (94.0)	70 (92.1)	72 (94.7)	73 (96.1)	31 (93.9)	29 (85.3)	36 (100.0)	29 (82.9)	34 (100.0)	29 (96.7)	18 (85.7)	21 (84.0)
Naive	88 (100.0)	84 (100.0)	76 (100.0)	76 (100.0)	76 (100.0)	33 (100.0)	34 (100.0)	36 (100.0)	35 (100.0)	34 (100.0)	30 (100.0)	21 (100.0)	25 (100.0)
Force of Infection (FOI) standardized, mean (SD)	0.60 (0.63)	0.65 (0.61)	0.58 (0.62)	0.59 (0.56)	0.58 (0.63)	-0.07 (0.78)	0.03 (0.71)	0.04 (0.61)	0.24 (0.62)	0.08 (0.69)	0.08 (0.63)	-1.43 (0.82)	-1.69 (0.60)
Risk score, mean (SD)	-1.39 (0.32)	-1.46 (0.36)	-1.41 (0.36)	-1.45 (0.38)	-1.47 (0.34)	-1.48 (0.32)	-1.48 (0.29)	-1.55 (0.35)	-1.44 (0.33)	-1.44 (0.33)	-1.52 (0.21)	-2.20 (0.67)	-1.94 (0.34)
Baseline (D1) titers (AU/ml), mean (SD)													
BA.1	2.33 (0.68)	2.33 (0.75)	2.31 (0.74)	2.36 (0.66)	2.22 (0.67)	2.09 (0.71)	2.11 (0.65)	2.27 (0.69)	2.20 (0.66)	2.14 (0.74)	2.15 (0.67)	2.48 (0.78)	2.21 (0.75)
BA.4/BA.5	2.04 (0.72)	2.03 (0.69)	2.07 (0.71)	2.03 (0.68)	1.98 (0.66)	1.88 (0.66)	1.83 (0.60)	1.86 (0.62)	1.95 (0.61)	1.94 (0.71)	1.91 (0.57)	2.10 (0.74)	2.09 (0.77)
Beta	2.88 (0.59)	2.83 (0.74)	2.77 (0.76)	2.82 (0.62)	2.72 (0.63)	2.70 (0.62)	2.70 (0.66)	2.83 (0.61)	2.67 (0.75)	2.66 (0.72)	2.69 (0.62)	2.88 (0.69)	2.80 (0.70)
Delta	3.17 (0.56)	3.15 (0.66)	3.14 (0.59)	3.12 (0.63)	3.05 (0.61)	3.01 (0.61)	2.98 (0.59)	3.17 (0.47)	3.03 (0.59)	3.01 (0.56)	3.02 (0.53)	3.04 (0.66)	3.04 (0.61)
D614G	3.49 (0.55)	3.45 (0.65)	3.46 (0.59)	3.43 (0.60)	3.36 (0.54)	3.35 (0.65)	3.35 (0.45)	3.51 (0.47)	3.40 (0.53)	3.39 (0.54)	3.36 (0.45)	3.45 (0.62)	3.39 (0.65)

Wt. Avg.[2]	2.64 (0.59)	2.62 (0.66)	2.62 (0.66)	2.62 (0.59)	2.53 (0.58)	2.46 (0.60)	2.45 (0.54)	2.58 (0.54)	2.52 (0.60)	2.49 (0.62)	2.49 (0.53)	2.68 (0.67)	2.58 (0.66)
Primary and first booster type (%)[3]													
J, J	0 (0.0)	1 (1.2)	0 (0.0)	0 (0.0)	0 (0.0)	0 (0.0)	0 (0.0)	0 (0.0)	0 (0.0)	0 (0.0)	0 (0.0)	0 (0.0)	0 (0.0)
J, M	3 (3.4)	4 (4.8)	2 (2.6)	1 (1.3)	2 (2.6)	0 (0.0)	0 (0.0)	0 (0.0)	0 (0.0)	1 (2.9)	0 (0.0)	0 (0.0)	3 (12.0)
J, P	1 (1.1)	1 (1.2)	0 (0.0)	0 (0.0)	0 (0.0)	0 (0.0)	0 (0.0)	0 (0.0)	0 (0.0)	0 (0.0)	0 (0.0)	2 (9.5)	1 (4.0)
M, M	29 (33.0)	33 (39.3)	28 (36.8)	20 (26.3)	26 (34.2)	8 (24.2)	14 (41.2)	10 (27.8)	8 (22.9)	6 (17.6)	13 (43.3)	5 (23.8)	11 (44.0)
M, P	5 (5.7)	3 (3.6)	2 (2.6)	6 (7.9)	1 (1.3)	3 (9.1)	2 (5.9)	3 (8.3)	2 (5.7)	1 (2.9)	2 (6.7)	2 (9.5)	1 (4.0)
P, M	8 (9.1)	3 (3.6)	6 (7.9)	8 (10.5)	5 (6.6)	0 (0.0)	1 (2.9)	5 (13.9)	6 (17.1)	1 (2.9)	0 (0.0)	3 (14.3)	2 (8.0)
P, P	42 (47.7)	39 (46.4)	38 (50.0)	41 (53.9)	42 (55.3)	22 (66.7)	17 (50.0)	18 (50.0)	19 (54.3)	25 (73.5)	15 (50.0)	9 (42.9)	7 (28.0)

[1]Naïve = Status of no previous COVID-19 infection at baseline, as determined by self-reported prior infection or positive N-Antibody test.

[2]Wt. Avg. = Maximum diversity weighted geometric mean of the five nAb titers D614G reference, Beta, Delta, Omicron BA.1, and Omicron BA.4/BA.5.

[3]Primary series and 1st booster vaccination: J, J = Johnson & Janssen 1 dose primary series and a Johnson & Janssen booster; J, M = Johnson & Janssen 1 dose primary series and a Moderna booster; J, P = Johnson & Janssen 1 dose primary series and a Pfizer-BioNTech booster; M, M = Moderna 2 dose primary series and a Moderna booster; M, P = Moderna 2 dose primary series and a Pfizer-BioNTech booster; P, M = Pfizer-BioNTech 2 dose primary series and a Moderna booster; P, P = Pfizer-BioNTech 2 dose primary series and a Pfizer-BioNTech booster.

*The last 2 columns (“Pfizer-BioNTech 2” arms) were limited to participants 18-49 years of age and therefore there were no participants 65+ years in these arms.

Supplementary Table 3. Demographic and clinical information for baseline SARS-CoV-2 non-naive[1] study participants eligible for cumulative incidence analyses (N = 358)

	1 Dose Beta + Omicron (Moderna)	1 Dose Delta + Omicron (Moderna)	1 Dose Omicron (Moderna)	1 Dose Omicron + Prototype (Moderna)	1 Dose Prototype (Moderna)	Beta (Pfizer-BioNTech 1)	Beta + Omicron (Pfizer-BioNTech 1)	Beta + Wildtype/ Prototype (Pfizer-BioNTech 1)	Omicron (Pfizer-BioNTech 1)	Omicron + Wildtype/ Prototype (Pfizer-BioNTech 1)	Wildtype/ Prototype (Pfizer-BioNTech 1)	Omicron BA.1 + Wildtype/ Prototype (Pfizer-BioNTech 2)	Omicron BA.4/5 + Wildtype/ Prototype (Pfizer-BioNTech 2)
N	23	16	23	20	21	18	17	15	18	18	17	78	74
Age, yr (mean (SD))	49 (18)	44 (19)	49 (14)	49 (17)	50 (18)	50 (16)	50 (15)	44 (18)	44 (18)	48 (15)	45 (19)	31 (8.0)	31 (8.7)
Age ≥ 65, n (%)	6 (26.1)	3 (18.8)	4 (17.4)	4 (20.0)	5 (23.8)	4 (22.2)	4 (23.5)	4 (26.7)	4 (22.2)	3 (16.7)	3 (17.6)	0 (0.0)	0 (0.0)
Sex													
Female, n (%)	13 (56.5)	9 (56.2)	11 (47.8)	9 (45.0)	13 (61.9)	10 (55.6)	11 (64.7)	8 (53.3)	11 (61.1)	12 (66.7)	8 (47.1)	45 (57.7)	41 (55.4)
Race, n (%)													
Asian	2 (8.7)	1 (6.2)	4 (17.4)	2 (10.0)	1 (4.8)	2 (11.1)	3 (17.6)	1 (6.7)	4 (22.2)	2 (11.1)	3 (17.6)	13 (16.7)	14 (18.9)
Black	5 (21.7)	0 (0.0)	1 (4.3)	3 (15.0)	2 (9.5)	2 (11.1)	2 (11.8)	3 (20.0)	2 (11.1)	1 (5.6)	4 (23.5)	8 (10.3)	9 (12.2)
American Indian or Alaska Native	2 (8.7)	0 (0.0)	0 (0.0)	0 (0.0)	1 (4.8)	0 (0.0)	0 (0.0)	0 (0.0)	0 (0.0)	0 (0.0)	1 (5.9)	2 (2.6)	2 (2.7)
Native Hawaiian or other Pacific Islander	0 (0.0)	0 (0.0)	0 (0.0)	0 (0.0)	0 (0.0)	1 (5.6)	0 (0.0)	0 (0.0)	0 (0.0)	0 (0.0)	0 (0.0)	0 (0.0)	0 (0.0)
White	14 (60.9)	15 (93.8)	18 (78.3)	15 (75.0)	17 (81.0)	14 (77.8)	13 (76.5)	13 (86.7)	13 (72.2)	15 (83.3)	9 (52.9)	60 (76.9)	52 (70.3)
Ethnicity, n (%)													
Not Hispanic or Latino	19 (82.6)	14 (87.5)	22 (95.7)	19 (95.0)	19 (90.5)	18 (100.0)	16 (94.1)	12 (80.0)	15 (83.3)	17 (94.4)	14 (82.4)	64 (82.1)	58 (78.4)
Non-naive	23 (100.0)	16 (100.0)	23 (100.0)	20 (100.0)	21 (100.0)	18 (100.0)	17 (100.0)	15 (100.0)	18 (100.0)	18 (100.0)	17 (100.0)	78 (100.0)	74 (100.0)
Force of Infection (FOI) standardized, mean (SD)	0.46 (0.72)	0.51 (0.72)	0.50 (0.70)	0.50 (0.68)	0.53 (0.88)	-0.05 (0.64)	-0.11 (0.63)	0.10 (0.69)	-0.29 (0.69)	-0.19 (0.66)	-0.23 (0.53)	-1.56 (0.74)	-1.40 (0.78)
Risk score, mean (SD)	-1.53 (0.50)	-1.27 (0.22)	-1.47 (0.47)	-1.51 (0.49)	-1.51 (0.55)	-1.45 (0.34)	-1.38 (0.20)	-1.53 (0.42)	-1.38 (0.24)	-1.29 (0.27)	-1.45 (0.39)	-2.08 (0.45)	-2.05 (0.43)
Baseline (D1) titers (AU/ml), mean (SD)													
BA.1	3.20 (0.72)	2.95 (0.73)	3.27 (0.54)	3.24 (0.89)	3.20 (0.63)	3.07 (0.71)	3.26 (0.72)	3.34 (0.73)	3.13 (0.76)	3.35 (0.76)	3.54 (0.51)	3.20 (0.44)	3.26 (0.45)
BA.4/BA.5	3.00 (0.64)	2.90 (0.62)	3.01 (0.52)	3.05 (0.78)	3.00 (0.63)	2.70 (0.61)	2.91 (0.59)	2.98 (0.57)	2.92 (0.63)	2.92 (0.74)	3.11 (0.40)	3.10 (0.47)	3.12 (0.43)
Beta	3.70 (0.66)	3.42 (0.62)	3.64 (0.50)	3.60 (0.82)	3.62 (0.66)	3.52 (0.82)	3.67 (0.72)	3.70 (0.69)	3.50 (0.68)	3.72 (0.60)	3.90 (0.41)	3.61 (0.44)	3.67 (0.44)
Delta	3.74 (0.83)	3.76 (0.44)	3.73 (0.45)	3.73 (0.79)	3.82 (0.56)	3.74 (0.81)	3.76 (0.70)	3.85 (0.55)	3.67 (0.61)	3.87 (0.50)	4.03 (0.36)	3.66 (0.36)	3.73 (0.38)
D614G	4.04 (0.77)	4.02 (0.46)	4.03 (0.42)	4.04 (0.84)	4.12 (0.53)	3.97 (0.82)	4.02 (0.77)	4.03 (0.52)	3.99 (0.51)	4.18 (0.50)	4.31 (0.35)	3.99 (0.38)	4.06 (0.39)

Wt. Avg.[2]	3.44 (0.66)	3.30 (0.57)	3.45 (0.46)	3.45 (0.81)	3.45 (0.58)	3.29 (0.70)	3.43 (0.67)	3.49 (0.61)	3.35 (0.64)	3.51 (0.64)	3.68 (0.37)	3.43 (0.40)	3.49 (0.39)
Primary and first booster type (%)[3]													
J, J	0 (0.0)	0 (0.0)	0 (0.0)	2 (10.0)	0 (0.0)	0 (0.0)	0 (0.0)	0 (0.0)	0 (0.0)	0 (0.0)	0 (0.0)	1 (1.3)	1 (1.4)
J, M	1 (4.3)	0 (0.0)	1 (4.3)	0 (0.0)	1 (4.8)	1 (5.6)	0 (0.0)	0 (0.0)	0 (0.0)	1 (5.6)	0 (0.0)	2 (2.6)	1 (1.4)
J, P	1 (4.3)	0 (0.0)	1 (4.3)	0 (0.0)	1 (4.8)	0 (0.0)	0 (0.0)	0 (0.0)	0 (0.0)	0 (0.0)	0 (0.0)	2 (2.6)	1 (1.4)
M, M	8 (34.8)	4 (25.0)	6 (26.1)	5 (25.0)	6 (28.6)	4 (22.2)	5 (29.4)	4 (26.7)	5 (27.8)	2 (11.1)	9 (52.9)	24 (30.8)	23 (31.1)
M, P	1 (4.3)	1 (6.2)	1 (4.3)	0 (0.0)	2 (9.5)	0 (0.0)	0 (0.0)	0 (0.0)	1 (5.6)	2 (11.1)	1 (5.9)	4 (5.1)	4 (5.4)
P, M	0 (0.0)	3 (18.8)	0 (0.0)	0 (0.0)	0 (0.0)	2 (11.1)	0 (0.0)	1 (6.7)	1 (5.6)	2 (11.1)	1 (5.9)	4 (5.1)	7 (9.5)
P, P	12 (52.2)	8 (50.0)	14 (60.9)	13 (65.0)	11 (52.4)	11 (61.1)	12 (70.6)	10 (66.7)	11 (61.1)	11 (61.1)	6 (35.3)	41 (52.6)	37 (50.0)

[1]Non-naïve = Status of previous COVID-19 infection at baseline, as determined by self-reported prior infection or positive N-Antibody test.

[2]Wt. Avg. = Maximum diversity weighted geometric mean of the five nAb titers D614G reference, Beta, Delta, Omicron BA.1, and Omicron BA.4/BA.5.

[3]Primary series and 1st booster vaccination: J, J = Johnson & Janssen 1 dose primary series and a Johnson & Janssen booster; J, M = Johnson & Janssen 1 dose primary series and a Moderna booster; J, P = Johnson & Janssen 1 dose primary series and a Pfizer-BioNTech booster; M, M = Moderna 2 dose primary series and a Moderna booster; M, P = Moderna 2 dose primary series and a Pfizer-BioNTech booster; P, M = Pfizer-BioNTech 2 dose primary series and a Moderna booster; P, P = Pfizer-BioNTech 2 dose primary series and a Pfizer-BioNTech booster.

*The last 2 columns ("Pfizer-BioNTech 2" arms) were limited to participants 18-49 years of age and therefore there were no participants 65+ years in these arms.

Supplementary Table 4. Clinical and Supportive Laboratory Criteria for Centers for Diseases Control and Prevention National Notifiable Disease Surveillance System Coronavirus Disease 2019 (COVID-19) 2021 Case Definition.[2] All COVID-19 endpoints included in the current analysis met these clinical and supportive laboratory criteria.

Clinical Criteria

In the absence of a more likely diagnosis:

- Acute onset or worsening of at least two of the following symptoms or signs:
    - fever (measured or subjective),
    - chills,
    - rigors,
    - myalgia,
    - headache,
    - sore throat,
    - nausea or vomiting,
    - diarrhea,
    - fatigue,
    - congestion or runny nose.

OR

- Acute onset or worsening of any one of the following symptoms or signs:
    - cough,
    - shortness of breath,
    - difficulty breathing,
    - olfactory disorder,
    - taste disorder,
    - confusion or change in mental status,
    - persistent pain or pressure in the chest,
    - pale, gray, or blue-colored skin, lips, or nail beds, depending on skin tone,
    - inability to wake or stay awake.

OR

- Severe respiratory illness with at least one of the following:
    - Clinical or radiographic evidence of pneumonia,
    - Acute respiratory distress syndrome (ARDS).

Laboratory Criteria

Supportive laboratory evidence:

- Detection of antibody in serum, plasma, or whole blood specific to natural infection with SARS-CoV-2 (antibody to nucleocapsid protein), **OR**
- Detection of SARS-CoV-2 specific antigen by immunocytochemistry in an autopsy specimen, **OR**
- Detection of SARS-CoV-2 RNA or specific antigen using a test performed without CLIA oversight.

Supplementary Table 5. Numbers of COVID-19 endpoints in the COVID-19 cumulative incidence analysis cohort (see Supplementary Fig. 2), shown by study stage, time period of endpoint occurrence, and naïve/non-naïve status.

			COVID-19 Endpoints			
Study Stage	Prior SARS-CoV-2 Infection	Total Participants	Early	Booster-Proximal*	Booster-Distal*	Total
Stage 1	Naïve	400	11	78	49	138
Stage 1	Non-Naïve	103	1	10	6	17
Stage 2	Naïve	202	6	35	10	51
Stage 2	Non-Naïve	103	0	7	3	10
Stage 4	Naïve	46	2	9	0	11
Stage 4	Non-Naïve	152	1	5	1	7

*Only booster-proximal and booster-distal COVID-19 endpoints were included in the correlates analyses.

Naïve/Non-naïve Definitions: A participant was determined to be SARS-CoV-2 non-naïve at baseline if they self-reported a previous SARS-CoV-2 infection or had detectable anti-N antibodies at D1 (Elecsys Anti-SARS-CoV-2 N; Roche). Otherwise, a participant was considered SARS-CoV-2 naïve.

COVID-19 Endpoint: As in Branche et al.,[3] a self-reported positive SARS-CoV-2 test or study-conducted positive SARS-CoV-2 test, with onset date the earliest positive test date.

Time Period of COVID-19 Endpoint Occurrence:

- Early: After booster and prior to 7 days post D15 visit
- Booster-Proximal: 7 to 91 days after D15
- Booster-Distal: 92 to 188 days after D15

Supplementary Table 6. Numbers and percentages of COVID-19 endpoints, by naïve/non-naïve status, with each of 22 different symptoms.

	**Naïve, count**	**Naïve, %**	**Non-naïve, count**	**Non-naïve, %**
Number of endpoints	**181**		**32**	
Total # symptoms (average per case)	6.3		5.9	
Satisfying CDC clinical criteria[2]	176	97.2%	32	100%
Satisfying COVE clinical criteria[4]	162	89.5%	29	90.6%
**Symptom**				
Congestion	146	0.81	22	0.69
Cough	144	0.80	25	0.78
Runny nose	133	0.73	20	0.63
Sore throat	132	0.73	25	0.78
Fatigue	124	0.69	20	0.63
Headache	101	0.56	18	0.56
Muscle aches	78	0.43	14	0.44
Body aches	77	0.43	15	0.47
Fever	53	0.29	7	0.22
Chill	52	0.29	4	0.13
Diarrhea	16	0.09	2	0.06
Nausea	15	0.08	6	0.19
Shortness of breath	15	0.08	2	0.06
Rigor	13	0.07	0	0.00
Loss of smell	13	0.07	2	0.06
Loss of taste	10	0.06	2	0.06
Difficulty breathing	4	0.02	0	0.00
Confusion	4	0.02	3	0.09
Trouble waking	4	0.02	0	0.00
Vomit	3	0.02	0	0.00
Chest pain	2	0.01	1	0.03

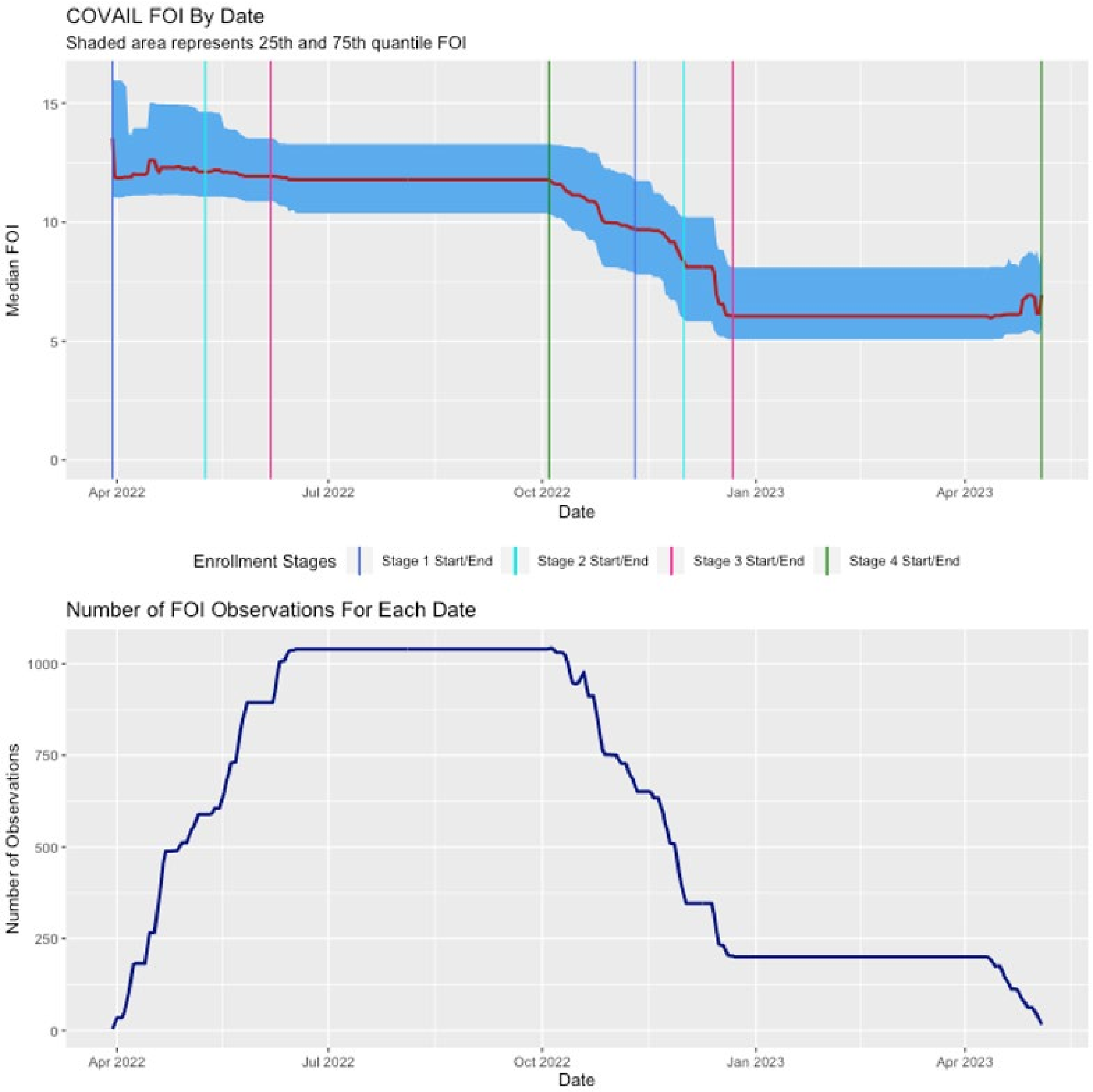

Supplementary Figure 3. Top panel: Distribution of median FOI scores (red line) of trial participants across calendar time and trial stage. The blue shaded area represents the 25th and 75th quantile FOI score. Bottom panel: Number of participants with FOI observations for each date. FOI, force of infection.

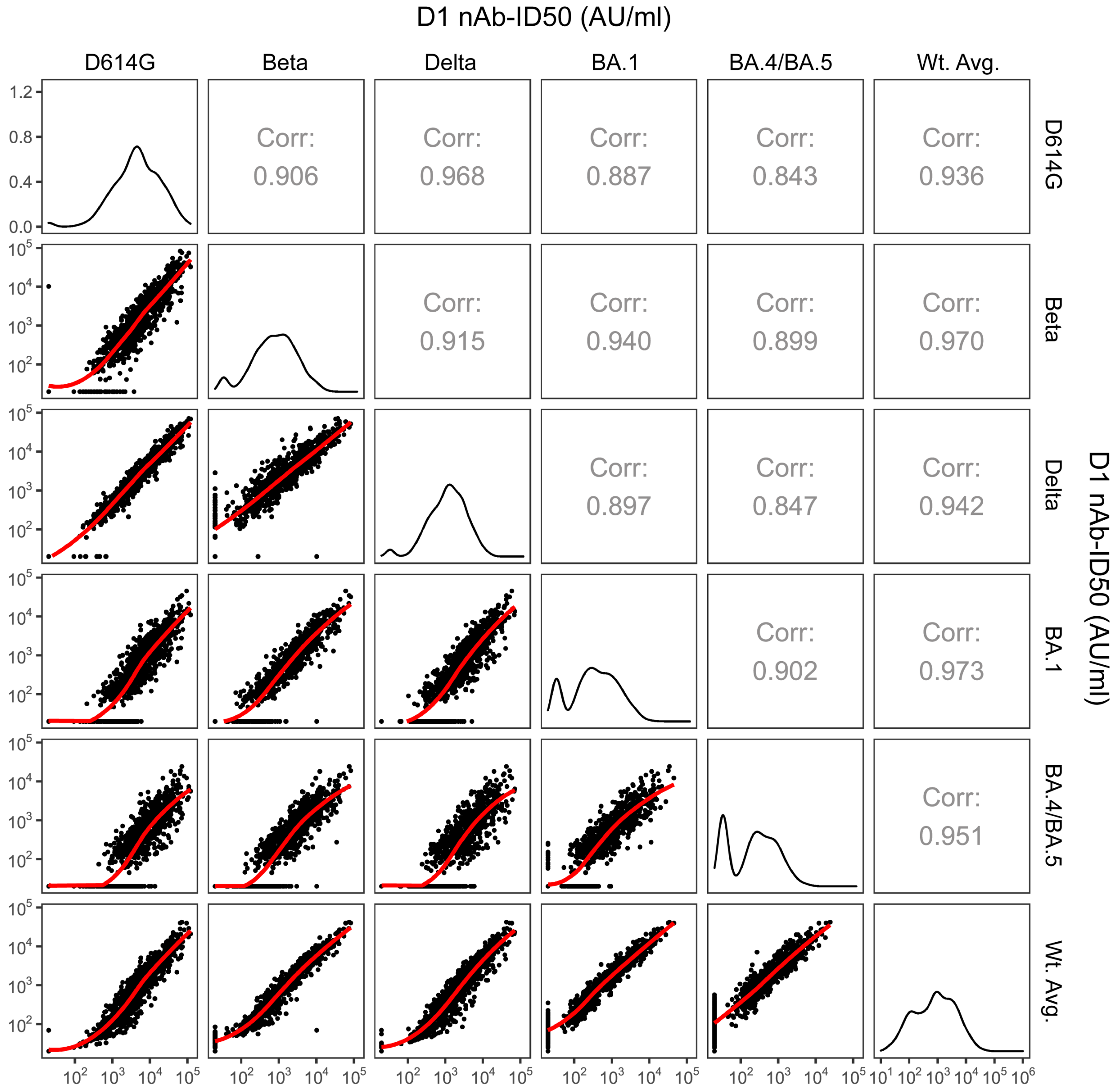

Supplementary Figure 4. Correlations of D1 nAb-ID50 titers, among participants pooled across 13 one-dose mRNA booster arms. The red lines are smooth curves delineating the relationship between the two variables and were fitted using the LOESS method/local regression method. AU, arbitrary units; Corr = Spearman rank correlation coefficient; nAb-ID50, 50% inhibitory dilution neutralizing antibody titer.

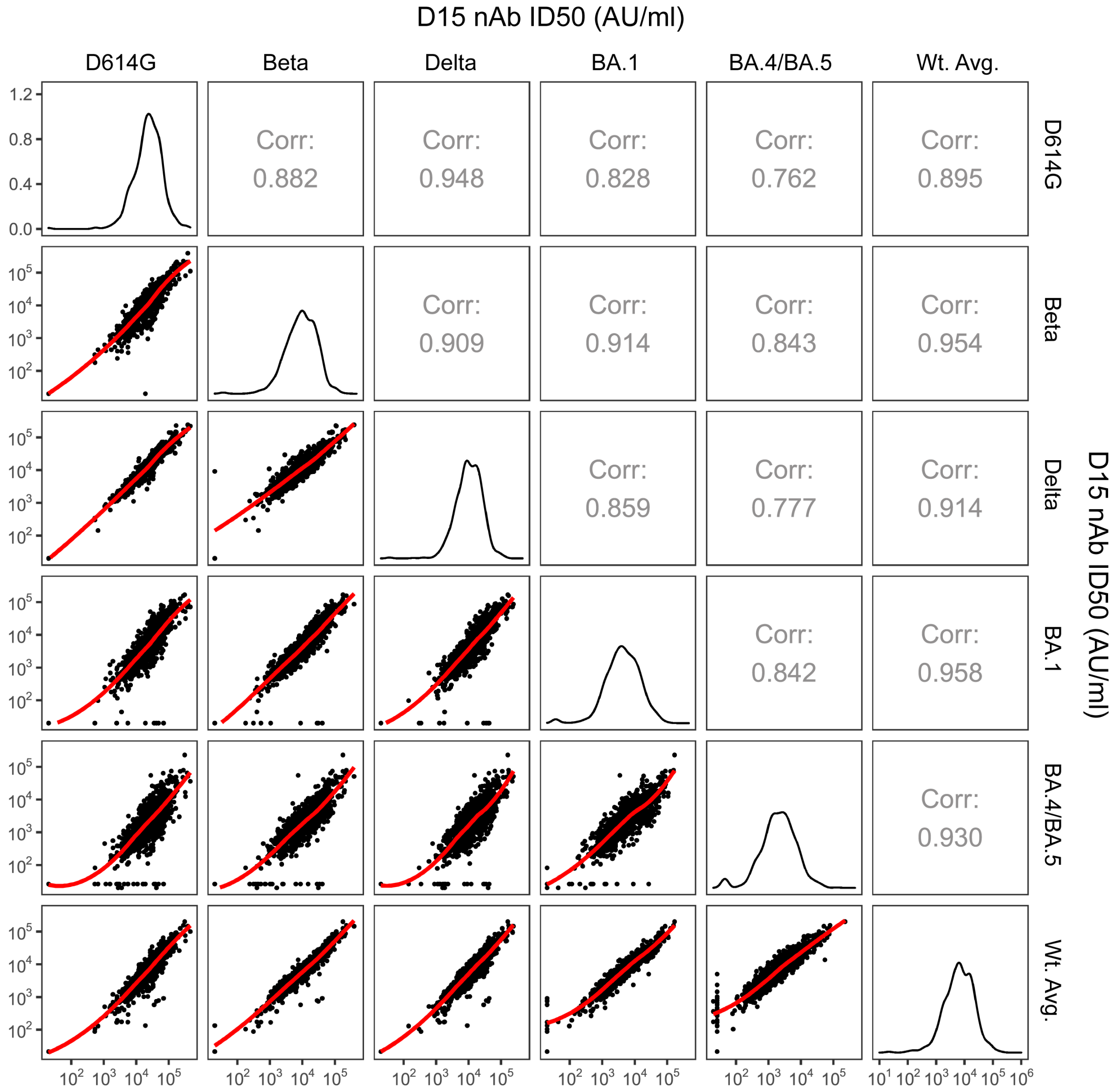

Supplementary Figure 5. Correlations of D15 nAb-ID50 titers, among participants pooled across 13 one-dose mRNA booster arms. The red lines are smooth curves delineating the relationship between the two variables and were fitted using the LOESS method/local regression method. AU, arbitrary units; Corr = Spearman rank correlation coefficient; nAb-ID50, 50% inhibitory dilution neutralizing antibody titer.

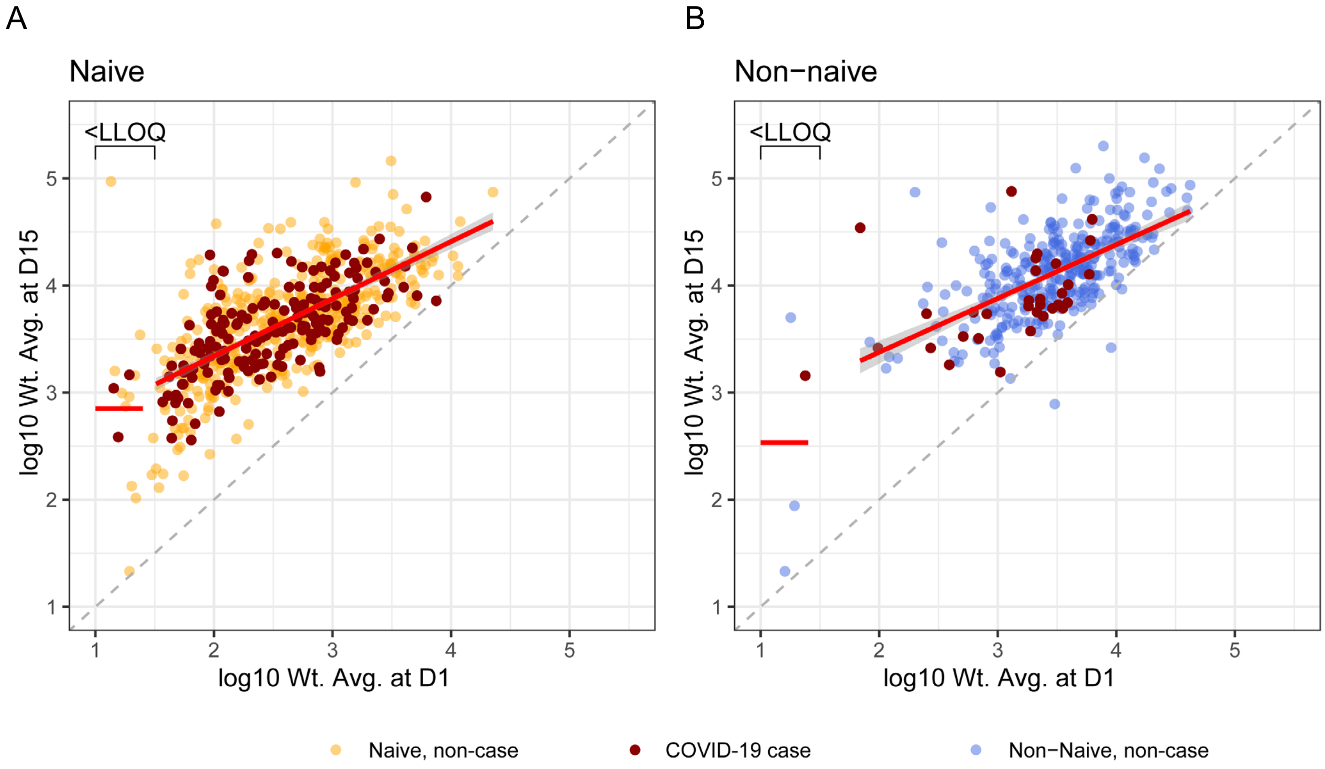

Supplementary Figure 6. Scatterplots of D1 and D15 log10 weighted average titers in (A) naïve participants (N=629; 181 cases and 448 non-cases) and (B) non-naïve participants (N=356; 32 cases and 324 non-cases). The short horizontal red line represents the mean of log10 Wt. Avg. values that are <LLOQ at Day15. The diagonal red line represents the fitted linear regression line between Day1 and Day15 log10 Wt. Avg. values that are above LLOQ. The gray area around the diagonal red line indicates the 95% confidence interval of the fitted line. Red dots identify COVID-19 cases from 7 to 188 days post D15 visit; yellow dots identify naïve non-cases; blue dots identify non-naïve non-cases. Wt. Avg. = Maximum diversity weighted geometric mean of the five neutralizing antibody titers D614G, Beta, Delta, BA.1, and BA.4/BA.5. LLOQ, lower limit of quantification.

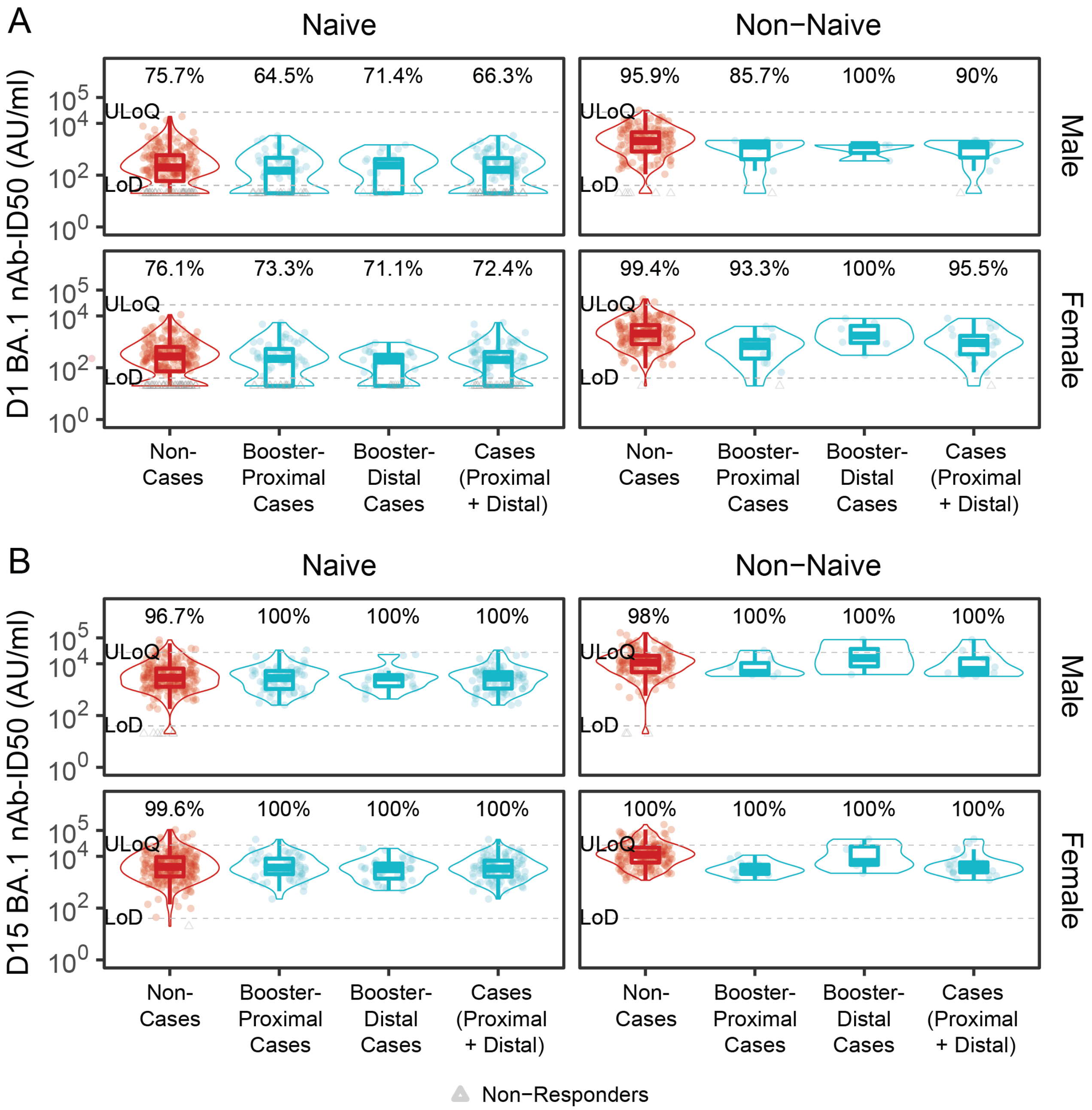

Supplementary Figure 7. Results of a post hoc analysis showing violin box plots of A) D1 and B) D15 BA.1 ID50 titers, shown by non-cases (red dots) and COVID-19 cases (turquoise dots) and stratified by booster-proximal cases, booster-distal-cases, and proximal+distal cases, presented separately for SARS-CoV-2 naïve and non-naïve participants, as well as separately for males and for females. Data points are shown for the following numbers of participants: A) Naïve male: 214 non-cases; 62 booster-proximal cases, 21 booster-distal cases, 83 total cases; Naïve female: 234 non-cases; 60 booster-proximal cases, 38 booster-distal cases, 98 total cases; Non-Naïve male: 147 non-cases; 7

booster-proximal cases, 3 booster-distal cases, 10 total cases; Non-Naïve female: 177 non-cases; 15 booster-proximal cases, 7 booster-distal cases, 22 total cases. B) A) Naïve male: 214 non-cases; 62 booster-proximal cases, 21 booster-distal cases, 83 total cases; Naïve female: 234 non-cases; 60 booster-proximal cases, 38 booster-distal cases, 98 total cases; Non-Naïve male: 147 non-cases; 7 booster-proximal cases, 3 booster-distal cases, 10 total cases; Non-Naïve female: 177 non-cases; 15 booster-proximal cases, 7 booster-distal cases, 22 total cases. Violin plots contain interior box plots with upper and lower horizontal edges the 25th and 75th percentiles of antibody level and middle line the 50th percentile, and vertical bars the distance from the 25th (or 75th) percentile of antibody level and the minimum (or maximum) antibody level within the 25th (or 75th) percentile of antibody level minus (or plus) 1.5 times the interquartile range. Each side shows a rotated probability density (estimated by a kernel density estimator with a default Gaussian kernel) of the data. Non-cases: No evidence of SARS-CoV-2 infection at 7 days post D15 through to 188 days post D15. Booster-proximal cases: COVID-19 endpoint between 7 and 91 days post D15 visit; booster-distal cases: COVID-19 endpoint between 92 and 188 days post D15 visit; cases (proximal + distal): COVID-19 endpoint between 7 and 188 days post D15 visit. Numbers above each plot are percentages of participants with positive response, with positive response defined as titer above the limit of detection (LoD), 40 AU/ml. Gray triangles identify non-responders/undetectable. The upper dashed horizontal line in each plot shows the antigen-specific upper limit of quantification (ULoQ) and the lower dashed horizontal line in each plot shows the LoD. nAb-ID50, 50% inhibitory dilution neutralizing antibody titer. Source data are provided as a Source Data file.

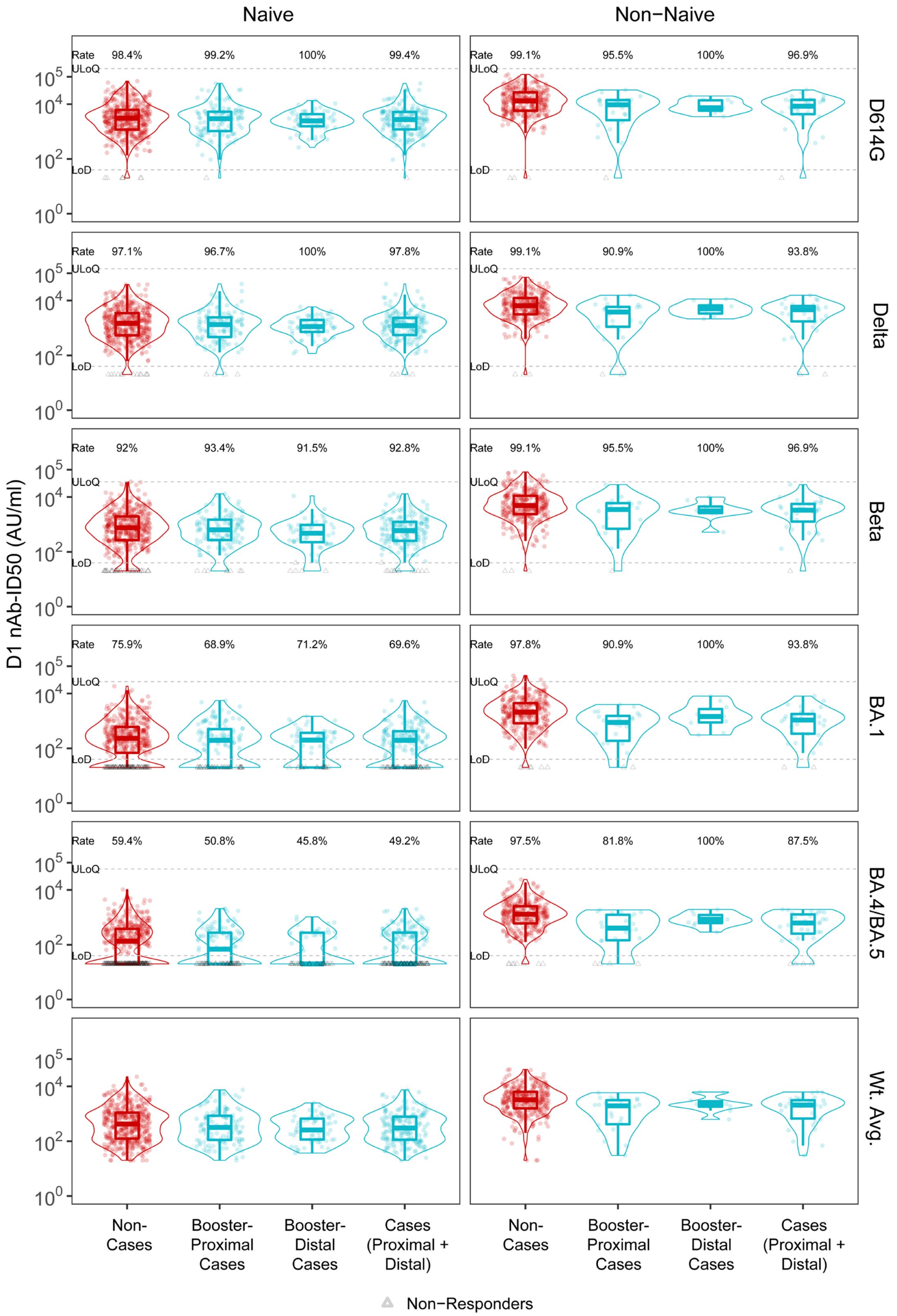

Supplementary Figure 8. Violin box plots of D1 levels for the six titer markers (D614G, Delta, Beta, BA.1, BA.4/BA.5, weighted average), shown by non-cases (red dots) and COVID-19 cases (turquoise dots) (stratified by booster-proximal cases, booster-distal cases, and proximal+distal cases), pooled across the 13 one-dose mRNA booster arms. Violin plots contain interior box plots with upper and lower horizontal edges the 25th and 75th percentiles of antibody level and middle line the 50th percentile, and vertical bars the distance from the 25th (or 75th) percentile of antibody level and the minimum (or

maximum) antibody level within the 25th (or 75th) percentile of antibody level minus (or plus) 1.5 times the interquartile range. Each side shows a rotated probability density (estimated by a kernel density estimator with a default Gaussian kernel) of the data. Non-cases: No evidence of SARS-CoV-2 infection at 7 days post D15 through to 188 days post D15 visit. Booster-proximal cases: COVID-19 endpoint between 7 and 91 days post D15 visit; booster-distal cases: COVID-19 endpoint between 92 and 188 days post D15 visit; cases (proximal + distal): COVID-19 endpoint between 7 and 188 days post D15 visit. Rate is the percentage of participants with positive response, with positive response defined as ID50 titer above the limit of detection (LoD), 40 AU/ml. Gray triangles identify non-responders/undetectable. The upper dashed horizontal line in each plot shows the antigen-specific upper limit of quantification (ULoQ) and the lower dashed horizontal line in each plot shows the LoD. AU, arbitrary units; nAb-ID50, 50% inhibitory dilution neutralizing antibody titer. Source data are provided as a Source Data file.

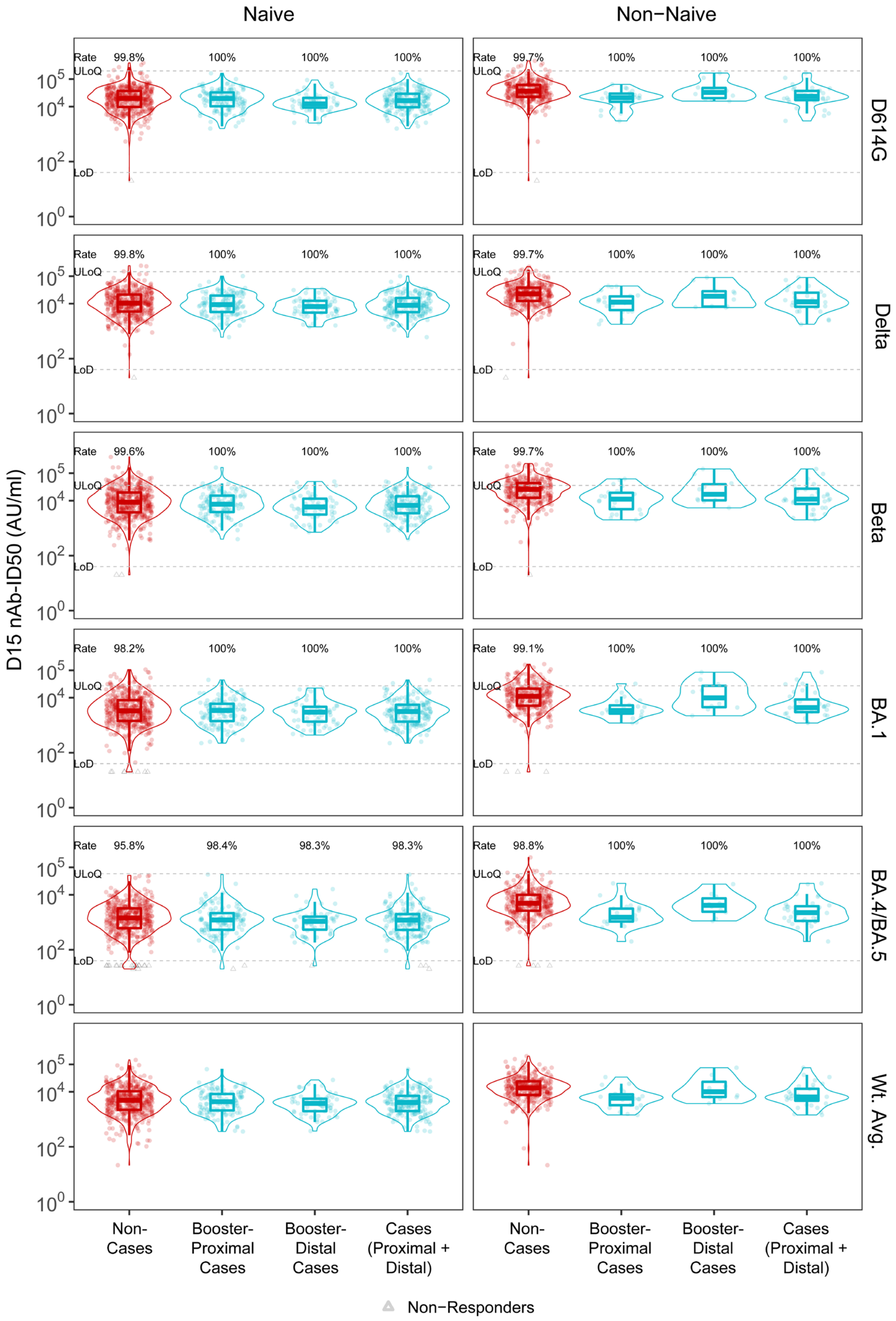

Supplementary Figure 9. Violin box plots of D15 levels for the six titer markers (D614G, Delta, Beta, BA.1, BA.4/BA.5, weighted average), shown by non-cases (red dots) and COVID-19 cases (turquoise dots) (stratified by booster-proximal cases, booster-distal cases, and proximal+distal cases), pooled across the 13 one-dose mRNA booster arms. Violin plots contain interior box plots with upper and lower horizontal edges the 25th and 75th percentiles of antibody level and middle line the 50th percentile, and vertical bars the distance from the 25th (or 75th) percentile of antibody level and the minimum (or

maximum) antibody level within the 25th (or 75th) percentile of antibody level minus (or plus) 1.5 times the interquartile range. Each side shows a rotated probability density (estimated by a kernel density estimator with a default Gaussian kernel) of the data. Non-cases: No evidence of SARS-CoV-2 infection at 7 days post D15 through to 188 days post D15 visit. Booster-proximal cases: COVID-19 endpoint between 7 and 91 days post D15 visit; booster-distal cases: COVID-19 endpoint between 92 and 188 days post D15 visit; cases (proximal + distal): COVID-19 endpoint between 7 and 188 days post D15 visit. Rate is the percentage of participants with positive response, with positive response defined as ID50 titer above the limit of detection (LoD), 40 AU/ml. Gray triangles identify non-responders/undetectable. The upper dashed horizontal line in each plot shows the antigen-specific upper limit of quantification (ULoQ) and the lower dashed horizontal line in each plot shows the LoD. AU, arbitrary units; nAb-ID50, 50% inhibitory dilution neutralizing antibody titer. Source data are provided as a Source Data file.

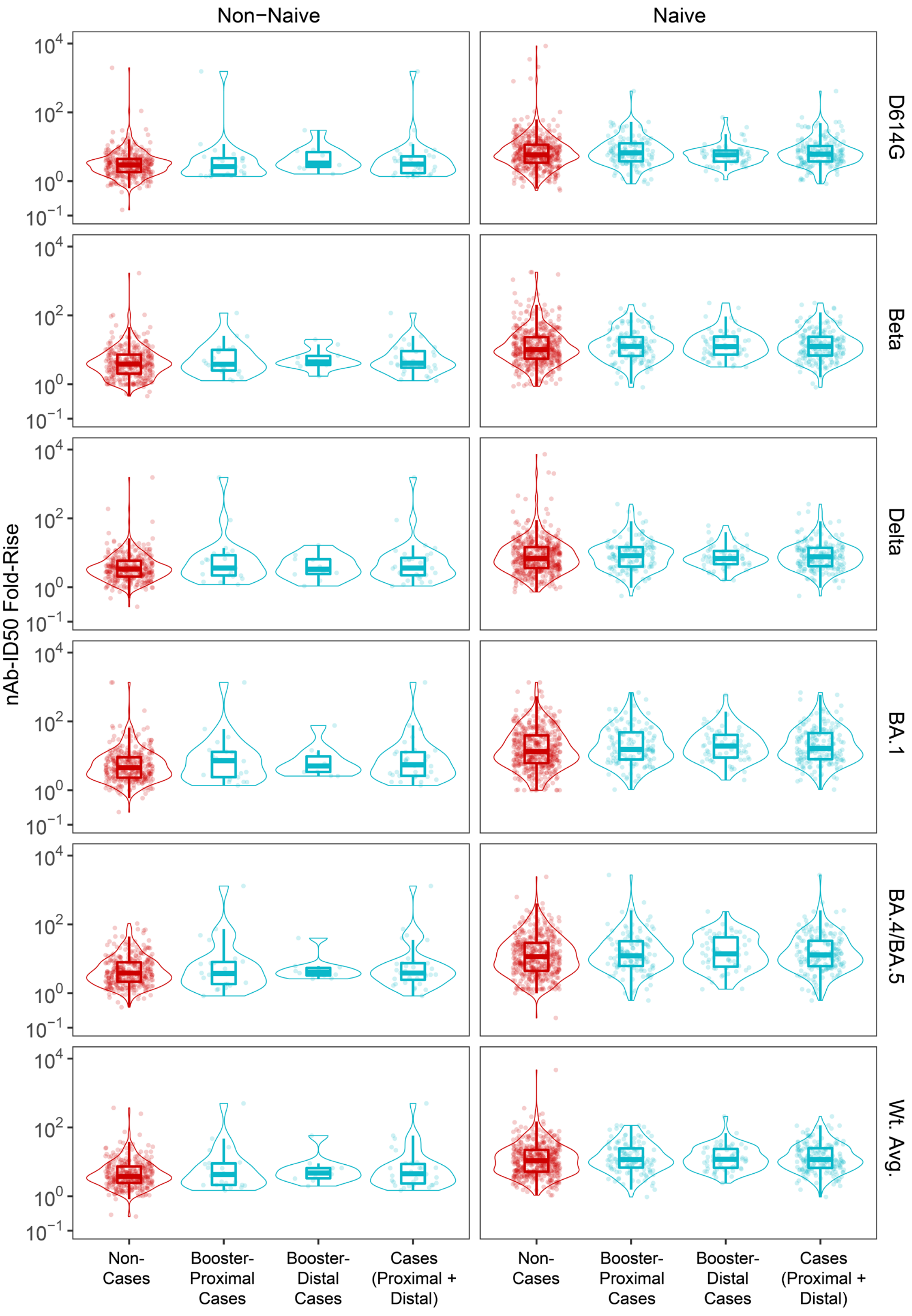

Supplementary Figure 10. Violin box plots of fold-rise levels for the six titer markers (D614G, Beta, Delta, BA.1, BA.4/BA.5, weighted average), shown by non-cases (red dots) and COVID-19 cases (turquoise dots) (stratified by booster-proximal cases, booster-distal cases, and proximal+distal cases), pooled across the 13 one-dose mRNA booster arms. Violin plots contain interior box plots with upper and lower horizontal edges the 25th and 75th percentiles of antibody level and middle line the 50th percentile, and vertical bars the distance from the 25th (or 75th) percentile of antibody level and the minimum (or

maximum) antibody level within the 25th (or 75th) percentile of antibody level minus (or plus) 1.5 times the interquartile range. Each side shows a rotated probability density (estimated by a kernel density estimator with a default Gaussian kernel) of the data. Non-cases: No evidence of SARS-CoV-2 infection at 7 days post D15 through to 188 days post D15 visit. Booster-proximal cases: COVID-19 endpoint between 7 and 91 days post D15 visit; booster-distal cases: COVID-19 endpoint between 92 and 188 days post D15 visit; cases (proximal + distal): COVID-19 endpoint between 7 and 188 days post D15 visit. AU, arbitrary units; nAb-ID50, 50% inhibitory dilution neutralizing antibody titer. Source data are provided as a Source Data file.

Supplementary Table 7. Interaction two-sided Wald p values (~ naïve + each peak titer + naïve * each peak titer, with and without additional covariate adjustment of the risk score and the FOI score) for whether naïve status modifies the hazard ratios of COVID-19 per 1 SD increase in each titer marker based on the entire correlates per-protocol cohort. No multiple hypothesis testing adjustment was made. HR Ratio refers to the ratio of the HR for naïves and the HR for non-naives.

		Boost Proximal					Entire Period				
			Without Cov. Adjust.		With Cov. Adj.			Without Cov. Adjust.		With Cov. Adj.	
	Sample size	Endpoints	HR Ratio	p	HR Ratio	p	Endpoints	HR Ratio	p	HR Ratio	p
BA.1	985	144	1.632	0.004	1.632	0.004	213	1.414	0.028	1.402	0.028
Beta	985	144	1.555	0.009	1.538	0.01	213	1.343	0.05	1.336	0.052
Delta	985	144	1.544	0.006	1.543	0.006	213	1.328	0.042	1.341	0.033
D614G	985	144	1.506	0.005	1.506	0.005	213	1.266	0.071	1.278	0.058
BA.4/BA.5	985	144	1.818	0.002	1.836	0.002	213	1.532	0.014	1.511	0.018
MDW	985	144	1.691	0.002	1.679	0.002	213	1.418	0.025	1.413	0.025

Supplementary Table 8. Interaction two-sided Wald p values (~ naïve + each peak titer + naïve * each peak titer, with and without additional covariate adjustment of the risk score and the FOI score) for whether naïve status modifies the hazard ratios of COVID-19 per 1 SD increase in each marker based on the entire correlates per-protocol cohort whose D15 titers are well-overlapped (intersection of middle 90% of naïve participants and the middle 90% of non-naïve participants for each D15 titer marker). No multiple hypothesis testing adjustment was made. HR Ratio refers to the ratio of the HR for naïves and the HR for non-naives.

		Boost Proximal					Entire Period				
			Without Cov. Adjust.		With Cov. Adj.			Without Cov. Adjust.		With Cov. Adj.	
	Sample size	Endpoints	HR Ratio	p	HR Ratio	p	Endpoints	HR Ratio	p	HR Ratio	p
BA.1	765	103	2.211	0.005	1.632	0.004	150	1.511	0.057	1.402	0.028
Beta	789	106	1.350	0.256	1.538	0.010	154	1.163	0.479	1.336	0.052
Delta	780	107	1.662	0.068	1.543	0.006	157	1.399	0.139	1.341	0.033
D614G	781	109	1.674	0.071	1.506	0.005	160	1.239	0.347	1.278	0.058
BA.4/BA.5	741	97	2.427	0.004	1.836	0.002	144	1.525	0.076	1.511	0.018
MDW	753	99	2.113	0.011	1.679	0.002	148	1.504	0.081	1.413	0.025

Supplementary Table 9. Interaction two-sided Wald p values (~ naïve + each peak titer + naïve * each peak titer, with and without additional covariate adjustment of the risk score and the FOI score) for whether naïve status modifies the hazard ratios of COVID-19 per 1 SD increase in each titer marker based on the correlates per-protocol cohort restricting to participants with detectable D1 titers. No multiple hypothesis testing adjustment was made.

		Boost Proximal					Entire Period				
			Without Cov. Adjust.		With Cov. Adj.			Without Cov. Adjust.		With Cov. Adj.	
	Sample size	Endpoints	HR Ratio	p	HR Ratio	p	Endpoints	HR Ratio	p	HR Ratio	p
BA.1	813	104	1.759	0.001	1.632	0.004	156	1.429	0.021	1.402	0.028
Beta	933	135	2.083	0.003	1.538	0.010	200	1.607	0.023	1.336	0.052
Delta	963	138	2.770	0.001	1.543	0.006	207	1.904	0.007	1.341	0.033
D614G	973	142	3.725	<.001	1.506	0.005	211	2.132	0.002	1.278	0.058
BA.4/BA.5	699	80	1.850	0.004	1.836	0.002	117	1.461	0.040	1.511	0.018
MDW	813	104	1.759	0.001	1.632	0.004	156	1.429	0.021	1.402	0.028

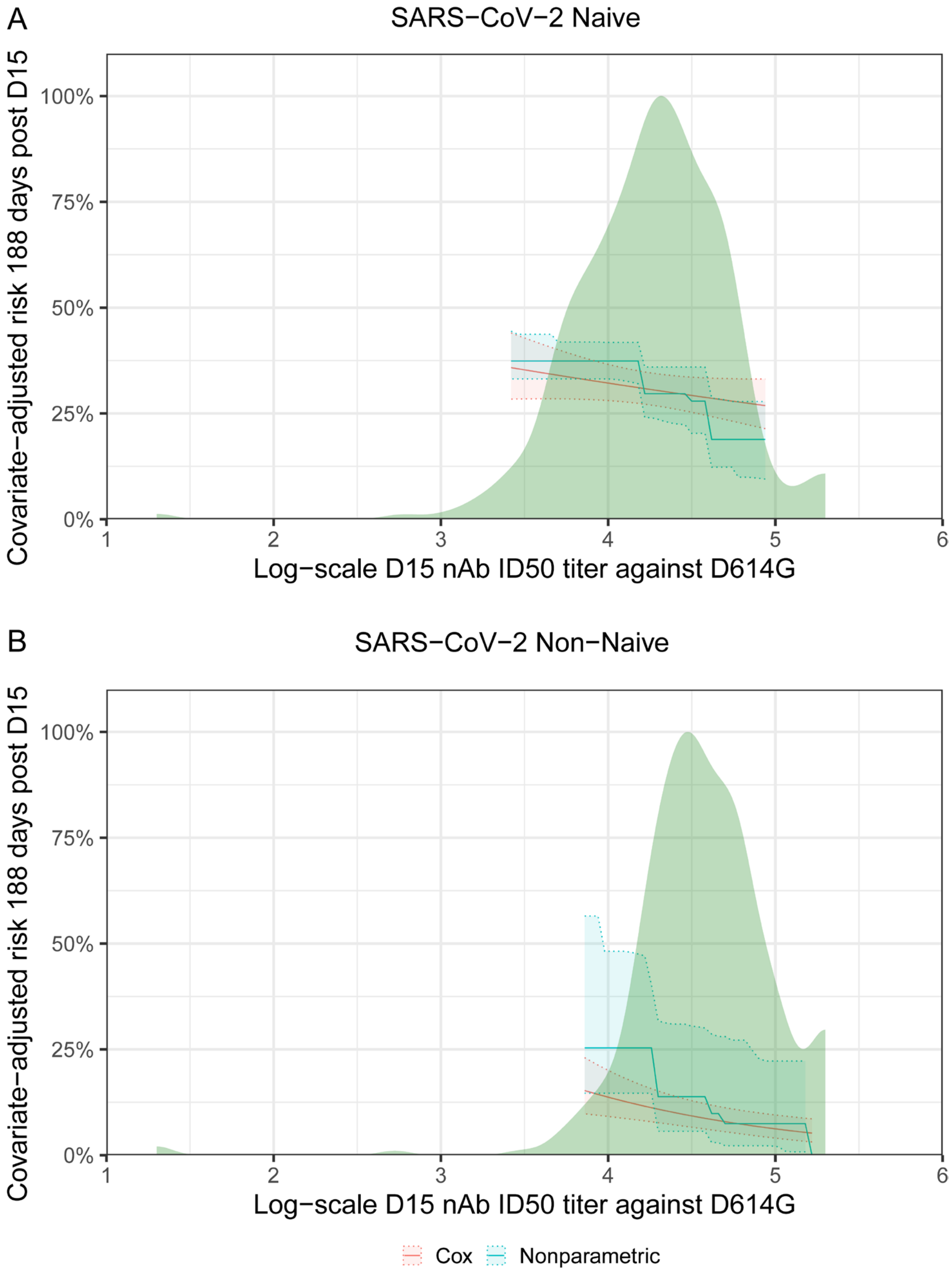

Supplementary Figure 11. Covariate-adjusted controlled risk of COVID-19 by D15 D614G titer, estimated using a Cox model (orange line) or a nonparametric method (turquoise line), in A) naïve participants or B) non-naïve participants, for COVID-19 endpoints from 7 to 188 days post D15. Both curves were restricted to the middle 95% of the marker distribution. Shaded regions represent 95% confidence intervals. The green shaded region is a smoothed histogram of log10 D15 D614G titer (AU/ml). Analyses adjusted for force of infection score and risk score. AU, arbitrary units; nAb-ID50, 50% inhibitory dilution neutralizing antibody titer. Source data are provided as a Source Data file.

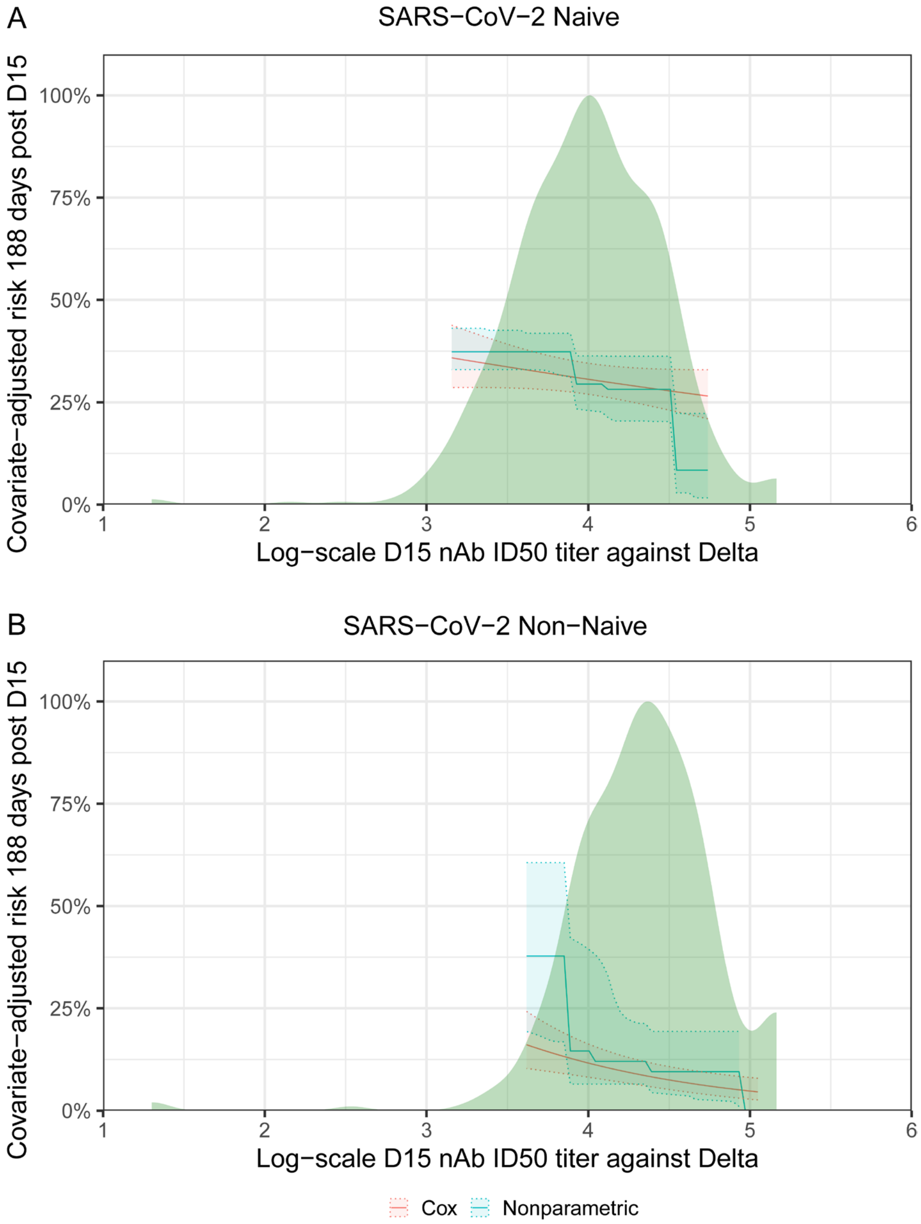

Supplementary Figure 12. Covariate-adjusted controlled risk of COVID-19 by D15 Delta titer, estimated using a Cox model (orange line) or a nonparametric method (turquoise line), in A) naïve participants or B) non-naïve participants, for COVID-19 endpoints from 7 to 188 days post D15. Both curves were restricted to the middle 95% of the marker distribution. Shaded regions represent 95% confidence intervals. The green shaded region is a smoothed histogram of log10 D15 Delta titer (AU/ml). Analyses adjusted for force of infection score and risk score. AU, arbitrary units; nAb-ID50, 50% inhibitory dilution neutralizing antibody titer. Source data are provided as a Source Data file.

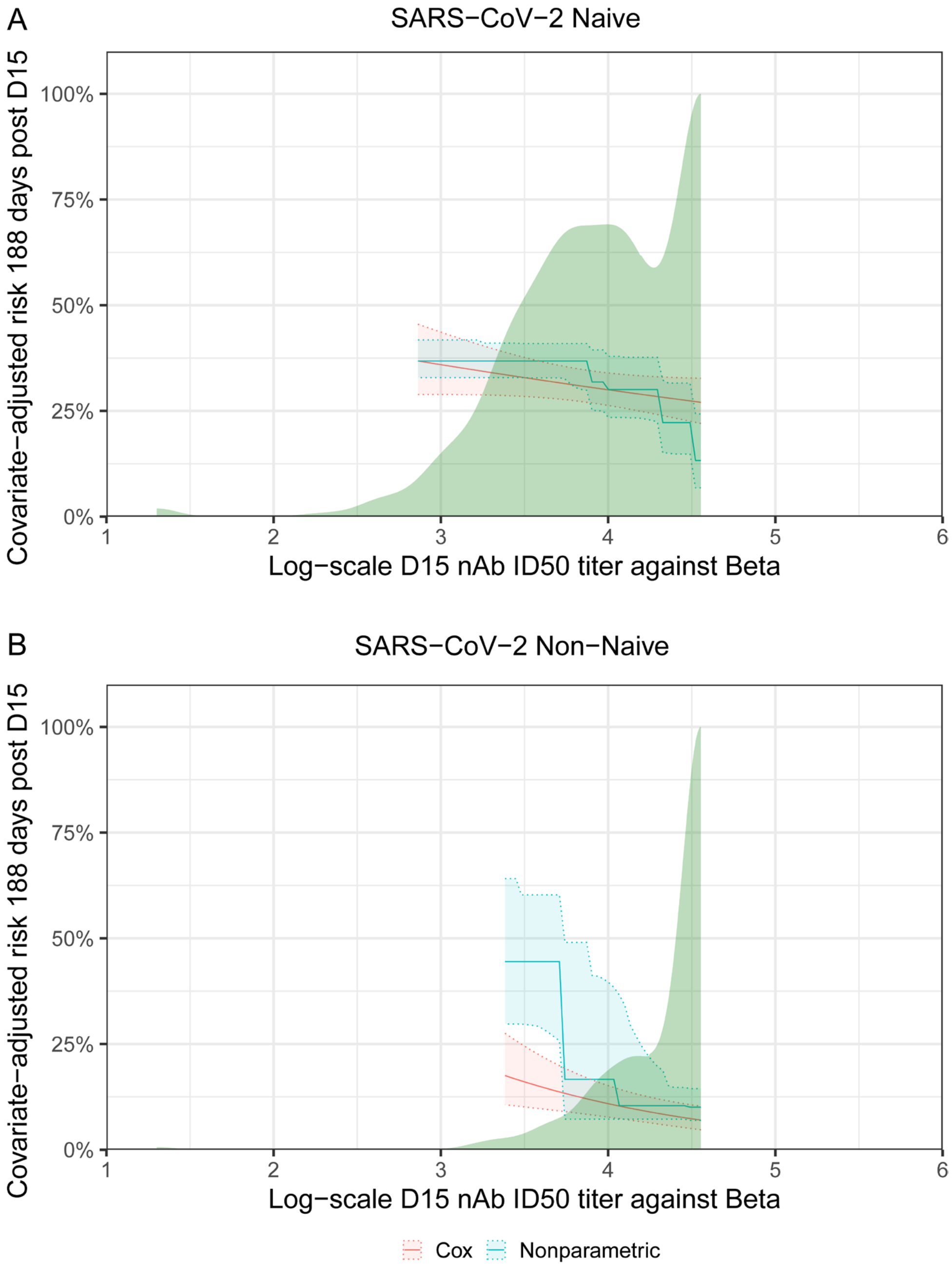

Supplementary Figure 13. Covariate-adjusted controlled risk of COVID-19 by D15 Beta titer, estimated using a Cox model (orange line) or a nonparametric method (turquoise line), in A) naïve participants or B) non-naïve participants, for COVID-19 endpoints 7 to 188 days post D15. Both curves were restricted to the middle 95% of the marker distribution. Shaded regions represent 95% confidence intervals. The green shaded region is a smoothed histogram of log10 D15 Beta titer (AU/ml). Analyses adjusted for force of infection score and risk score. AU, arbitrary units; nAb-ID50, 50% inhibitory dilution neutralizing antibody titer. Source data are provided as a Source Data file.

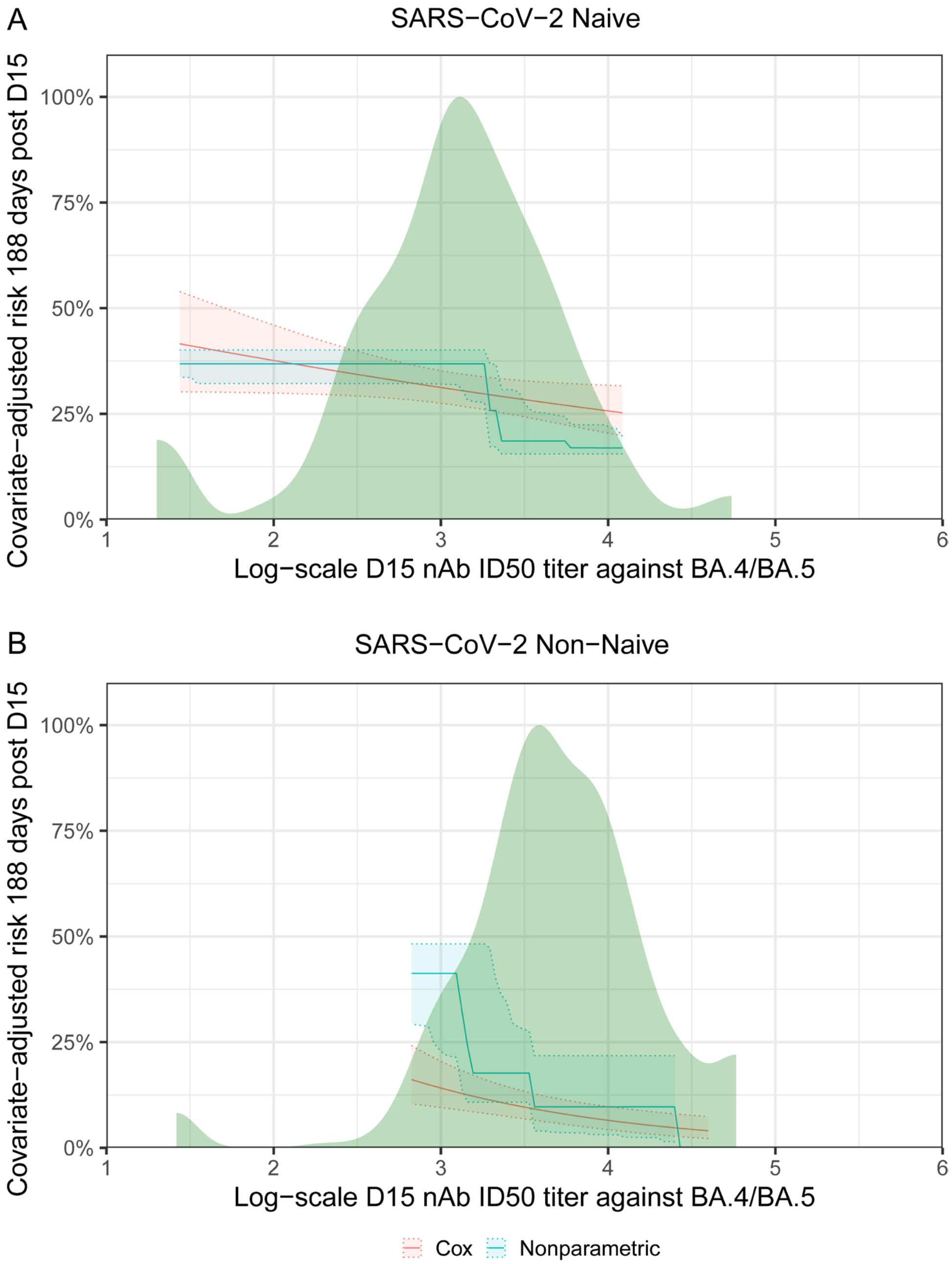

Supplementary Figure 14. Covariate-adjusted controlled risk of COVID-19 by D15 BA.4/BA.5 titer, estimated using a Cox model (orange line) or a nonparametric method (turquoise line), in A) SARS-CoV-2 naïve participants or B) SARS-CoV-2 non-naïve participants, for COVID-19 endpoints from 7 to 188 days post D15. Both curves were restricted to the middle 95% of the marker distribution. Shaded regions represent 95% confidence intervals. The green shaded region is a smoothed histogram of log10 D15 BA.4/BA.5 titer (AU/ml). Analyses adjusted for force of infection score and risk score. AU, arbitrary units; nAb-ID50, 50% inhibitory dilution neutralizing antibody titer. Source data are provided as a Source Data file.

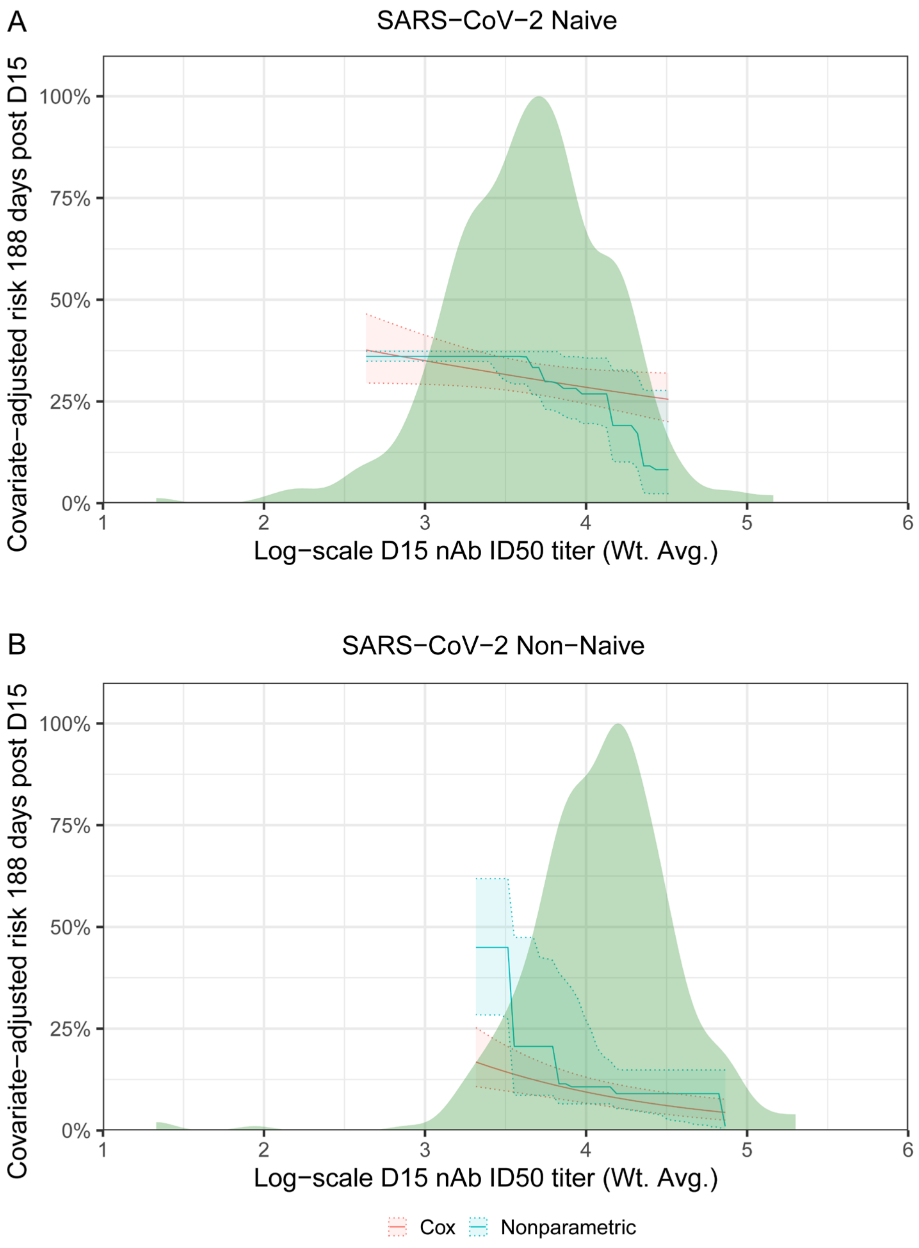

Supplementary Figure 15. Covariate-adjusted controlled risk of COVID-19 by D15 weighted average titer, estimated using a Cox model (orange line) or a nonparametric method (turquoise line), in A) naïve participants or B) non-naïve participants, for COVID-19 endpoints from 7 to 188 days post D15. Both curves were restricted to the middle 95% of the marker distribution. Shaded regions represent 95% confidence intervals. The green shaded region is a smoothed histogram of log10 D15 weighted average titer (AU/ml). Analyses adjusted for force of infection score and risk score. Wt. Avg. = Maximum diversity weighted geometric mean of the five nAb titers D614G, Beta, Delta, BA.1, and BA.4/BA.5. AU, arbitrary units; nAb-ID50, 50% inhibitory dilution neutralizing antibody titer. Source data are provided as a Source Data file.

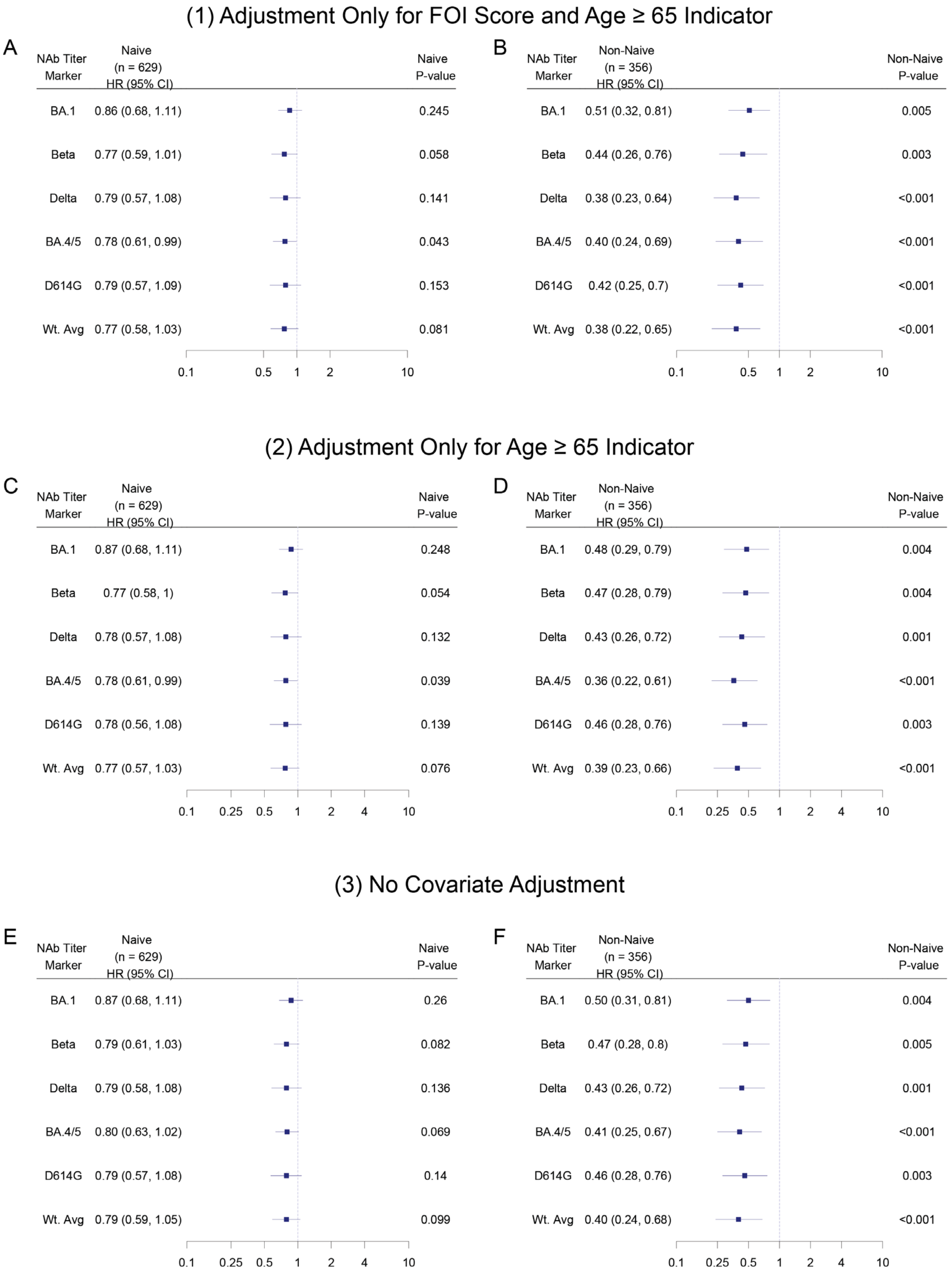

Supplementary Figure 16. Cox model covariate-adjusted hazard ratios of COVID-19 per 10-fold increase in D15 titer (AU/ml) for each marker BA.1, Beta, Delta, BA.4/BA.5, D614G, or weighted average (Wt. Avg.) for COVID-19 endpoints from 7 to 188 days post D15, with the following sensitivity analyses performed: 1) adjustment only for the FOI score and for the ≥ 65 age indicator; 2) adjustment only for the ≥ 65 age indicator; 3) no covariate adjustment.

Results are shown in A, C, E) naïve participants (for all panels A, C, E: BA.1 N=629, Beta N=629, Delta N=629, BA.4/BA.5 N=629, D614G N=629, Wt. Avg. N=629) or B, D, F) non-naïve participants (for all panels B, D, F: BA.1 N=356, Beta N=356, Delta N=356, BA.4/BA.5 N=356, D614G N=356, Wt. Avg. N=356). Point estimates, 95% confidence intervals (CIs), and 2-sided Wald p-values are shown. P-values were not adjusted for multiple comparisons. Wt. Avg. = Maximum diversity weighted geometric mean of the five titers D614G reference, Beta, Delta, Omicron BA.1, and Omicron BA.4/BA.5. AU, arbitrary units; FOI, force of infection; nAb, neutralizing antibody.

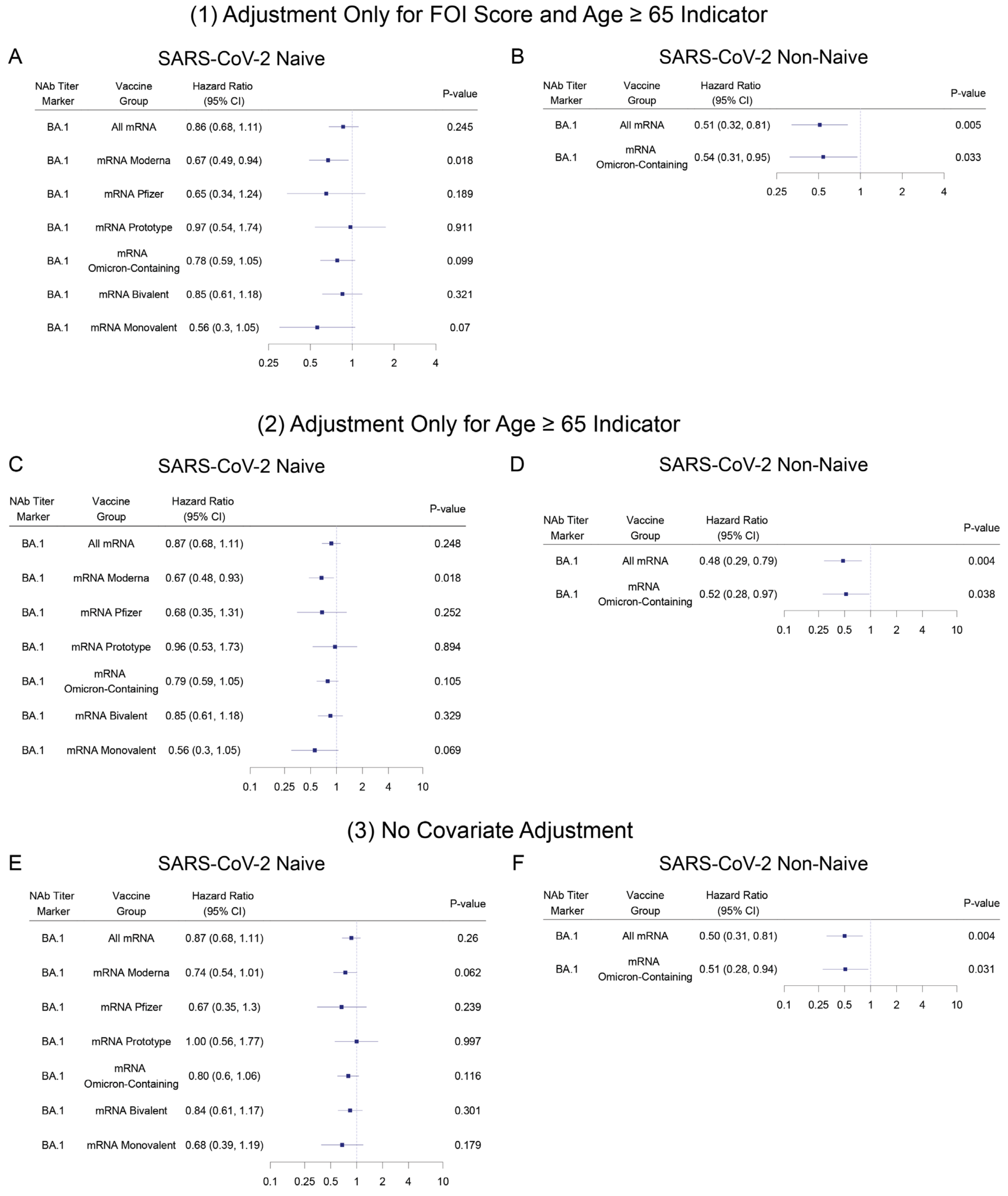

Supplementary Figure 17. Cox model covariate-adjusted hazard ratios of COVID-19 per 10-fold increase in D15 BA.1 titer (AU/ml), in A, C, E) naïve participants and in the designated subgroups (for all panels A, C, E: All mRNA N=629, mRNA Moderna N=306, mRNA Pfizer-BioNTech N=131, mRNA Prototype N=105, mRNA Omicron-containing N=459, mRNA Bivalent N=352, mRNA Monovalent N=107), or B, D, F) non-naïve participants and in the designated subgroup (for all panels B, D, F: All mRNA N=356, mRNA Omicron-containing

N=285) for COVID-19 endpoints from 7 to 188 days post D15, with the following sensitivity analyses performed: 1) adjustment only for the FOI score and for the ≥ 65 age indicator; 2) adjustment only for the ≥ 65 age indicator; 3) no covariate adjustment. Point estimates, 95% confidence intervals (CIs), and 2-sided Wald p-values are shown. P-values were not adjusted for multiple comparisons. Subgroup analyses were only conducted when the number of COVID-19 endpoints was equal to or exceeded 20, to ensure reasonable precision. AU, arbitrary units; FOI, force of infection; nAb, neutralizing antibody.

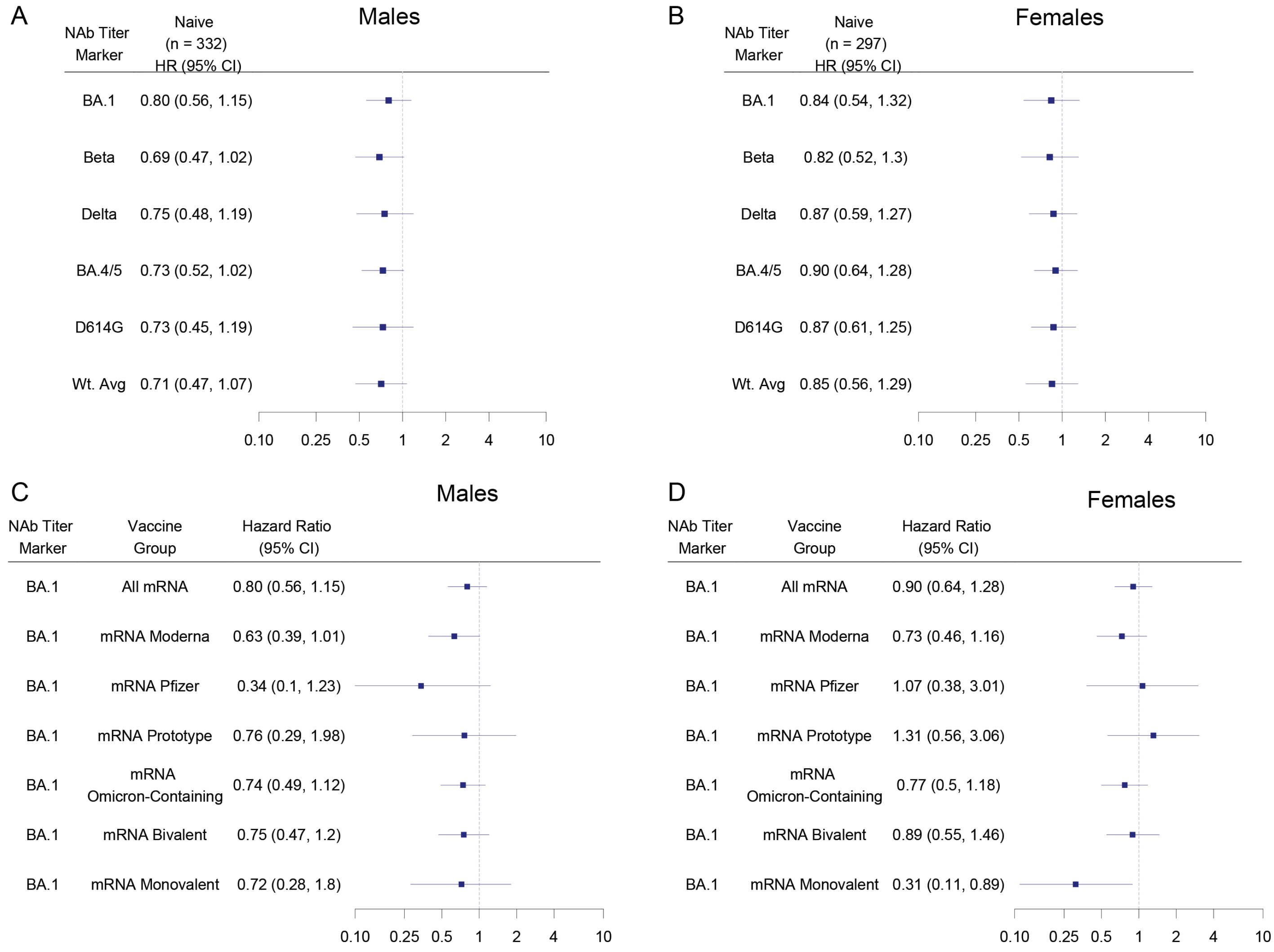

Supplementary Figure 18. A, B) Results of a post hoc analysis to estimate Cox model covariate-adjusted hazard ratios (HRs) of COVID-19 per 10-fold increase in D15 titer (AU/ml) for each marker BA.1, Beta, Delta, BA.4/BA.5, D614G, or weighted average (Wt. Avg.), in SARS-CoV-2 naïve participants, separately among (A) males and (B) females. C, D) Results of a post hoc analysis to estimate Cox model covariate-adjusted hazard ratios of COVID-19 per 10-fold increase in D15 BA.1 titer (AU/ml), in designated subgroups of SARS-CoV-2 naïve participants, separately among (C) males and (D) females. Follow-up was 7 to 188 days post D15. Analyses adjusted for force of infection score and risk score. Point estimates and 95% confidence intervals (CIs) are shown. Wt. Avg. = Maximum diversity weighted geometric mean of the five nAb titers D614G, Beta, Delta, Omicron BA.1, and Omicron BA.4/BA.5. AU, arbitrary units; nAb-ID50, 50% inhibitory dilution neutralizing antibody titer.

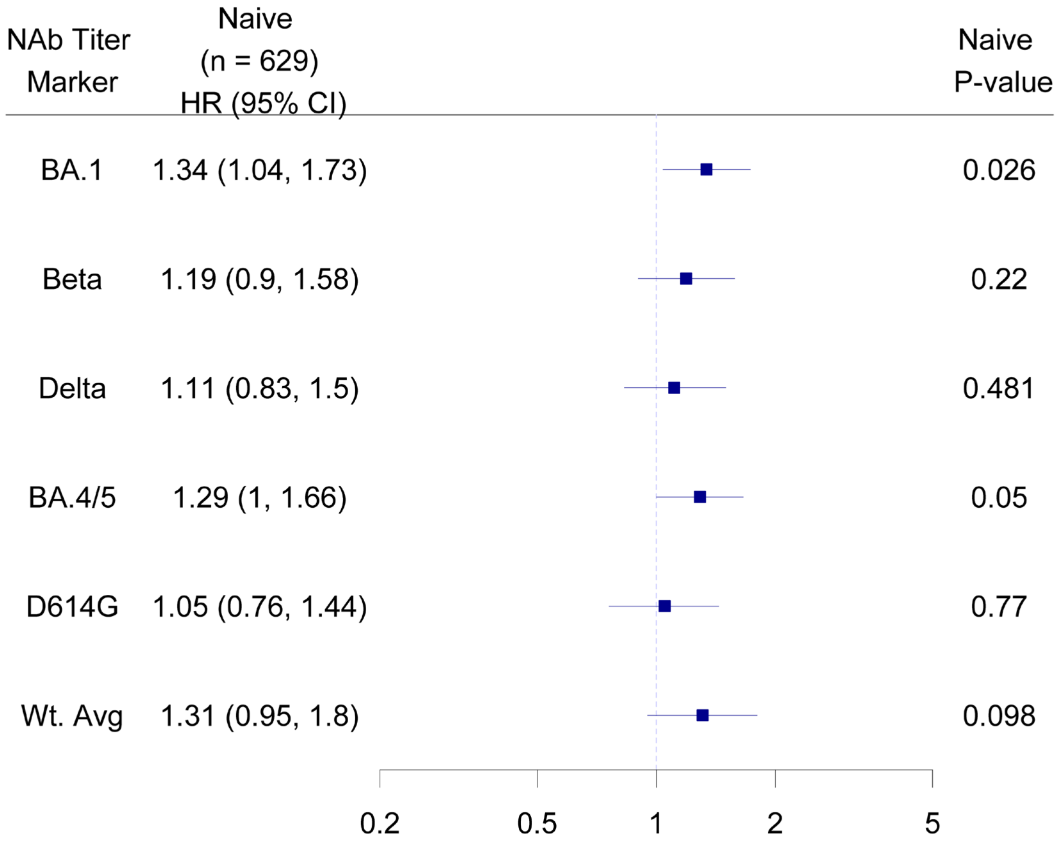

Supplementary Figure 19. Cox model covariate-adjusted hazard ratios of COVID-19 in naïve participants (N=629 for each row) per 10-fold increase in each of the six fold-rise titer markers, for COVID-19 endpoints from 7 to 188 days post D15. Point estimates, 95% confidence intervals (CIs), and 2-sided Wald p-values are shown. P-values were not adjusted for multiple comparisons. nAb, neutralizing antibody.

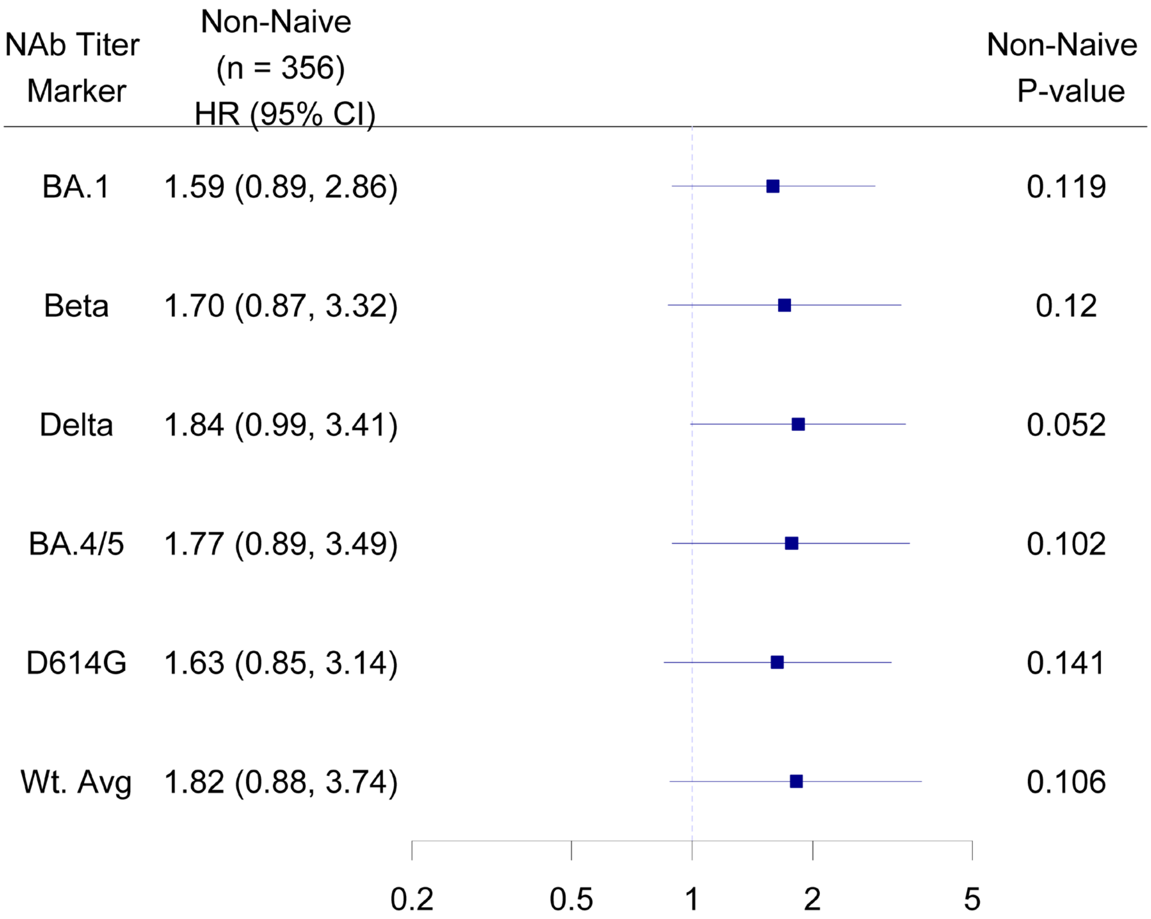

Supplementary Figure 20. Cox model covariate-adjusted hazard ratios of COVID-19 in non-naïve participants (N=356 for each row) per 10-fold increase in each of the six fold-rise titer markers, for COVID-19 endpoints from 7 to 188 days post D15. Point estimates, 95% confidence intervals (CIs), and 2-sided Wald p-values are shown. P-values were not adjusted for multiple comparisons. nAb, neutralizing antibody.

NAb Titer Marker	Vaccine Group	Hazard Ratio (95% CI)	P-value
BA.1	All mRNA	1.34 (1.04, 1.73)	0.026
BA.1	mRNA Moderna	1.21 (0.84, 1.74)	0.301
BA.1	mRNA Pfizer	1.28 (0.68, 2.39)	0.439
BA.1	mRNA Prototype	1.59 (0.85, 2.98)	0.144
BA.1	mRNA Omicron-Containing	1.36 (1.01, 1.84)	0.043
BA.1	mRNA Bivalent	1.42 (1.02, 1.97)	0.036
BA.1	mRNA Monovalent	1.23 (0.59, 2.53)	0.58

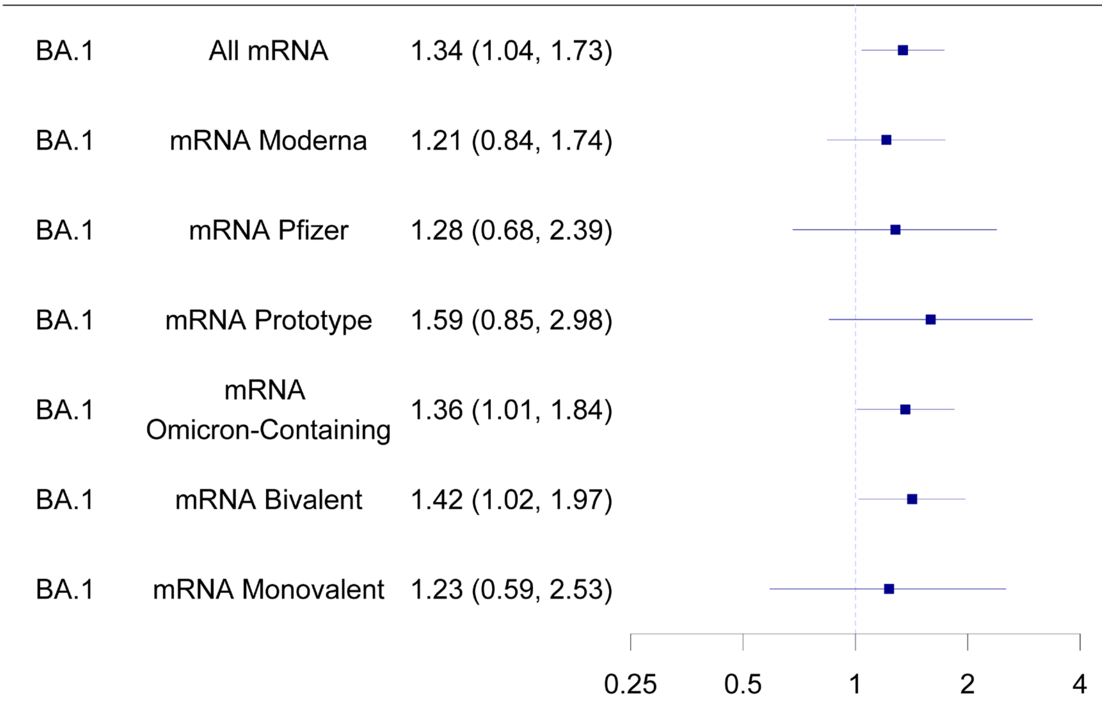

Supplementary Figure 21. Cox model covariate-adjusted hazard ratios of COVID-19 in naïve participants and in the designated subgroups (All mRNA N=629, mRNA Moderna N=306, mRNA Pfizer-BioNTech N=131, mRNA Prototype N=105, mRNA Omicron-containing N=459, mRNA Bivalent N=352, mRNA Monovalent N=107), per 10-fold increase in BA.1 titer fold-rise from D1 to D15, for COVID-19 endpoints from 7 to 188 days post D15. Point estimates, 95% confidence intervals (CIs), and 2-sided Wald p-values are shown. P-values were not adjusted for multiple comparisons. Subgroup analyses were only conducted when the number of endpoints exceeded 20, to ensure reasonable precision. nAb, neutralizing antibody.

NAb Titer Marker	Vaccine Group	Hazard Ratio (95% CI)	P-value
BA.1	All mRNA	1.59 (0.89, 2.86)	0.119
BA.1	mRNA Omicron-Containing	1.77 (0.91, 3.41)	0.09

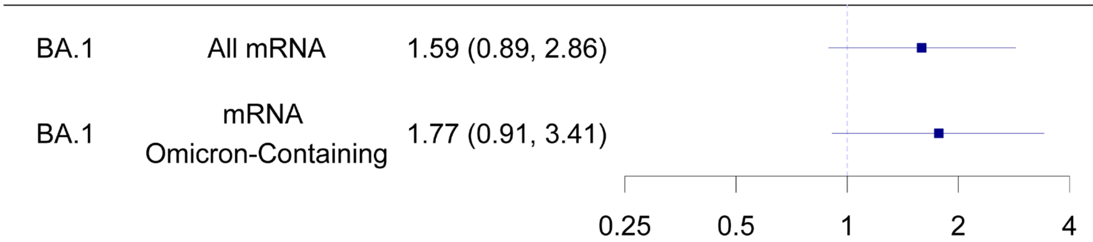

Supplementary Figure 22. Cox model covariate-adjusted hazard ratios of COVID-19 in non-naïve participants and in the designated subgroup (all mRNA N=356, mRNA Omicron-containing N=285), per 10-fold increase in BA.1 titer fold-rise from D1 to D15, for COVID-19 endpoints from 7 to 188 days post D15. Point estimates, 95% confidence intervals (CIs), and 2-sided Wald p-values are shown. P-values were not adjusted for multiple comparisons. Subgroup analyses were only conducted when the number of endpoints exceeded 20, to ensure reasonable precision. nAb, neutralizing antibody.

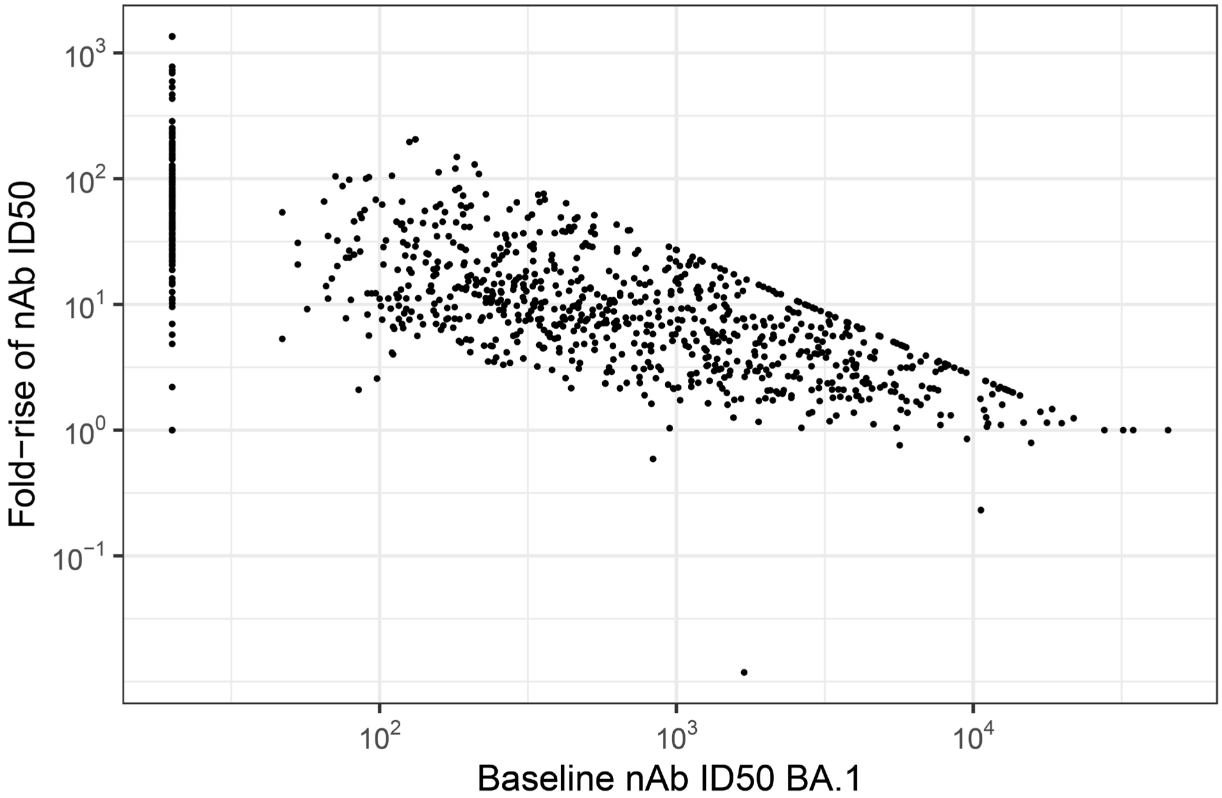

Supplementary Figure 23. Scatterplot of baseline (D1) BA.1 ID50 nAb titer and fold-rise (D15/D1) in BA.1 ID50 nAb titer for participants in the per-protocol correlates cohort. nAb ID50, 50% inhibitory dilution neutralizing antibody.

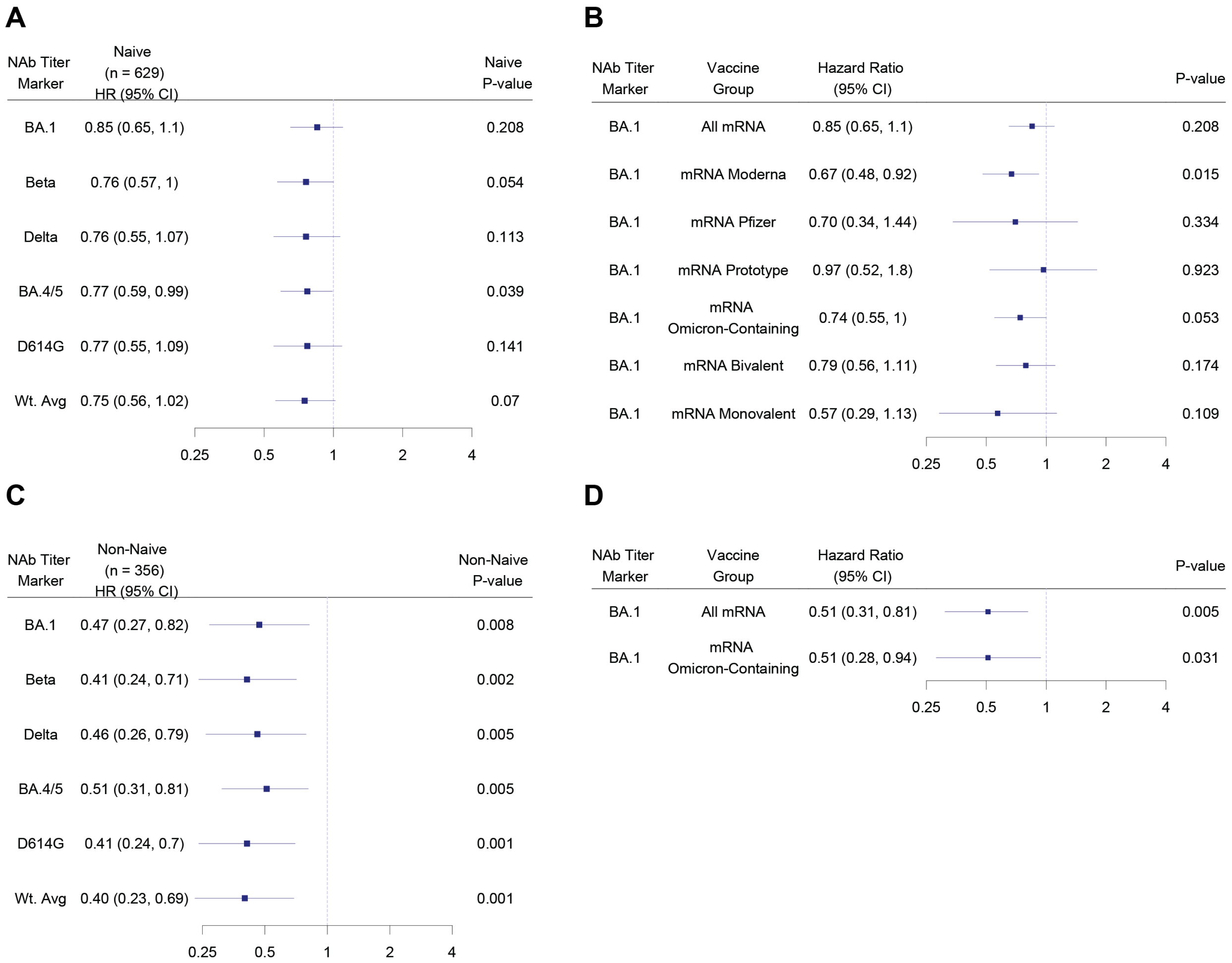

Supplementary Figure 24. Cox model covariate-adjusted hazard ratios of COVE COVID-19 (definition below) per 10-fold increase in D15 nAb-ID50 titer, shown separately in SARS-CoV-2 (A, B) naïve and (C, D) non-naïve participants. (A) and (C) show hazard ratios per 10-fold increase in D15 titer (AU/ml) for each marker BA.1, Beta, Delta, BA.4/BA.5, D614G, or weighted average (Wt. Avg.) (A: N=629 participants for each row; C: N=356 participants for each row). (B) and (D) show hazard ratios per 10-fold increase in D15 BA.1 titer (AU/ml) in the designated subgroups (B: All mRNA N=629, mRNA Moderna N=306, mRNA Pfizer-BioNTech N=131, mRNA Prototype N=105, mRNA Omicron-containing N=459, mRNA Bivalent N=352, mRNA Monovalent N=107; D: all mRNA N=356, mRNA Omicron-containing N=285). Subgroup analyses were only conducted when the number of COVE COVID-19 endpoints was equal to or exceeded 20. Analyses adjusted for force of infection score and risk score. Point estimates, 95% confidence intervals (CIs), and 2-sided Wald p-values are shown. P-values were not adjusted for multiple comparisons. Wt. Avg. = Maximum diversity weighted geometric mean of the five nAb titers D614G reference, Beta, Delta, Omicron BA.1, and Omicron BA.4/BA.5. AU, arbitrary units; nAb-ID50, 50% inhibitory dilution neutralizing antibody titer.

Definition of the COVE COVID-19 endpoint:

- Self-reported positive SARS-CoV-2 test (RT-PCR or antigen test) or study-conducted positive SARS-CoV-2 test (nasal swab and subsequent nucleic acid amplification test at an unscheduled illness visit) AND
- At least TWO of the following systemic symptoms: fever (≥38°C), chills, myalgia, headache, sore throat, new loss of taste or smell, OR
- At least ONE of the following respiratory signs/symptoms: cough, shortness of breath or difficulty breathing, OR clinical or radiographical evidence of pneumonia.

Bullets (2) and (3) above are the clinical criteria used for a COVID-19 case definition in the primary COVE efficacy analyses.[4,5]

162/181 (89.5%) of COVID-19 endpoints in naïve participants satisfied the COVE clinical criteria and supportive laboratory criteria for a COVE endpoint.
29/32 (90.6%) of COVID-19 endpoints in non-naïve participants satisfied the COVE clinical criteria and supportive laboratory criteria for a COVE endpoint.

Supplementary Table 10. Estimated half-lives (days), calculated using a linear mixed effects model, and their associated 95% confidence intervals for BA.1 titer in each vaccine arm among naïve and non-naïve participants.

Arm	Naïve	Non-naive
1. Moderna Prototype	51.0 (47.1 to 55.6)	61.4 (53.0 to 73.1)
2. Moderna Beta + Omicron	57.9 (52.2 to 65.0)	70.0 (57.8 to 88.6)
4. Moderna Delta + Omicron	56.8 (52.4 to 62.1)	62.7 (52.62 to 77.6)
5. Moderna Omicron	52.8 (48.5 to 58.0)	79.2 (66.2 to 98.5)
6. Moderna Omicron + Prototype	65.4 (58.2 to 74.7)	86.0 (66.6 to 121.3)
7. Pfizer-BioNTech Prototype	55.8 (47.9 to 66.6)	71.7 (57.9 to 94.1)
8. Pfizer-BioNTech Beta + Omicron	71.7 (57.9 to 94.1)	65.4 (52.8 to 86.0)
9. Pfizer-BioNTech Omicron	73.4 (63.2 to 87.7) (	88.5 (72.3 to 114.2)
10. Pfizer-BioNTech Beta	66.9 (56.6 to 81.9)	75.3 (61.7 to 96.6)
11. Pfizer-BioNTech Beta + Prototype	70.0 (60.6 to 82.9)	77.2 (64.0 to 97.2)
12. Pfizer-BioNTech Omicron + Prototype	62.7 (52.6 to 77.6)	97.1 (72.4 to 147.5)
16. Pfizer-BioNTech Omicron BA.1 + Prototype	143.4 (108.1 to 212.9)	143.4 (124.7 to 168.5)
17. Pfizer-BioNTech Omicron BA.4/BA.5+ Prototype	107.5 (85.9 to 143.7)	136.8 (117.9 to 163.0)

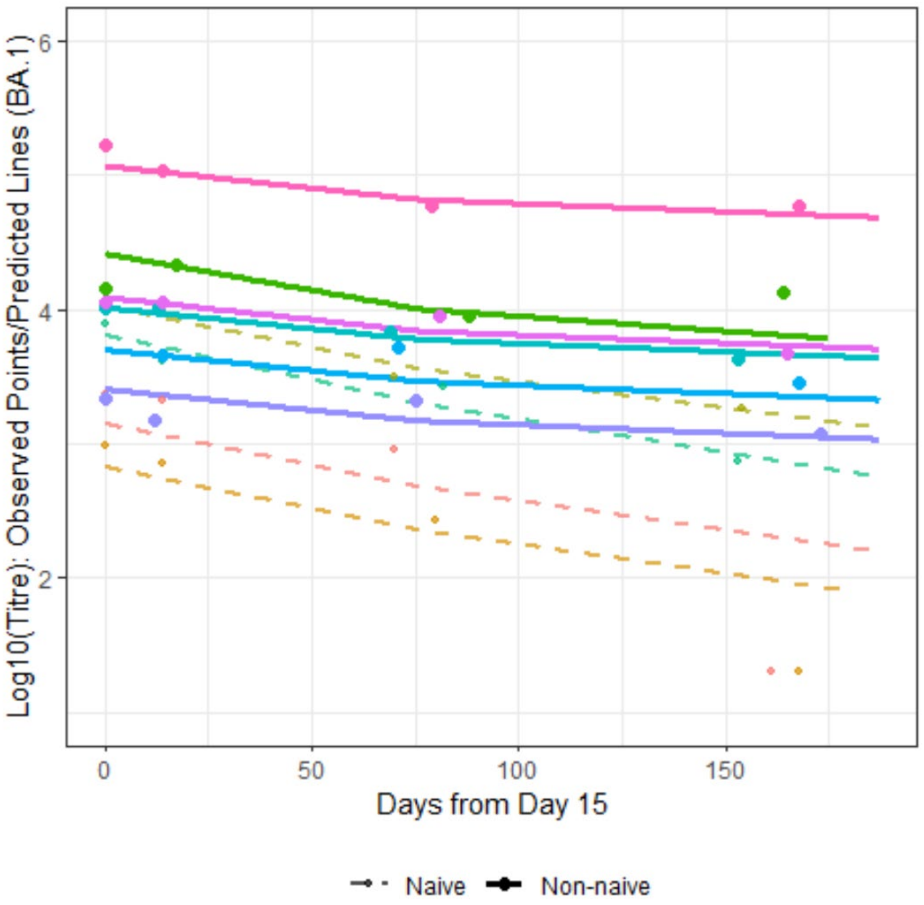

Supplementary Figure 25. Empirically observed BA.1 log10 titers at D1, D29, D91, and D181 (filled dots) for 10 randomly selected non-case participants along with their predicted BA.1 log10 titers titer over time based on a biphasic decay model (dashed lines for naïve and solid for non-naïve participants).

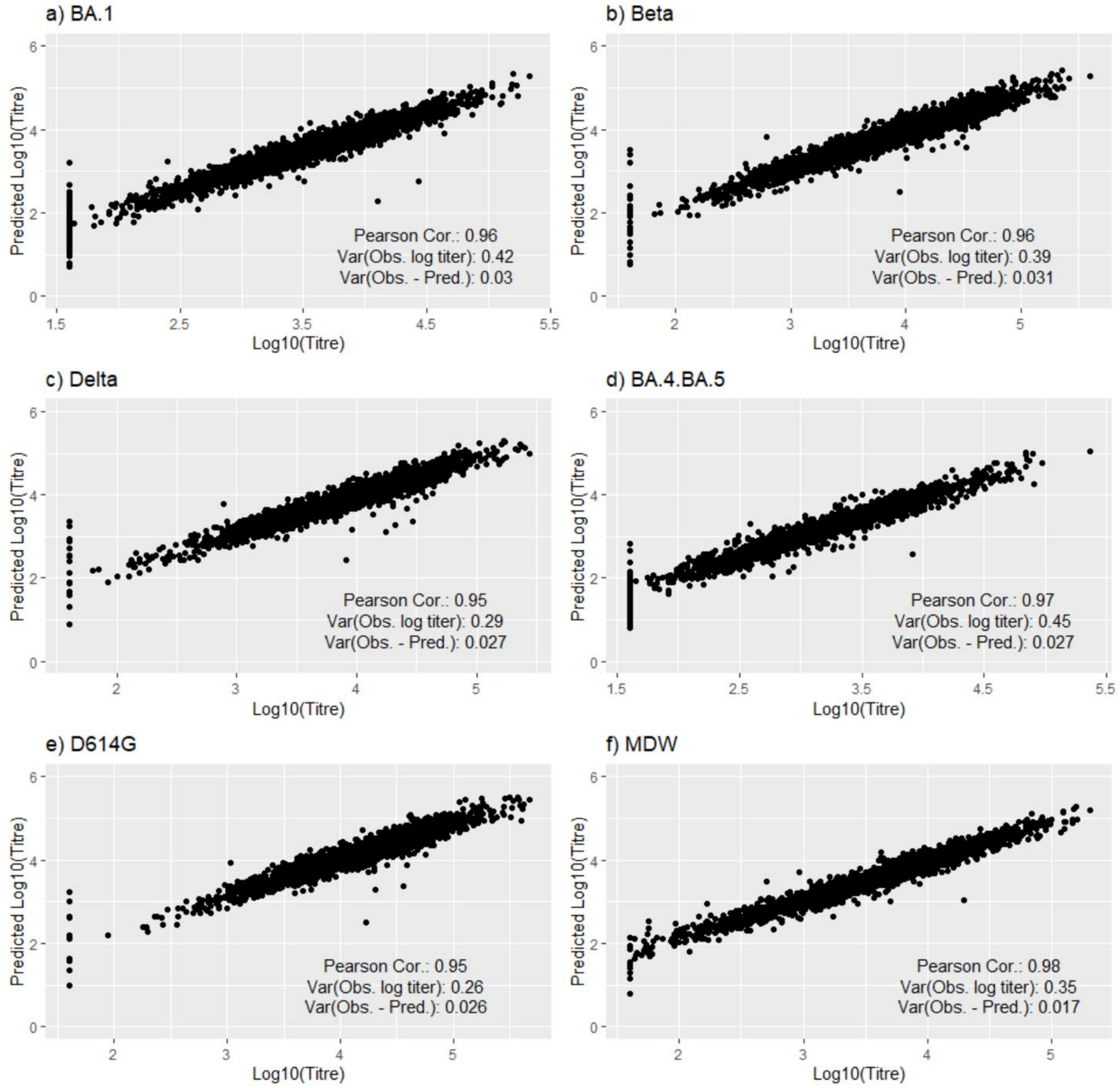

Supplementary Figure 26. Measured versus predicted (based on a biphasic antibody decay model) log10 titers at D15, D29, D91, and D181 along with summary statistics. Titers are shown for: A) Omicron BA.1, B) Beta, C) Delta, D) Omicron BA.4/BA.5, E) D614G, and F) MDW.

MDW = Maximum diversity weighted geometric mean of the five nAb titers D614G, Beta, Delta, Omicron BA.1, and Omicron BA.4/BA.5. Pearson Cor.: Pearson Correlation. Var(Obs. log titer): variance of the observed log10 titer. Var(Obs. - Pred.): Variance of the difference between the observed log10 titer and the predicted log10 titer.

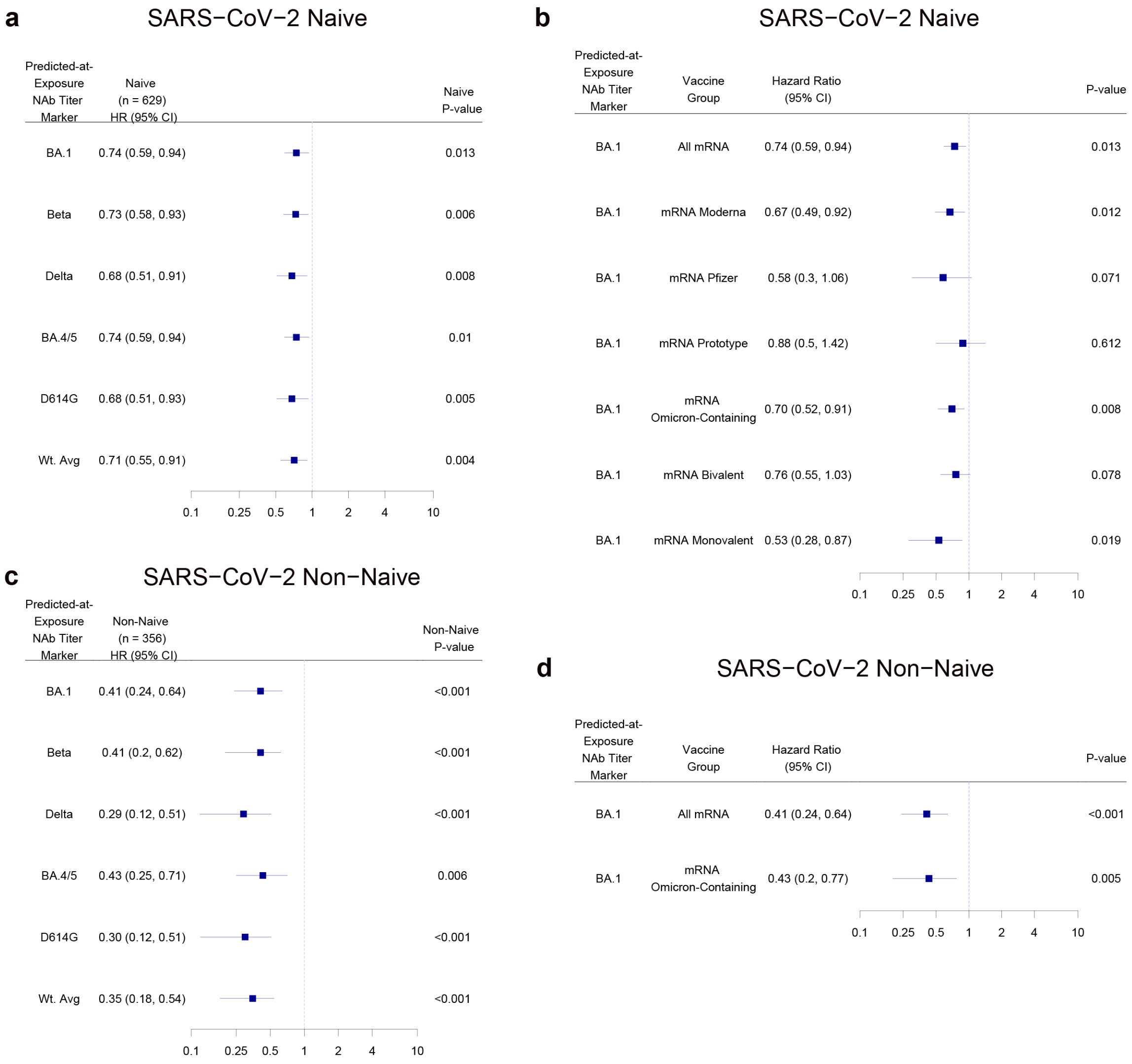

Supplementary Figure 27. A, C) Calendar-time-based Cox model hazard ratios of COVID-19 per 10-fold increase in predicted-at-exposure titer (AU/ml) for each marker BA.1, Beta, Delta, BA.4/BA.5, D614G, or weighted average (Wt. Avg.) for COVID-19 endpoints from 7 to 188 days post D15, in A) naïve and C) non-naïve participants. B, D) Calendar-time-based Cox model hazard ratios of COVID-19 per 10-fold increase in predicted-at-exposure BA.1 titer (AU/ml) in the designated subgroups in B) naïve and D) non-naïve participants. Numbers of participants used in the analyses: A) BA.1 N=629, Beta N=629, Delta N=629, BA.4/BA.5 N=605, D614G N=629, Wt. Avg. N=629; B) All mRNA N=629, mRNA Moderna N=306, mRNA Pfizer-BioNTech N=131, mRNA Prototype N=105, mRNA Omicron-containing N=459, mRNA Bivalent N=352, mRNA Monovalent N=107; C) BA.1 N=356, Beta N=356, Delta N=356, BA.4/BA.5 N=351, D614G N=356, Wt. Avg. N=356; D) All mRNA N=356, mRNA Omicron-containing N=285. Subgroup analyses were only conducted when the number of endpoints was equal to or exceeded 20. Point estimates, 95% percentile bootstrap confidence intervals (CIs), and 2-sided bootstrap p-values are shown. P-values were not adjusted for multiple comparisons. AU, arbitrary units; nAb-ID50, 50% inhibitory dilution neutralizing antibody titer. No covariate adjustment was done for any of the plots.

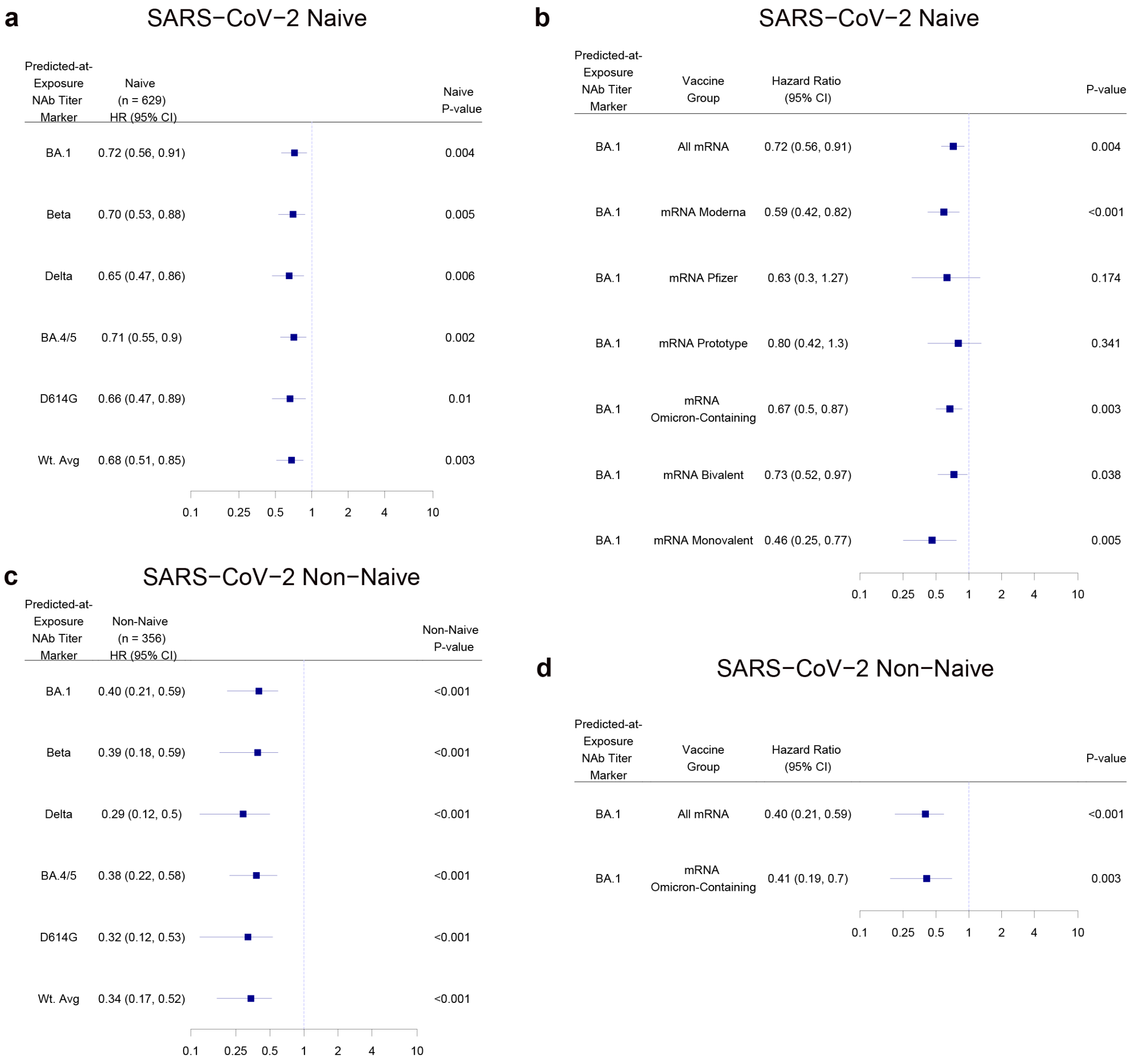

Supplementary Figure 28. Cox model covariate-adjusted hazard ratios of COVE COVID-19 (definition below) per 10-fold increase in predicted-at-exposure nAb-ID50 titer, shown separately in SARS-CoV-2 (A, B) naïve and (C, D) non-naïve participants. (A) and (C) show hazard ratios per 10-fold increase in predicted-at-exposure titer (AU/ml) for each marker BA.1, Beta, Delta, BA.4/BA.5, D614G, or weighted average (Wt. Avg.). (B) and (D) show hazard ratios per 10-fold increase in predicted-at-exposure BA.1 titer (AU/ml) in the designated subgroups. Numbers of participants used in the analyses: A) BA.1 N=629, Beta N=629, Delta N=629, BA.4/BA.5 N=605, D614G N=629, Wt. Avg. N=629; B) All mRNA N=629, mRNA Moderna N=306, mRNA Pfizer-BioNTech N=131, mRNA Prototype N=105, mRNA Omicron-containing N=459, mRNA Bivalent N=352, mRNA Monovalent N=107; C) BA.1 N=356, Beta N=356, Delta N=356, BA.4/BA.5 N=351, D614G N=356, Wt. Avg. N=356; D) All mRNA N=356, mRNA Omicron-containing N=285. Subgroup analyses were only conducted when the number of COVE COVID-19 endpoints was equal to or exceeded 20. Analyses adjusted for risk score. Point estimates, 95% bootstrap percentile confidence

intervals (CIs), and 2-sided bootstrap p-values are shown. P-values were not adjusted for multiple comparisons. Wt. Avg. = Maximum diversity weighted geometric mean of the five nAb titers D614G reference, Beta, Delta, Omicron BA.1, and Omicron BA.4/BA.5. AU, arbitrary units; nAb-ID50, 50% inhibitory dilution neutralizing antibody titer.

Definition of the COVE COVID-19 endpoint:

- Self-reported positive SARS-CoV-2 test (RT-PCR or antigen test) or study-conducted positive SARS-CoV-2 test (nasal swab and subsequent nucleic acid amplification test at an unscheduled illness visit) AND
- At least TWO of the following systemic symptoms: fever (≥38°C), chills, myalgia, headache, sore throat, new loss of taste or smell, OR
- At least ONE of the following respiratory signs/symptoms: cough, shortness of breath or difficulty breathing, OR clinical or radiographical evidence of pneumonia.

Bullets (2) and (3) above are the clinical criteria used for a COVID-19 case definition in the primary COVE efficacy analyses.{Baden, 2021 #2;El Sahly, 2021 #73}

162/181 (89.5%) of COVID-19 endpoints in naïve participants satisfied the COVE clinical criteria and supportive laboratory criteria for a COVE endpoint.
29/32 (90.6%) of COVID-19 endpoints in non-naïve participants satisfied the COVE clinical criteria and supportive laboratory criteria for a COVE endpoint.

Supplementary Table 11. Event time and lineage, and whether the lineage was obtained by sequencing (Observed = TRUE) or imputed (Observed = FALSE), for COVID-19 study endpoints included in the analysis (from 7 to 188 days post D15) in the Moderna Beta + Omicron study arm.

	Event Time	Lineage	Observed
1	104	BA.5	TRUE
2	152	BA.4	TRUE
3	155	BA.5	TRUE
4	18	BA.2	TRUE
5	92	BA.2	TRUE
6	125	BA.5	TRUE
7	75	BA.5	TRUE
8	70	BA.5	TRUE
9	151	BA.5	TRUE
10	146	BA.5	TRUE
11	40	BA.2	FALSE
12	56	BA.2	FALSE
13	115	BA.5	TRUE
14	29	BA.2	TRUE
15	27	BA.2	TRUE
16	52	BA.5	TRUE
17	61	BA.2	FALSE
18	171	BA.5	TRUE
19	115	BA.4	TRUE
20	162	BA.5	TRUE
21	58	BA.2	TRUE
22	29	BA.2	FALSE
23	94	BA.5	FALSE
24	65	BA.5	TRUE
25	102	BA.5	FALSE
26	29	BA.2	TRUE
27	40	BA.2	TRUE
28	64	BA.2	TRUE
29	36	BA.4	TRUE
30	188	BA.5	FALSE
31	88	BA.5	TRUE
32	38	BA.2	FALSE
33	58	BA.5	TRUE
34	19	BA.2	TRUE
35	67	BA.5	FALSE
36	8	BA.2	TRUE
37	10	BA.2	TRUE

Supplementary Table 12. Event time and lineage, and whether the lineage was obtained by sequencing (Observed = TRUE) or imputed (Observed = FALSE), for COVID-19 study endpoints included in the analysis (from 7 to 188 days post D15) in the Moderna Delta + Omicron study arm.

	Event Time	Lineage	Observed
1	160	BA.5	TRUE
2	79	BA.5	FALSE
3	137	BA.5	TRUE
4	22	XZ	TRUE
5	45	BA.2	FALSE
6	52	BA.2	TRUE
7	72	BA.5	TRUE
8	56	BA.5	TRUE
9	129	BA.5	FALSE
10	136	BA.5	TRUE
11	31	BA.2	TRUE
12	111	BA.4	TRUE
13	17	BA.2	TRUE
14	143	BA.5	FALSE
15	10	BA.4	TRUE
16	26	BA.2	TRUE
17	46	BA.5	TRUE
18	16	BA.2	TRUE
19	75	BA.5	TRUE
20	24	BA.2	TRUE
21	76	BA.5	TRUE
22	25	BA.2	TRUE
23	124	BA.4	TRUE
24	20	BA.2	TRUE
25	60	BA.5	TRUE
26	50	BA.5	TRUE
27	134	BA.5	FALSE
28	13	BA.4	TRUE

Supplementary Table 13. Event time and lineage, and whether the lineage was obtained by sequencing (Observed = TRUE) or imputed (Observed = FALSE), for COVID-19 study endpoints included in the analysis (from 7 to 188 days post D15) in the Moderna Omicron study arm.

	EventTime	Lineage	Observed
1	24	BA.2	FALSE
2	33	BA.2	TRUE
3	148	BA.5	TRUE
4	23	BA.2	TRUE
5	167	BA.5	TRUE
6	144	BA.5	TRUE
7	132	BA.5	FALSE
8	97	BA.5	TRUE
9	96	BA.5	TRUE
10	60	BA.5	TRUE
11	35	BA.2	FALSE
12	64	BA.2	FALSE
13	166	BA.5	TRUE
14	112	BA.5	TRUE
15	65	BA.5	TRUE
16	162	BA.5	TRUE
17	156	BA.5	TRUE
18	115	BA.5	TRUE
19	118	BA.5	FALSE
20	47	BA.5	TRUE
21	131	BA.5	TRUE
22	61	BA.4	TRUE
23	76	BA.5	FALSE
24	54	BA.5	FALSE
25	152	BA.5	TRUE

Supplementary Table 14. Event time and lineage, and whether the lineage was obtained by sequencing (Observed = TRUE) or imputed (Observed = FALSE), for COVID-19 study endpoints included in the analysis (from 7 to 188 days post D15) in the Moderna Omicron + Prototype study arm.

	Event Time	Lineage	Observed
1	15	BA.2	TRUE
2	43	BA.2	TRUE
3	74	BA.5	TRUE
4	53	BA.2	TRUE
5	69	BA.4	TRUE
6	35	BA.2	FALSE
7	129	BA.5	TRUE
8	96	BA.4	TRUE
9	93	BA.5	TRUE
10	103	BA.5	FALSE
11	62	BA.5	TRUE
12	31	BA.2	TRUE
13	73	BA.5	FALSE
14	154	BA.5	TRUE
15	30	BA.2	TRUE
16	103	BA.5	FALSE
17	150	BA.5	FALSE
18	136	BA.5	TRUE
19	176	BA.5	TRUE
20	32	BA.2	FALSE
21	68	BA.5	TRUE
22	55	BA.5	TRUE
23	29	BA.2	FALSE
24	94	BA.5	TRUE
25	17	BA.5	TRUE
26	12	BA.2	FALSE

Supplementary Table 15. Event time and lineage, and whether the lineage was obtained by sequencing (Observed = TRUE) or imputed (Observed = FALSE), for COVID-19 study endpoints included in the analysis (from 7 to 188 days post D15) in the Moderna Prototype study arm.

	EventTime	Lineage	Observed
1	82	BA.5	TRUE
2	64	BA.5	TRUE
3	36	BA.2	TRUE
4	42	BA.2	FALSE
5	80	BA.5	FALSE
6	106	BA.5	FALSE
7	154	BA.4	TRUE
8	42	BA.2	TRUE
9	40	BA.2	TRUE
10	60	BA.5	TRUE
11	25	BA.2	TRUE
12	55	BA.2	TRUE
13	123	BA.5	TRUE
14	94	BA.5	FALSE
15	102	BA.5	TRUE
16	66	BA.5	TRUE
17	35	BA.2	TRUE
18	104	BA.5	TRUE
19	90	BA.5	FALSE
20	73	BA.4	TRUE
21	28	BA.2	TRUE
22	57	BA.5	TRUE
23	35	BA.2	FALSE
24	54	BA.5	TRUE
25	127	BA.5	TRUE
26	188	BA.5	FALSE
27	163	BA.5	TRUE

Supplementary Table 16. Event time and lineage, and whether the lineage was obtained by sequencing (Observed = TRUE) or imputed (Observed = FALSE), for COVID-19 study endpoints included in the analysis (from 7 to 188 days post D15) in the Pfizer-BioNTech Stage-2 Beta study arm.

	Event Time	Lineage	Observed
1	82	BA.5	TRUE
2	112	BA.5	TRUE
3	89	BA.5	FALSE
4	23	BA.4	TRUE
5	14	BA.4	TRUE
6	116	BA.5	FALSE
7	64	BA.5	TRUE
8	17	BA.2	FALSE
9	188	BA.5	FALSE

Supplementary Table 17. Event time and lineage, and whether the lineage was obtained by sequencing (Observed = TRUE) or imputed (Observed = FALSE), for COVID-19 study endpoints included in the analysis (from 7 to 188 days post D15) in the Pfizer-BioNTech Stage-2 Beta + Omicron study arm.

	Event Time	Lineage	Observed
1	177	BA.5	FALSE
2	43	BA.5	TRUE
3	70	BA.5	FALSE
4	17	BA.2	FALSE
5	72	BA.5	TRUE
6	9	BA.4	TRUE
7	66	BA.5	TRUE

Supplementary Table 18. Event time and lineage, and whether the lineage was obtained by sequencing (Observed = TRUE) or imputed (Observed = FALSE), for COVID-19 study endpoints included in the analysis (from 7 to 188 days post D15) in the Pfizer-BioNTech Stage-2 Beta + Prototype study arm.

	Event Time	Lineage	Observed
1	26	BA.5	TRUE
2	48	BA.5	TRUE
3	38	BA.5	FALSE
4	24	BA.5	TRUE
5	73	BA.2	TRUE
6	14	BA.4	TRUE
7	73	BA.5	TRUE
8	103	BA.5	FALSE
9	108	BA.5	TRUE

Supplementary Table 19. Event time and lineage, and whether the lineage was obtained by sequencing (Observed = TRUE) or imputed (Observed = FALSE), for COVID-19 study endpoints included in the analysis (from 7 to 188 days post D15) in the Pfizer-BioNTech Stage-2 Omicron study arm.

	EventTime	Lineage	Observed
1	43	BA.5	FALSE
2	73	BA.5	FALSE
3	25	BA.5	TRUE
4	10	BA.2	FALSE
5	134	BA.5	TRUE
6	35	BA.5	TRUE
7	188	BA.5	FALSE
8	111	BA.5	FALSE

Supplementary Table 20. Event time and lineage, and whether the lineage was obtained by sequencing (Observed = TRUE) or imputed (Observed = FALSE), for COVID-19 study endpoints included in the analysis (from 7 to 188 days post D15) in the Pfizer-BioNTech Stage-2 Omicron + Prototype study arm.

	EventTime	Lineage	Observed
1	84	BA.5	TRUE
2	82	BA.5	TRUE
3	33	BA.2	TRUE
4	56	BA.5	TRUE
5	60	BA.5	TRUE
6	25	BA.2	TRUE

Supplementary Table 21. Event time and lineage, and whether the lineage was obtained by sequencing (Observed = TRUE) or imputed (Observed = FALSE), for COVID-19 study endpoints included in the analysis (from 7 to 188 days post D15) in the Pfizer-BioNTech Stage-2 Prototype study arm.

	EventTime	Lineage	Observed
1	54	BA.5	TRUE
2	81	BA.5	TRUE
3	78	BA.5	FALSE
4	166	BA.5	FALSE
5	133	BA.5	TRUE
6	33	BA.5	TRUE
7	16	BA.4	TRUE
8	49	BA.5	FALSE
9	33	BA.5	TRUE
10	76	BA.5	TRUE
11	78	BA.5	TRUE
12	135	BA.5	TRUE
13	26	BA.5	FALSE
14	82	BA.5	TRUE
15	174	BA.5	TRUE
16	65	BA.5	TRUE

Supplementary Table 22. Event time and lineage, and whether the lineage was obtained by sequencing (Observed = TRUE) or imputed (Observed = FALSE), for COVID-19 study endpoints included in the analysis (from 7 to 188 days post D15) in the Pfizer-BioNTech Stage-4 Omicron BA.1 + Prototype study arm.

	EventTime	Lineage	Observed
1	21	BA.5	FALSE
2	43	BA.5	FALSE
3	57	BA.5	FALSE
4	63	BA.5	TRUE
5	31	BA.5	TRUE
6	86	BA.5	TRUE
7	7	BA.5	TRUE
8	40	BA.5	TRUE

Supplementary Table 23. Event time and lineage, and whether the lineage was obtained by sequencing (Observed = TRUE) or imputed (Observed = FALSE), for COVID-19 study endpoints included in the analysis (from 7 to 188 days post D15) in the Pfizer-BioNTech Stage-4 Omicron BA.4/BA.5 + Prototype study arm.

	EventTime	Lineage	Observed
1	73	XBB.1.5	TRUE
2	85	XBB<ca>0.10	TRUE
3	53	BA.5	FALSE
4	63	BA.5	FALSE
5	16	BA.5	TRUE
6	93	XBB.1.5	TRUE
7	50	BA.5	TRUE

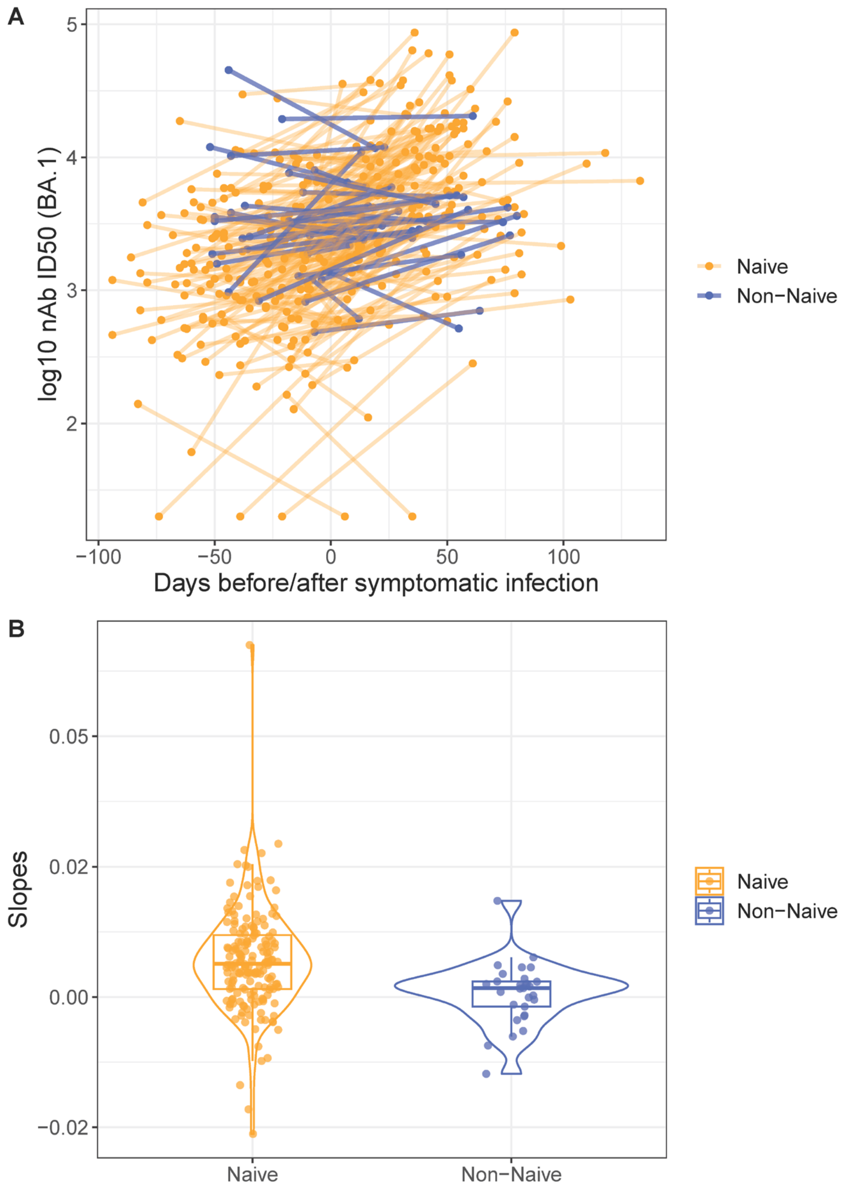

Supplementary Figure 29. A) BA.1 log10 titer before and after COVID-19 in naïve (yellow dots) and non-naïve (blue dots) cases. B) Violin plots of pre/post COVID-19 slope in BA.1 log10 titer for naive (yellow dots) and non-naive (blue dots) cases. Violin plots contain interior box plots with upper and lower horizontal edges the 25th and 75th percentiles of antibody level and middle line the 50th percentile, and vertical bars the distance from the 25th (or 75th) percentile of antibody level and the minimum (or maximum) antibody level within the 25th (or 75th) percentile of antibody level minus (or plus) 1.5 times the interquartile range. Each side shows a rotated probability density (estimated by a kernel density estimator with a default Gaussian kernel) of the data. Cases from 7 to 188 days post D15 are included (N=344 in naïve participants; N=58 in non-naïve participants). nAb-ID50, 50% inhibitory dilution neutralizing antibody titer. Source data are provided as a Source Data file.

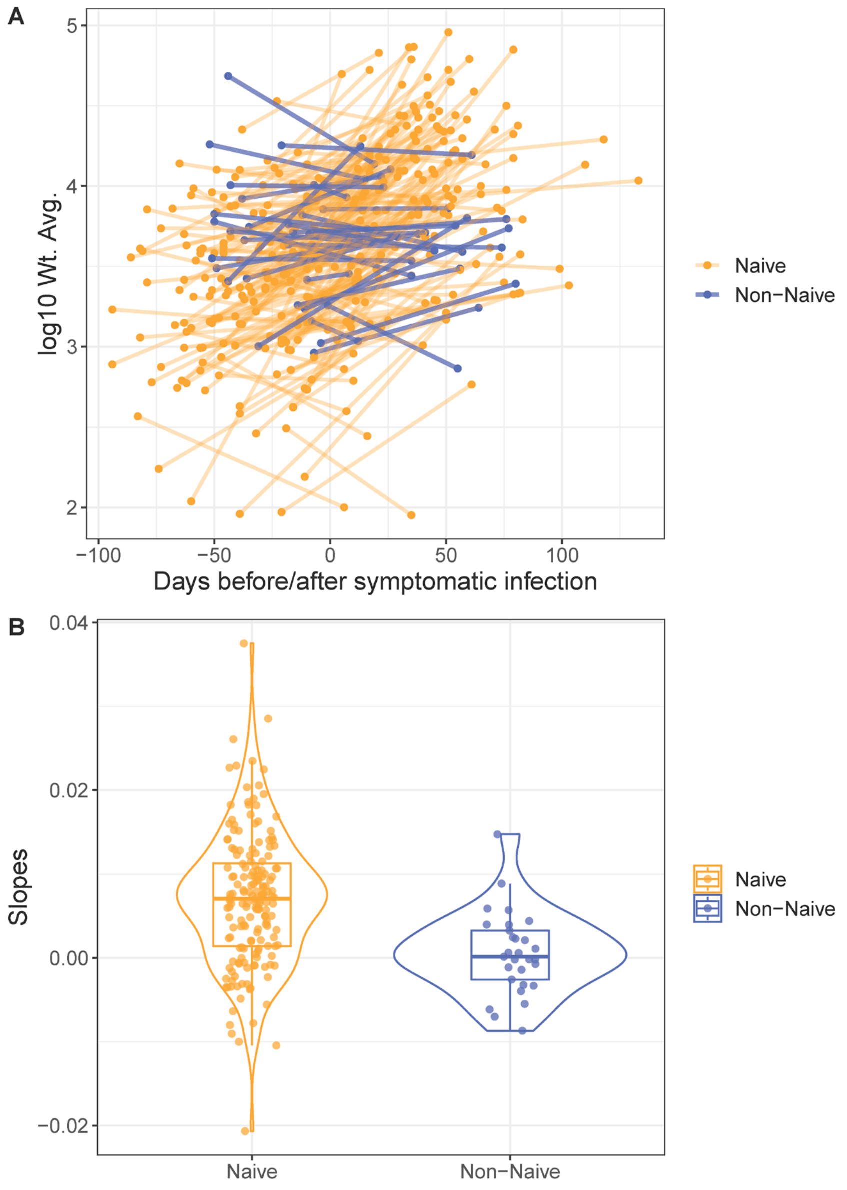

Supplementary Figure 30. A) Log10 weighted average (Wt. Avg) nAb titer before and after COVID-19 in naïve (yellow dots) and non-naïve (blue dots) cases. B) Violin plots of pre/post COVID-19 slope in log10 weighted average nAb titer for naive (yellow dots) and non-naive (blue dots) cases. Violin plots contain interior box plots with upper and lower horizontal edges the 25th and 75th percentiles of antibody level and middle line the 50th percentile, and vertical bars the distance from the 25th (or 75th) percentile of antibody level and the minimum (or maximum) antibody level within the 25th (or 75th) percentile of antibody level minus (or plus) 1.5 times the interquartile range. Each side shows a rotated probability density (estimated by a kernel density estimator with a default Gaussian kernel) of the data. Cases from 7 to 188 days post D15 are included (N=344 in naïve participants; N=58 in non-naïve participants). Wt. Avg. = Maximum diversity weighted geometric mean of the five titers Reference, Beta, Delta, Omicron BA.1, and Omicron BA.4/BA.5. Source data are provided as a Source Data file.

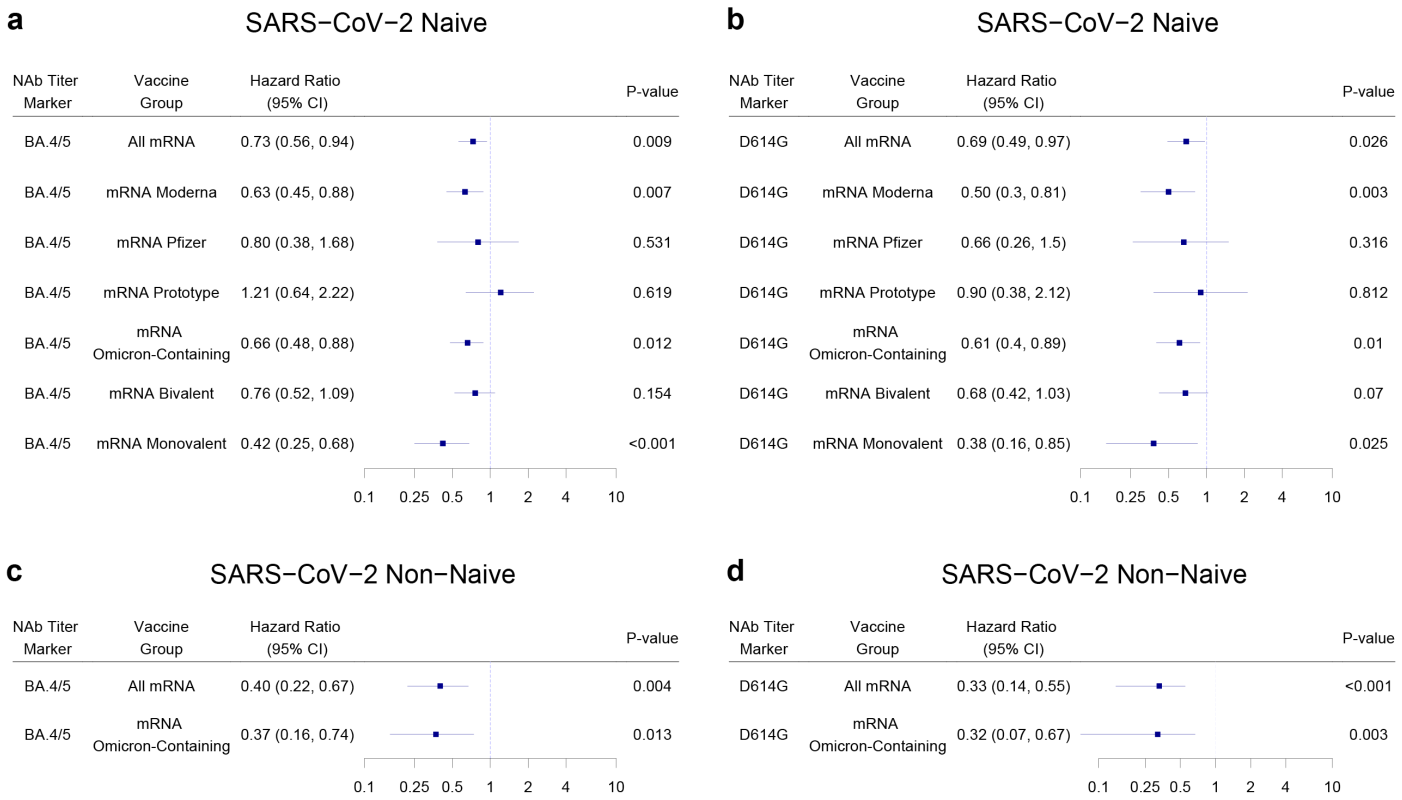

Supplementary Figure 31. Cox model covariate-adjusted, calendar time-based hazard ratios of BA.4/BA.5 COVID-19 per 10-fold increase in A, C) predicted-at-exposure BA.4/BA.5 titer (AU/ml) or B, D) predicted-at-exposure D614G titer (AU/ml), in A, B) SARS-CoV-2 naïve participants and in the designated subgroups, or C, D) SARS-CoV-2 non-naïve participants and in the designated subgroups, for COVID-19 endpoints from 7 to 188 days post D15. A) (All mRNA N=605) and in the designated subgroups (mRNA Moderna N=297, mRNA Pfizer-BioNTech N=122, mRNA Prototype N=101, mRNA Omicron-containing N=441, mRNA Bivalent N=336, mRNA Monovalent N=105), or C) non-naïve participants (All mRNA N=351) and in the designated subgroup (mRNA Omicron-containing N=281). B) (All mRNA N=629) and in the designated subgroups (mRNA Moderna N=306, mRNA Pfizer-BioNTech N=131, mRNA Prototype N=105, mRNA Omicron-containing N=459, mRNA Bivalent N=352, mRNA Monovalent N=107), or D) non-naïve participants (All mRNA N=356) and in the designated subgroup (mRNA Omicron-containing N=285). Point estimates, 95% bootstrap percentile confidence intervals (CIs), 2-sided bootstrap p-values are shown. P-values were not adjusted for multiple comparisons. Subgroup analyses were only conducted when the number of BA.4/BA.5 endpoints was equal to or exceeded 15, to ensure reasonable precision. AU, arbitrary units; nAb, neutralizing antibody.

Supplementary References

1. Fong Y, Dang L, Zhang B, et al. Neutralizing Antibody Immune Correlates for a Recombinant Protein Vaccine in the COVAIL Trial. ciae465, https://doi.org/10.1093/cid/ciae465. *Clinical Infectious Diseases* 2024.
2. Centers for Disease Control and Prevention. National Notifiable Diseases Surveillance System (NNDSS): Coronavirus Disease 2019 (COVID-19) 2021 Case Definition. Last reviewed August 24, 2021. Access date October 3, 2024. https://ndc.services.cdc.gov/case-definitions/coronavirus-disease-2019-2021/.
3. Branche AR, Rouphael NG, Diemert DJ, et al. Comparison of bivalent and monovalent SARS-CoV-2 variant vaccines: the phase 2 randomized open-label COVAIL trial. *Nat Med* 2023; **29**(9): 2334-46.
4. El Sahly HM, Baden LR, Essink B, et al. Efficacy of the mRNA-1273 SARS-CoV-2 Vaccine at Completion of Blinded Phase. *N Engl J Med* 2021; **385**(19): 1774-85.
5. Baden LR, El Sahly HM, Essink B, et al. Efficacy and Safety of the mRNA-1273 SARS-CoV-2 Vaccine. *N Engl J Med* 2021; **384**(5): 403-16.

Statistical Analysis Plan for
"Neutralizing antibody immune correlates by prior SARS-CoV-2 infection status in COVAIL trial recipients of an mRNA second COVID-19 vaccine boost"

Bo Zhang, Youyi Fong, Lauren Dang, Jonathan Fintzi, Shiyu Chen, Jing Wang, Chenchen Yu, Craig A. Magaret, Cindy Molitor, Bhavesh Borate, Sydney Busch, David Benkeser, Peter B. Gilbert, Dean Follmann

1. Introduction

COVAIL enrolled approximately 1250 adults in the U.S. previously vaccinated with a COVID-19 vaccine primary series.[1,2] Participants received a homologous or heterologous/variant boost from March 30 to October 28, 2022: Stage 1 was enrolled between 30 March to 06 May, 2022; Stage 2 was enrolled from 09 May to 23 May, 2022; Stage 3 was enrolled from 06 June to 13 June, 2022; and Stage 4 was enrolled from 04 October to 28 October, 2022. The randomization was stratified by age and history of confirmed prior SARS-CoV-2 infection, with the goal of approximately 45% of participants in each arm to include older adult (≥ 65 years of age) and at least 20% with prior infection history. Participants were randomized to one of 17 vaccine booster arms in four stages: Stage 1 is 6 Moderna mRNA vaccine arms (n=600 planned), Stage 2 is 6 Pfizer-BioNTech mRNA vaccine arms (n=300 planned), Stage 3 is 3 Sanofi recombinant protein vaccine arms (n=150 planned), and Stage 4 is 2 Pfizer-BioNTech bivalent mRNA vaccine arms (n=200 planned). Each of the first three stages includes an Ancestral strain prototype vaccine with the remainder of vaccine arms being variant vaccines. All study arms deliver a single booster except vaccine arm 3 in Stage 1 that delivers two bivalent mRNA boosters. Table 1 shows the COVAIL study schema.

Table 1: COVAIL study schema

Study Arms

	Arms	Vaccine Platform	Sample Size	Vaccine Candidate	Interval (weeks)*	Timing of First Dose	Timing of Second Dose
Stage 1	1	Moderna mRNA-1273	100	Prototype	≥16	D1	NA
	2		100	Beta + Omicron	≥16	D1	NA
	3		100	Beta + Omicron	≥16	D1	D57
	4		100	Delta + Omicron	≥16	D1	NA
	5		100	Omicron	≥16	D1	NA
	6		100	Omicron + Prototype	≥16	D1	NA
Stage 2	7	Pfizer/ BioNTech BNT162b2	50	Wildtype (Prototype)	≥16	D1	NA
	8		50	Beta + Omicron	≥16	D1	NA
	9		50	Omicron	≥16	D1	NA
	10		50	Beta	≥16	D1	NA
	11		50	Beta + Wildtype	≥16	D1	NA
	12		50	Omicron + Wildtype	≥16	D1	NA
Stage 3	13	Sanofi CoV2 preS dTM-AS03	50	Prototype	≥16	D1	NA
	14		50	Beta	≥16	D1	NA
	15		50	Beta+ Prototype	≥16	D1	NA
Stage 4	16^	Pfizer/ BioNTech BNT162b2 bivalent	100	Omicron BA.1 + Wildtype (Prototype)	≥16	D1	NA
	17^		100	Omicron BA.4/BA.5 + Wildtype (Prototype)	≥16	D1	NA

Blood samples were collected and stored from all study participants at D1, D15, D29, Month 3, 6, 9, 12. Relevant participant covariates include age, calendar time of enrollment, SARS-CoV-2 naïve/non-naïve status defined by the randomization strata of confirmed prior SARS-CoV-2 infection (no or yes), date of last prior vaccination before enrollment, and type of last prior vaccination. Sera samples were routinely evaluated for the presence of anti-N specific antibodies as a means to determining virus exposures at D1, D29, D91, and D181 and to confirm or annotate breakthrough infections in conjunction with self-reported and confirmed PCR infections during the course of the study.

Pseudovirus neutralization antibody titers (ID50) were measured for all Stage 1 and 2 mRNA booster arm participants against D614G, Delta/B.1.617.2, Beta/B.1.351, Omicron BA.1/B.1.1.529, and Omicron BA.4/BA.5 at multiple timepoints: baseline/enrollment (D1), 14 days after vaccination (D15), 28 days after vaccination (D29) 90 days (D91) after vaccination, 180 days (D181) after vaccination, 270 days (D271) after vaccination, and 365 days (D366) after vaccination, where Omicron BA.4/BA.5 was only measured in a subset at D15. For Arm 3 of Stage 1 (the only two-dose arm), ID50 titers were measured at D1, D15, D29 (prior to receipt of dose 2) and at 14, 28, 91, 181, 271, 365 days post-dose 2 (D57, D71, D85, D147, D237, D327, D422, respectively). For Stage 3 (Sanofi vaccine arms), ID50 titers against the same 5 strains were measured at D1, D15, D29, D91, D181, D271, D366. For Stage 4, ID50 titers against the same 5 strains were measured at D1, D15, D29, D91, D181. A central lab (Monogram) measured the titers against D614G, Delta, Beta, Omicron BA.1 and Omicron BA.4/BA.5 strains using a validated pseudovirus neutralization assay.

Neutralizing antibody (nAb) ID50 titer against BA.4/BA.5 was measured for all participants at D29 and for a subset at D15. In contrast, nAb ID50 titers against the other 4 strains was measured for all participants at D15. To use D15 as the key approximate peak time point for correlates assessment, all participants missing a D15 BA.4/BA.5 nAb ID50 value have their value imputed based on a linear regression with 2 predictors D29 log10 nAb ID50 against BA.4/BA.5 and the number of days between the D15 and D29 visit. A small number of participants were missing a D29 BA.4/BA.5 nAb ID50 value, for which this imputation approach will not work. However, these participants have an observed D15 BA.1 nAb ID50 value. For this small subset, the imputation of D15 BA.4/BA.5 nAb titer will be done based on a linear regression with sole predictor D15 log10 nAb ID50 against BA.1. Note that this imputation approach is only relevant for the one-dose arms; for Arm 3 (only 2-dose arm), the visit schedule is different, and no imputations are done. The resulting D15 nAb ID50 titer BA.4/BA.5 marker, observed for some and predicted for others, is analyzed as a correlate, to be able to include all six nAb ID50 titer markers at D15 in correlates analyses. The missing D15 nAb ID50 BA.4/BA.5 values were verified to be predicted with high accuracy, such that this imputation is expected to only have minor influence on the results.

A concordance study of D614G ID50 titer was conducted between the Monogram lab and the Duke University lab, which allows placement of the Monogram D614G ID50 titers on the scale of IU50/ml units (calibrated to the 20/136 WHO International Standard); this procedure was applied for previous immune correlates studies from the US Government COVID-19 Vaccine Correlates of Protection Program focusing on neutralizing antibodies against D614G.[3-7] The multiplicative constants placing Duke D614G ID50 units on the IU50/ml scale and Monogram D614G ID50 units on the IU50/ml scale were 0.242 and 0.0653, respectively. For the strains different from D614G, there is no International Standard, such that IU50/ml units do not exist. The COVAIL analyses use Monogram nAb ID50 assay data only, and all results are reported in Monogram ID50 units that are Arbitrary Units/ml (AU/ml). Where results are shown for the D614G strain, two axes may be used to show results both in Monogram assay D614G ID50 AU/ml units and in IU50/ml units, which will aid comparison with previous publications including for Moderna COVE.[3]

Participants who experience virologically-confirmed symptomatic COVID-19 starting 7 days after the D15 visit through to 188 days post D15 visit are referred to as COVID-19 cases, who are eligible for inclusion in the immune correlates analyses. Participants with no evidence of SARS-CoV-2 infection after D1 and through to the data cut date of 2023-07-23 are referred to as non-cases/controls, whose nAb ID50 data are included in immune correlates analyses. Correlates analyses either focus on eligible participants with D1 and D15 nAb ID50 data available, or, for the analyses of exposure-proximal correlates analyses, also use the ID50 data collected at additional time points, as detailed below.

This statistical analysis plan considers data analysis of the 13 one-dose mRNA vaccine arms, excluding study arms 3, 13-15 from consideration. Fong et al.[8] conducted a similar immune correlates analysis of the same nAb ID50 markers for the Sanofi vaccine arms 13-15, although that analysis was much more restricted in scope given the smaller sample size and much smaller number of COVID-19 cases. Where the same correlates objectives are assessed for the current article and Fong et al.,[8] the statistical analysis plans are harmonized, where Fong et al.[8] includes a statistical analysis plan for the analyses conducted for that article.

2. Antibody markers and sampling design for measuring antibody markers

The following antibody markers defined using D15 nAb ID50 titers, as well as D1 nAb ID50 titers for a subset of the markers, are considered for so-called peak time point antibody correlates analysis:

1. D1 log10 ID50 to D614G/Reference
2. D1 log10 ID50 to Delta
3. D1 log10 ID50 to Beta
4. D1 log10 ID50 to Omicron BA.1 (B.1.1.529)
5. D1 log10 ID50 to BA.4/BA.5
6. D1 maximal signal diversity weighted (MDW) score, calculated as the maximal signal diversity weighted average of antibody markers 1-5.[9]
7. D15 log10 ID50 to D614G
8. D15 log10 ID50 to Delta
9. D15 log10 ID50 to Beta
10. D15 log10 ID50 to Omicron BA.1 (B.1.1.529)
11. D15 log10 ID50 to BA.4/BA.5
12. D15 MDW score, calculated as the maximal signal diversity weighted average of antibody markers 7-11.[9]
13. D15 log10 ID50 to D614G – D1 log10 ID50 to D614G
14. D15 log10 ID50 to Delta – D1 log10 ID50 to Delta
15. D15 log10 ID50 to Beta – D1 log10 ID50 to Beta
16. D15 log10 ID50 to Omicron BA.1 – D1 log10 ID50 to Omicron BA.1
17. D15 log10 ID50 to BA.4/BA.5 – D1 log10 ID50 to BA.4/BA.5
18. D15 MDW score – D1 MDW score

The variables 13.-18. measure log10 fold-rise in nAb ID50 titer from pre-booster to post-booster vaccination.

The limit of detection (LoD) of the Monogram assay is 40; all values below the LoD are assigned value ID50 = 20. The ID50 units are not calibrated to International Standards, given that an International Standard is only available for the D614G strain. The antigen-specific upper limits of quantification (ULOQ) are used to censor marker values in inferential analyses but not descriptive analyses, which means that before data analysis ID50 values > ULOQ are set to ULOQ. The exception is that for exposure-proximal correlates analyses that use antibody decay models and focus on D15 marker values for predicting antibody trajectories over time, actual nAb ID50 values are used without setting values > ULOQ to the ULOQ. This is done to aid accuracy and precision of the predicted antibody trajectories over time. The ULOQs are 198904, 14858, 35879, 27104, 58293 AU/ml for D614G, Delta, Beta, BA.1, and BA.4/BA.5, respectively. All assay limit information is listed at: https://github.com/CoVPN/correlates_reporting2/blob/master/assay_metadata/covail_assay_metadata.csv. No ULOQ truncation is applied for the MDW score marker.

2.1 Vaccine groups of interest for studying vaccine efficacy and correlates of protection

There are different ways to meaningfully group vaccine booster arms together into paired groups for comparison, where we focus on the following, all of which restrict to the one-dose mRNA vaccine arms:

1. [mRNA Moderna vs. mRNA Pfizer-BioNTech] 1A. Moderna vaccines (arms 1-2, 5-6 pooled) vs. 1B. Pfizer-BioNTech vaccines (arms 7-9, 12 pooled) restricting to one-dose arms with common inserts in Moderna and Pfizer-BioNTech vaccines
2. [mRNA Prototype vs. mRNA Omicron-Containing] 2A. Prototype vaccines (arms 1, 7 pooled) vs. 2B. Omicron-containing vaccines (2, 4-6, 8, 9, 12, 16, 17 pooled) restricting to one-dose arms
3. [mRNA Bivalent vs. mRNA Monovalent] 3A. Bivalent Omicron-containing vaccines (arms 2, 4, 6, 8, 12, 16, 17 pooled) vs. 3B. Monovalent Omicron-containing vaccines (arms 5, 9 pooled) restricting to one-dose arms

Pfizer-BioNTech vaccine arms 10 and 11 are excluded in comparison 1. because the Beta and Beta+Widtype/Prototype vaccines were not studied for the Moderna vaccine. In addition, Pfizer-BioNTech vaccine arms 16 and 17 are excluded in the mRNA Moderna vs. mRNA Pfizer-BioNTech booster comparison to retain a closer head-to-head comparison of vaccine-insert content. In what follows, the bracketed labels are used to indicate the three pairs of groups defined above, i.e., [mRNA Moderna vs. mRNA Pfizer-BioNTech], [mRNA Prototype vs. mRNA Omicron-Containing], [mRNA Bivalent vs. mRNA Monovalent].

2.2 Definitions of three time periods for capturing COVID-19 outcome cases included in correlates analyses

For the COVID-19 outcome, “Non-cases” are participants who never register a COVID-19 primary endpoint at any time during follow-up and are never anti-N seropositive during follow-up. There are three groups of COVID-19 cases that are considered in data analyses: Cases occurring >= 7 days after the D15 visit and diagnosed with COVID-19 by 188 days post D15 visit (“D15_7to188 Cases”), Cases occurring >= 7 days after the D15 visit and diagnosed with COVID-19 by 91 days post D15 visit (“D15_7to91 Cases”, i.e., “boost-proximal cases”), and cases occurring between 92 days post D15 visit and diagnosed with COVID-19 by 188 days post D15 visit (“D15_92to188 Cases”, i.e., “boost-distal cases”). Note that the union of D15_7to91 Cases and D15_92to188 Cases equals the D15_7to188 Cases.

For implementing the analysis/computer code, the three time periods for counting COVID-19 endpoint cases, and for defining time-to-event variables on the study time scale, are defined using the following rules:

1. The time origin is the D15 visit date. For all three time periods, a COVID-19 endpoint must occur >= 7 days post D15 visit date to be included.
2. Boost-proximal (D15_7to91 COVID-19) cases include cases with onset date through 91 days post D15 visit.
3. Boost-distal (D15_7to188 COVID-19) cases include cases through 188 days post D15 visit, where 188 days is selected instead of 181 days to include more COVID-19 cases through ~6 months post marker measurement accounting for visit window variability.

For D15 immune marker correlates analyses, follow-up for COVID-19 is right-censored at the date of receipt of the second dose (in all analyses), to avoid the complexity of a systematic perturbation in antibody response partway through follow-up for one study arm but not for others, which could

complicate the interpretability of results. Descriptives show that zero COVID-19 endpoints are evaluable for correlates analyses for Arm 3 participants. Therefore Arm 3 participants contribute essentially no information about correlates. Accordingly, Arm 3 is excluded from the correlates analyses.

For assessing nAb ID50 titers as correlates of the COVID-19 endpoint, participant follow-up is right-censored by the first event among (1) receipt of a second dose (applicable for study arm 3 as noted above), (2) early termination, (3) receiving an out-of-study boost, and (4) the data cut date of 2023-07-13. In addition, for a given time-period of analysis D15_7to91, D15_92to188, or D15_7to188, right-censoring is by reaching 91 days, 188 days, and 188 days post D15 visit without a COVID-19 endpoint, respectively. Moreover, to be included in correlates analyses a participant must not have an eligibility deviation (based on the variable ***eligibility_deviation*** in the data set) and they must have available D15 nAb ID50 titer data.

2.3 Figures and tables to describe the log10 ID50 markers for COVID-19 cases vs. non-cases

Branche et al.[1,2] reported immunogenicity characterization of the vaccine booster arms for the log10 ID50 markers against the five different SARS-CoV-2 strains D614G, Delta, Beta, BA.1, BA.4/BA.5. Therefore, to present new information, the figures for this correlates SAP focus on showing results for cases vs. non-cases. Plots are arranged to show side by side distributions, in the order from left to right of Non-cases, D15_7to91 Cases, D15_92to188 Cases, D15_7to188 Cases.

Generally, it is of interest to show results separately for the markers measured to each of the five SARS-CoV-2 strains, as well as for a sixth marker – the maximal signal diversity weighted (MDW) score marker. For each of the 5 strains separately and the MDW score marker (6 total), it is of interest to make plots focusing on different sets of vaccine arms and comparisons:

a. Plot for the 13 one-dose mRNA booster arms pooled
b. Plot portraying the comparison of [mRNA Moderna vs. mRNA Pfizer-BioNTech]
c. Plot portraying the comparison of [mRNA Prototype vs. mRNA Omicron-Containing]
d. Plot portraying the comparison of [mRNA Bivalent vs. mRNA Monovalent]

where b., c., d. are defined in Section 2.1 as three pairwise pooled-arm comparisons.

Separate plots are made for studying the markers measured at D1, the markers measured at D15, and the fold-rise paired differences in the markers (D15 time point minus D1 time point). In addition, for completeness, the plots are also done for each of the study arms separately.

For all 13 one-dose mRNA vaccine arms pooled, scatterplots are made to study the correlations of the 6 nAb ID50 titer markers at each given time point (D1, D15, D29, D91), using a pairs plot, with Spearman rank correlations. For each of the 6 nAb ID50 titer markers, pairs plots are used to study correlations of readouts across all available time points of measurement. COVID-19 with lineages BA.4 and BA.5 were captured separately. Because BA.4 and BA.5 have the same Spike protein sequence and all the booster vaccines are Spike-insert vaccines, the BA.4 and BA.5 lineages are pooled together as a COVID-19 outcome genotype, referred to as BA.4/BA.5. If there are multiple SARS-CoV-2 lineages with >= 25 evaluable COVID-19 cases and for which nAb ID50 was measured against the given lineage, then additional plots will be done to highlight how antibody response to the case-causing lineage/genotype compare between non-cases and cases. That is, for each case-causing genotype, four side-by-side violin/box plots are made, the first for antibody measured against the specific genotype for non-cases,

and the subsequent three plots for antibody measured against the specific genotype for D15_7to91 Cases, D15_92to188 Cases, D15_7to188 Cases caused by the given specific genotype. Based on descriptive statistics (Tables 3, 4), the only qualifying lineage with >= 25 evaluable COVID-19 cases is BA.4/BA.5. Therefore, the plots are made for this genotype. For these plots, all COVID-19 endpoints have a lineage value, most of them observed and some of them imputed by GISAID as noted in Section 6.6. Different plotting symbols are used for observed-lineage cases and for imputed-lineage cases.

The markers are always plotted as log10 ID50 values, where the y-axis tick marks are placed on the ID50 scale (that is, the plotting labels use anti-log10 transformation).

"ID50 titer" is used as the label for each marker, which is more appropriate than IU/ml or IU50/ml, because the readout is in International Units (IU) only for the D614G strain. However, the ID50 titer label does not apply well to the MDW score marker; for this marker the label "Wt. Avg" (as the MDW score marker is a weighted average of the 5 strain-specific readouts) is used.

Note that every marker with ID50 titer < 40 AU/ml (the assay LOD) is assigned the value of 20 AU/ml, which is done before computing the MDW score marker.

Tables of nAb ID50 titer results at each time point

Summaries of the immunogenicity data will be reported in tables. In particular, the tables will include the following information for the ID50 pseudo-virus neutralization antibody marker at each of the time points: D1, D15, D29, D91. As noted, the sole 2-dose arm, Arm 3, is excluded from the tables given the different measurement schedule and the fact that right-censoring at the second dose implies inclusion of this arm would not provide significant information for the correlates analyses.

- For ID50 measured against each strain D614G, Delta, Beta, BA.1, BA.4/BA.5, the estimated percentage of participants defined as responders (i.e., ID50 titer > 40 AU/ml) with the corresponding 95% CIs using the Clopper-Pearson method.
- Geometric mean titers (GMTs) of ID50 against each strain D614G, Delta, Beta, BA.1, BA.4/BA.5 will be summarized along with their 95% CIs using the t-distribution approximation of log-transformed concentrations/titers. These are shown for antigen-specific markers and for the MDW score.
- Geometric mean titer ratios (GMTRs) of ID50 are defined as the geometric mean of individual titers (post-vaccination D15 or D29 or D91/pre-vaccination D1).
- GMTRs will be summarized with 95% CI (t-distribution approximation) for the D15 or D29 or D91 value compared to the D1 value.
- The ratios of GMTs for ID50 will be estimated between groups with the two-sided 95% CIs calculated using t-distribution approximation of log-transformed titers/concentrations, where the groups compared are vaccine recipient non-cases vs. vaccine recipient breakthrough COVID-19 cases occurring within each of three periods of COVID-19 endpoint onset D15_7to91, D15_92to188, D15_7to188. These results are shown for the 5 antigen-specific markers and for the MDW score.
- The differences in the positive response frequencies between groups (for antigen-specific markers, but not for the MDW score) will be computed along with the two-sided 95% CIs by the

Wilson-Score method without continuity correction[10] (the groups for comparison are as described in the previous bullet).

Tables will be provided separately following the objectives outlined as a.-e. above, for a. all 13 one-dose mRNA vaccine arms pooled (Arms 1-2, 4-12, 16-17) and c.-e. each of the 6 pooled groups of interest defined in Section 2.1. In addition, in the above list "cases" refers to COVID-19 cases not accounting for lineage information of the case. Moreover, similarly to the construction of figures, the tables also are done restricting COVID-19 cases to BA.4/BA.5. As for the figures, all COVID-19 endpoints have a lineage value, most of them observed and some of them imputed by GISAID as noted in Section 6.6.

2.4 Estimation of overall vaccine effects on COVID-19 for informing the final design of the immune correlates analyses

For each of the three two-group comparisons listed in Section 2.1, for each group, a point estimate of the cumulative incidence of COVID-19 over time starting 1 day after enrollment through to 188 days post D15 visit will be plotted. The cumulative incidence curves are estimated by hazard-based TMLE,[11] which provides a way to adjust for baseline covariates and to allow for covariate-dependent right-censoring. While the study is randomized, covariate-adjustment is useful because enrollment and follow-up was not fully concurrent across the vaccine arms, such that adjusting for calendar time is relevant. However, because the four stages of enrollment did not overlap in their calendar periods of follow-up, for many analyses it is not possible to adjust for calendar period as a baseline covariate. Accordingly, instead an exogenous force of infection score is adjusted for defined based on a data base of COVID-19 incidence in the U.S. during the period of COVAIL follow-up, as noted in Section 6.1. In addition, where possible Cox-model based analyses are conducted using the calendar time scale, which provides additional partial correction for differences in calendar time. These Cox model analyses adjust for baseline risk score (if predictive enough with CV-AUC > 0.55), and analyses that pool over the naïve and non-naïve groups also adjust for naïve/non-naïve status.

Super-learner is used to generate initial estimates of the conditional censoring distribution and to generate initial estimates of the conditional outcome regression. The Super Learner library includes both parametric and nonparametric algorithms as specified in Table 2. If a candidate algorithm involves tuning parameters, then the tuning parameter is selected based on a 5-fold cross validation. Each method includes adjustment for baseline risk score B built via super-learning as described in Section 7.1, the FOI score F, and, for analyses that pool over the naïve and non-naïve subgroups, baseline naïve vs. non-naïve status as specified in Table 3. The analyses will be implemented using the CFsurvival package available via http2://github.com/tedwestling/CFsurvival

Table 2. Super Learner library of regression models for estimation of the conditional survival function of COVID-19 event, conditional survival function of censoring, and the propensity score for TMLE estimation of the counterfactual cumulative incidence of the COVID-19 endpoint

**Conditional survival function of COVID event**	
survSL.km	Kaplan-Meier estimator
survSL.cox	Cox model

survSL.rfsrc	Survival random forest
survSL.gam	Generalized additive model
**Conditional survival function of censoring**	
survSL.km	Kaplan-Meier estimator
survSL.cox	Cox model
survSL.rfsrc	Survival random forest
survSL.gam	Generalized additive model
**Propensity score**	
SL.mean	Mean
SL.glm	Generalized linear model

Super-learner is used to generate initial estimates of the conditional censoring distribution and to generate initial estimates of the conditional outcome regression. The Super Learner library includes both parametric and nonparametric algorithms as specified in Table 2. Each method includes adjustment for the FOI score F and the baseline risk score B built via super-learning as described in Section 6.1, and two-way interactions of these terms, as well as for naïve/non-naïve status for analyses that pool over the naïve and non-naïve subgroups (see Table 5). The candidate algorithms in the Super Learner library consider various adjustments for time, as shown in Table 2. The TMLE analyses will be implemented using the *survtmle* R package available at CRAN.

Table 2. Super Learner library of regression models for estimation of the COVID-19 outcome regression and the right-censoring regression for TMLE estimation of cumulative incidence of the COVID-19 endpoint in each of two pairs of pooled study arm groups

**Algorithms**	**Screens / Tuning Parameters**
SL.mean	None
SL.glm	Low-collinearity and (All, Lasso, LR)[2]
SL.glm.interaction	Low-collinearity and (Lasso, LR)
SL.glmnet	All with (alpha = 0, 0.33, 0.67, 1)
SL.nnet	Low-collinearity and (Lasso, LR) (size=2,5)
SL.ksvm	Low-collinearity and (Lasso, LR) (kernel = “rbfdot”, “polydot”)
SL.ranger	All with (balance = yes, no)
SL.gam (only include if >= 50 endpoints	Low-collinearity and (Lasso, LR)
SL.gbm (only include if >= 50 endpoints)	Low-collinearity and (Lasso, LR)

SL. polymars (only include if >= 50 endpoints)	Low-collinearity and (Lasso, LR)
SL.xgboost (only include if >= 50 endpoints)	Low-collinearity and All and (maxdepth, shrinkage, balance[3])= (2, 0.1, yes) (2, 0.1, no) (4, 0.1, yes) (4, 0.1, no)

[1] All continuous and ordinal covariates are pre-standardized to have empirical mean 0 and standard deviation 1.

[2] **All** = include all variables; **Lasso** = include variables with non-zero coefficients in the standard implementation of SL.glmnet that optimizes the lasso tuning parameter via cross-validation; **Low-collinearity** = do not allow any pairs of quantitative variables with Spearman rank correlation > 0.90; **LR** = Univariate logistic regression Wald test 2-sided p-value < 0.10.

[3] Covariate balancing (if requested) is done using option scale_pos_weight in SL.xgboost and option case.weights in SL.ranger.

Cumulative incidence ratios over time [CIRs(t)] will be estimated by the ratio (group 1/group 2) of these TMLE cumulative incidence estimators. Influence-curve based variance estimators of each cumulative incidence is used, and the delta method applied to obtain the variance estimator of the log cumulative incidence ratio. Point estimates and 95% pointwise transformed Wald CIs for cumulative incidence curves and CIR(t) curves will be plotted. Note that while COVID-19 endpoints prior to 7 days post D15 are not included in correlates analyses, they are included in the cumulative incidence plots, to provide a more complete description of overall vaccine effects over time that provides context for the immune correlates analyses. The cumulative incidence plots include vertical marks at D22, D91, and D181. For point estimates, 95% CIs, and p-values for comparing cumulative incidence at D91 and at D181 between groups, the analysis restricts to the same cohort and endpoint time frame (counting COVID-19 cases starting 7 days after D15) in which the immune correlates analyses are assessed. Similarly, the Cox model estimates and inferences comparing groups use the same cohort and time frame in which the immune correlates analyses are done.

For completeness, for each study arm separately, the same cumulative incidence plotting is done. Given the much smaller sample sizes, these analyses implement *survtmle* only including the baseline risk score B in the outcome regression model and the censoring model, excluding the gam learners in the Super Learner library, and not including any pairwise interaction terms.

To summarize overall vaccine effects, the COVID-19 hazard ratio comparing each pair of groups during each of the three time periods D15_7to91, D15_92to188, and D15_7to188 will be reported as a point estimate, 95% CI, and 2-sided p-value for whether the hazard ratio departs from unity. The analysis is done with a proportional hazards model using calendar time as the time scale (thus allowing flexible modeling of COVID-19 incidence over calendar time in the nonparametric estimation of the baseline hazard function), with time origin March 30, 2022. These analyses include separate baseline hazards for naïve and non-naïve enrolled participants, where naïve/non-naïve status is coded by the variable ***Infstat*** that includes both anti-N serotesting data at enrollment and self-report. In addition, these analyses adjust for the FOI score and the baseline risk score B (Table 5).

For each of the nine comparisons defined by comparison type and time-period, the point estimate of the hazard ratio and the lower limit of the 95% CI provide guidelines informing whether to conduct the natural direct/indirect effects mediation correlates of protection analyses. The following guideline is used for whether to conduct mediation analysis for the COVID-19 endpoint:

- Natural direct/indirect effect mediation correlates of protection analyses for the COVID-19 outcome are only conducted for a given pair of study groups and over a given time-period D15_7to91, D15_92to188, and D15_7to188, if the point estimate of the COVID-19 hazard ratio indicates at least a 30% difference in hazard ratio with the 95% CI excluding unity.

The result is that mediation CoP analysis is only done for the mRNA Prototype vs. mRNA Omicron-Containing pooled study arm comparison.

3. Objectives for assessing ~peak time point (D15) immune markers as immune correlates for COVID-19 ignoring lineage/sequence

3.1 List of Objectives for the COVID-19 primary outcome

The study objectives for the COVID-19 primary outcome are assessed in participants regardless of SARS-CoV-2 naïve/non-naïve status, for occurrence of the first COVID-19 endpoint and not accounting for lineage/sequence information of the COVID-19 endpoint. However, significant interactions in **Obj. peak 2.** will imply reporting results separately by naïve and non-naïve status.

Obj. peak 1. [Univariable CoR] To assess each of the 12 antibody markers at D15 as a correlate of risk (CoR) of COVID-19

Obj. peak 2. [Univariable CoR] To assess whether and how the D15 CoRs in 1. are modified by SARS-CoV-2 naïve/non-naïve status

Obj. peak 3. [Univariable CoR] To assess whether and how the D15 CoRs in 1. are modified by the D1 value of the same antibody marker

Obj. peak 4. [Univariable CoR] To assess whether and how the D15 CoRs in 1. are modified by the mRNA booster vaccine being (1) [mRNA Moderna vs. mRNA Pfizer-BioNTech], (2) [mRNA Prototype vs. mRNA Omicron-Containing], or (3) [mRNA Bivalent vs. mRNA Monovalent] (groupings of study arms defined in Section 2.1).

Obj. peak 5. [Univariable CoR durability] Assess each D15 marker as a CoR in the two time periods D15_7to91 and D15_92to188 (defined below) and evaluate whether the CoR differs in the two time periods.

Obj. peak 6. [Multivariable CoR ensemble machine learning] To build models based on input sets of immune markers (and baseline demographic variables) that best predict whether a vaccine recipient experiences the COVID-19 endpoint. These sets distinguish between classes of variables with different meanings, including the FOI score F, the baseline risk score B, D1 markers, absolute-level D15 markers,

fold-rise D15/D1 markers, SARS-CoV-2 naïve/non-naïve status, score markers combining information across markers, and type of vaccine (the three booster group indicators defined in Section 2.1) together with time between last vaccination before enrollment and enrollment. The models compare prediction accuracy of models with different sets of input variables. This objective generates an 'estimated optimal surrogates (EOS)' for each input variable set,[12] where an EOS combines information across the variables in an input set, defining the surrogate as an individual's predicted probability of COVID-19 from an estimated-optimal predictive model.

Obj. peak 7. [Univariable CoP controlled risk] To assess each D15 marker as a controlled risk CoP of COVID-19 (defined in Gilbert et al.[13]) by estimating the controlled-risk-by-ID50 curve for each of the 6 booster groups defined in Section 2.1 and pooling over all these 6 groups (i.e., all one-dose mRNA booster arms). This objective includes sensitivity analysis to quantify the robustness of the CoP to potential unmeasured confounding of the effect of the D15 marker on COVID-19. The term 'controlled risk CoP' may be confusing given the lack of a comparison group in this analysis; alternatively, this analysis can be referred to as a 'CoR-contrast analysis.'

Obj. peak 8. [Univariable CoP controlled risk ratio] For each D15 marker, assess it as a CoP by estimation/inference for the controlled risk ratio (defined in Gilbert et al.[13]) for each of the three pairwise booster group contrasts defined in Section 2.1.

Obj. peak 9. [Univariable CoP mediation] For each qualifying D15 marker as specified at the end of Section 2.3, and for each of the three vaccine effects on COVID-19 of interest defined in Section 2.1, assess it as a natural effects mediator of the vaccine effect through the antibody marker. The analysis reports point and 95% CI estimates of Non-Marker-mediated VE (i.e., the Natural Direct Effect), Marker-mediated VE (i.e., the Natural Indirect Effect), and the Proportion mediated (i.e., the proportion of the vaccine effect mediated through the marker).

All the above objectives are addressed, except **Obj. peak 6.** is not addressed, because immunoassays measuring additional immune functions (including Fc effector function markers, IgA markers, IgG subclass markers, T-cell markers) need to be measured before conduct of integrative statistical learning.

The naïve/non-naïve status variable is defined by the previous infection status variable in the primary COVAIL data set (***Infstat*** variable). The peak time point correlates objectives are conducted based on the following cohorts. Objectives peak 1-3, 7-9 are conducted for (1) all 13 one-dose mRNA booster arms pooled (Arms 1-2, 4-12, 16-17), and (2)-(7) for each of the 6 groups 1A, 1B, 2A, 2B, 3A, 3B defined in Section 2.1. Objectives peak 4-5 are conducted for all 13 one-dose mRNA booster arms pooled. Objective peak 6 is conducted for all 16 one-dose booster arms pooled. For objectives that are assessed separately to time periods D15_7to91 and D15_92to188, the criteria evaluated in Table 4 are used to determine which analyses are conducted based on the requirement of a sufficient number of COVID-19 endpoints.

3.2 Time period of follow-up for correlates analyses

As introduced in Section 2.2, correlates analyses are done for three time periods of follow-up post D15, each of which counts COVID-19 endpoints starting 7 days after D15: (1) follow-up through 6 months post

D15 visit ("D15_7to188 cases"); (2) through 91 days post D15 visit ("D15_7to91 Cases", i.e., "boost-proximal cases"); and (3) cases occurring between 92 days post D15 visit and diagnosed with COVID-19 by 188 days post D15 visit ("D15_92to188 Cases", i.e., "boost-distal cases"). For each analysis for each time-period, if there a total of fewer than 25 evaluable COVID-19 endpoints available to include in the analysis, then the analysis will be canceled. The cut-point of 91 days is chosen because vaccine protection is known to wane over time and restricting to the first 3 months post D15 focuses on the period of maximal booster vaccine immunity. Moreover, including follow-up only through 188 days post D15 visit gives a simple interpretation in terms of results through ~6 months post booster, and literature suggests that booster efficacy is near zero by 6 months post booster.

4. Objectives for assessing immune markers over time as exposure-proximal correlates for COVID-19 not accounting for SARS-CoV-2 genotype (lineage/sequence)

The following exposure-proximal correlates objectives are assessed:

Obj. exp-prox 1. To assess each of the 6 nAb titer markers over time as an exposure-proximal CoR of COVID-19

Obj. exp-prox 2. To assess whether and how the CoRs in 1. are modified by SARS-CoV-2 naïve/non-naïve status

Obj. exp-prox 3. To assess whether and how the CoRs in 1. are modified by the D1 value of the same antibody marker

Obj. exp-prox 4. To assess whether and how the CoRs in 1. are modified by the mRNA booster vaccine being (1) [mRNA Moderna vs. mRNA Pfizer-BioNTech], (2) [mRNA Prototype vs. mRNA Omicron-Containing], or (3) [mRNA Bivalent vs. mRNA Monovalent] (groupings of study arms defined in Section 2.1).

The exposure-proximal correlates objectives are conducted based on the following cohorts. Objectives exposure-proximal 1-3 are conducted for all 13 one-dose mRNA booster arms pooled and for each of the 6 groups defined in Section 2.1, including all follow-up through 188 days post booster dose. Objective exposure-proximal 4 peak is conducted for all 13 one-dose mRNA booster arms pooled.

5. Objectives for assessing ~peak time point (D15) immune markers as immune correlates for COVID-19 accounting for lineage/sequence of the COVID-19 endpoint

For a given analysis unit defined by pooled vaccine arm and one of the three time periods, for each lineage with more than 25 COVID-19 endpoints, the following objectives are addressed, with purpose to explore how much lineage match or mismatch matters for correlates. As noted above in Table 4, the lineage BA.4/BA.5 qualifies with enough endpoints for many of the analyses.

Obj. peak gt 1. Assess the lineage-specific D15 antibody markers as CoR/CoP against COVID-19 with the specific lineage

Obj. peak gt 2. Assess D614G D15 antibody markers as CoR/CoP against COVID-19 with the specific lineage, and evaluate whether matching the antibody to the COVID-19 outcome lineage improves the correlate

The peak time point genotype-specific correlates objectives are conducted based on the same cohorts as for the peak time point correlates analyses not accounting for lineage.

6. Objectives for assessing immune markers over time as exposure-proximal correlates for COVID-19 accounting for lineage/sequence/genotype of the COVID-19 endpoint

As for other analyses, the analyses include all 13 one-dose mRNA vaccine arms 1-2, 4-12, 16-17 pooled. The analyses for a given group are conducted for each lineage with at least 25 COVID-19 endpoints in the group, addressing the following objectives. The analysis comparing a pair of groups within one of the three comparisons listed in Section 2.1 is included if there at least 50 COVID-19 endpoints in the D15_7to188 time-period pooling over the two groups under consideration.

Obj. exp-prox gt 1. Assess the lineage-specific antibody markers as exposure-proximal CoRs against COVID-19 with the specific lineage

Obj. exp-prox gt 2. Assess D614G antibody markers as exposure-proximal CoRs against COVID-19 with the specific lineage, and evaluate the quality of this correlate compared to the lineage-directed correlate

The above objectives are conducted for the same cohorts for which lineage is not accounted for.

Table 3 lists numbers of evaluable COVID-19 endpoints overall and by lineage for the three different time periods of COVID-19 endpoints. Because nAb titers were only measured to BA.4/BA.5 among the lineages listed in Table 3, breakthrough cases with BA.4 or BA.5 are of greatest interest, as it enables to the genotype-specific objectives. Table 4 summarizes numbers of lineage-specific COVID-19 endpoints by analysis grouping and conclusions on which lineages are supported and selected for immune correlates analysis. The pairs of comparisons of interest are denoted 1A vs. 1B, 2A vs. 2B, 3A vs. 3B. The analysis comparing a pair of groups within one of the three comparisons listed in Section 2.1 is included if there at least 50 COVID-19 endpoints in the D15_7to188 time-period pooling over the two groups under consideration. Figures 1-4 show the All COVID-19 endpoint counts (not accounting for lineage) graphically.

The conclusions from Table 4 and Figures 1-4 are as follows:

1. mRNA vaccines pooled: All, BA.4/BA.5 CoR analyses are conducted for the D15_7to91 and D15_7to188 periods and All, BA.4/BA.5 CoR analyses for the D15_92to188 period, where ‘All’ means that the analysis not accounting for lineage is conducted.
2. [1A. Moderna mRNA vaccines] pooled (Arms 1-2, 5-6): All COVID-19 CoR analyses conducted for the D15_7to91 period; All, BA.4/BA.5 for the D15_7to188 period and All BA.4/BA.5 CoR analyses for the D15_92to188 period; CoP vs. 1B. for All and BA.4/BA.5 for the D15_7to91 and D15_7to188 periods.

3. [1B. Pfizer-BioNTech mRNA vaccines] pooled (Arms 7-9, 12): CoR All COVID-19 analyses are conducted for the D15_7to188 period; CoP vs. 1A. for All and BA.4/BA.5 for the D15_7to91 and D15_7to188 periods.
4. [2A. mRNA Prototype] monovalent vaccines pooled (Arms 1, 2): CoR analyses for All COVID-19 and BA.4/BA.5 for D15_7to188; CoP analyses vs. 2B are conducted for All, BA.4/BA.5 for D15_7to91 and D15_7to188 and All for D15_92to188.
5. [2B. mRNA Omicron-containing] vaccines pooled: CoR same as 1; CoP vs. 2A for All, BA.4/BA.5 for D15_7to91 and D15_7to188 and All for D15_92to188.
6. [3A. mRNA Bivalent] Omicron-containing vaccines pooled: CoR analyses for All COVID-19, BA.4/BA.5 for the D15_7to91 and D15_7to188 periods and All COVID-19 for the D15_92to188 period; CoP vs. 3B for All COVID-19 D15_7to91 and All, BA.4/BA.5 COVID-19 for D15_92to188.
7. [3B. mRNA Monovalent] Omicron vaccines pooled: No CoR analyses are conducted; CoP vs. 3A for All COVID-19 D15_7to91 and All, BA.4/BA.5 COVID-19 for D15_92to188.

Table 3. Lineages of COVID-19 primary endpoints by COVID-19 endpoint time-period and study arm

Time Period	Booster Arm	Number COVID-19 endpoints	Obtained sequence	BA.2	BA.4	BA.5	BA.4/ BA.5	XBB.1.5	XBBxca0.10	XZ
D15_7to188	1. 1 Dose Prototype (Moderna)	27	20	7	2	11	13	0	0	0
D15_7to91	1. 1 Dose Prototype (Moderna)	18	14	7	1	6	7	0	0	0
D15_92to188	1. 1 Dose Prototype (Moderna)	9	6	0	1	5	6	0	0	0
D15_7to188	2. 1 Dose Beta + Omicron (Moderna)	37	28	11	3	14	17	0	0	0
D15_7to91	2. 1 Dose Beta + Omicron (Moderna)	23	17	10	1	6	7	0	0	0
D15_92to188	2. 1 Dose Beta + Omicron (Moderna)	14	11	1	2	8	10	0	0	0
D15_7to188	3. 2 Dose Beta +	0	0	0	0	0	0	0	0	0

	Omicron (Moderna)									
D15_7to91	3. 2 Dose Beta + Omicron (Moderna)	0	0	0	0	0	0	0	0	0
D15_92to188	3. 2 Dose Beta + Omicron (Moderna)	0	0	0	0	0	0	0	0	0
D15_7to188	4. 1 Dose Delta + Omicron (Moderna)	28	23	8	4	10	14	0	0	1
D15_7to91	4. 1 Dose Delta + Omicron (Moderna)	20	18	8	2	7	9	0	0	1
D15_92to188	4. 1 Dose Delta + Omicron (Moderna)	8	5	0	2	3	5	0	0	0
D15_7to188	5. 1 Dose Omicron (Moderna)	25	18	2	1	15	16	0	0	0
D15_7to91	5. 1 Dose Omicron (Moderna)	11	6	2	1	3	4	0	0	0
D15_92to188	5. 1 Dose Omicron (Moderna)	14	12	0	0	12	12	0	0	0
D15_7to188	6. 1 Dose Omicron + Prototype (Moderna)	26	18	5	2	11	13	0	0	0
D15_7to91	6. 1 Dose Omicron + Prototype (Moderna)	16	11	5	1	5	6	0	0	0
D15_92to188	6. Dose Omicron +	10	7	0	1	6	7	0	0	0

	Prototype (Moderna)									
D15_7to188	7. Prototype (Pfizer-BioNTech 1)	16	12	0	1	11	12	0	0	0
D15_7to91	7. Prototype (Pfizer-BioNTech 1)	12	9	0	1	8	9	0	0	0
D15_92to188	7. Prototype (Pfizer-BioNTech 1)	4	3	0	0	3	3	0	0	0
D15_7to188	8. Beta + Omicron (Pfizer-BioNTech 1)	7	4	0	1	3	4	0	0	0
D15_7to91	8. Beta + Omicron (Pfizer-BioNTech 1)	6	4	0	1	3	4	0	0	0
D15_92to188	8. Beta + Omicron (Pfizer-BioNTech 1)	1	0	0	0	0	0	0	0	0
D15_7to188	9. Omicron (Pfizer-BioNTech 1)	8	3	0	0	3	3	0	0	0
D15_7to91	9. Omicron (Pfizer-BioNTech 1)	5	2	0	0	2	2	0	0	0

D15_92to188	9. Omicron (Pfizer-BioNTech 1)	3	1	0	0	1	1	0	0	0
D15_7to188	10. Beta (Pfizer-BioNTech 1)	9	5	0	2	3	5	0	0	0
D15_7to91	10. Beta (Pfizer-BioNTech 1)	6	4	0	2	2	4	0	0	0
D15_92to188	10. Beta (Pfizer-BioNTech 1)	3	1	0	0	1	1	0	0	0
D15_7to188	11. Beta + Prototype (Pfizer-BioNTech 1)	9	7	1	1	5	6	0	0	0
D15_7to91	11. Beta + Prototype (Pfizer-BioNTech 1)	7	6	1	1	4	5	0	0	0
D15_92to188	11. Beta + Prototype (Pfizer-BioNTech 1)	2	1	0	0	1	1	0	0	0
D15_7to188	12. Omicron + Prototype (Pfizer-BioNTech 1)	6	6	2	0	4	4	0	0	0
D15_7to91	12. Omicron + Prototype (Pfizer-	6	6	2	0	4	4	0	0	0

	BioNTech 1)									
D15_92to188	12. Omicron + Prototype (Pfizer-BioNTech 1)	0	0	0	0	0	0	0	0	0
D15_7to188	13. Prototype (Sanofi)	5	4	0	0	4	4	0	0	0
D15_7to91	13. Prototype (Sanofi)	4	3	0	0	3	3	0	0	0
D15_92to188	13. Prototype (Sanofi)	1	1	0	0	1	1	0	0	0
D15_7to188	14. Beta (Sanofi)	9	6	0	1	5	6	0	0	0
D15_7to91	14. Beta (Sanofi)	6	4	0	1	3	4	0	0	0
D15_92to188	14. Beta (Sanofi)	3	2	0	0	2	2	0	0	0
D15_7to188	15. Beta + Prototype (Sanofi)	8	5	0	1	4	5	0	0	0
D15_7to91	15. Beta + Prototype (Sanofi)	7	5	0	1	4	5	0	0	0
D15_92to188	15. Beta + Prototype (Sanofi)	1	0	0	0	0	0	0	0	0
D15_7to188	16. Omicron BA.1 + Prototype (Pfizer-BioNTech 2)	8	5	0	0	5	5	0	0	0
D15_7to91	16. Omicron	8	5	0	0	5	5	0	0	0

	BA.1 + Prototype (Pfizer-BioNTech 2)									
D15_92to188	16. Omicron BA.1 + Prototype (Pfizer-BioNTech 2)	0	0	0	0	0	0	0	0	0
D15_7to188	17. Omicron BA.4/5 + Prototype (Pfizer-BioNTech 2)	7	5	0	0	2	2	2	1	0
D15_7to91	17. Omicron BA.4/5 + Prototype (Pfizer-BioNTech 2)	6	4	0	0	2	2	1	1	0
D15_92to188	17. Omicron BA.4/5 + Prototype (Pfizer-BioNTech 2)	1	1	0	0	0	0	1	0	0

Table 4. Numbers of All-COVID-19 and lineage-specific COVID-19 endpoints by analysis grouping and conclusions on which lineages are supported and selected for immune correlates analysis (‘All’ refers to analyses of COVID-19 not accounting for lineage)

Analysis Grouping	Numbers of Observed Lineage-Specific COVID-19 Endpoints<br>(BA.2, BA.4, BA.5, BA.4/BA.5, XBB.1.5, XBBxca0.10, XZ) [Total]

D15_7to91 COVID-19 endpoints mRNA vaccines (Arms 1-2, 4-12, 16, 17)	(35, 11, 57, 68, 1, 1, 1) [106]<br>**All, BA.4/BA.5 qualify**
D15_92to188 COVID-19 endpoints mRNA vaccines (Arms 1-2, 4-12, 16, 17)	(1, 6, 40, 46, 1, 0, 0) [48]<br>**All, BA.4/BA.5 qualify**
D15_7to188 COVID-19 endpoints mRNA vaccines (Arms 1-2, 4-12, 16, 17)	(36, 17, 97, 114, 2, 1, 1) [154]<br>**All, BA.4/BA.5 qualify**
1A. D15_7to91 COVID-19 endpoints mRNA_Moderna (Arms 1-2, 5-6)	(24, 4, 20, 24, 0, 0, 1) [48]<br>**All qualify for CoR**<br>**All, BA.4/BA.5 qualify for CoP vs. 1B**
1A. D15_92to188 COVID-19 endpoints mRNA_Moderna (Arms 1-2, 5-6)	(1, 4, 31, 35, 0, 0, 0) [36]<br>**All, BA.4/BA.5 qualify for CoR**<br>**None qualify for CoP vs. 1B**
1A. D15_7to188 COVID-19 endpoints mRNA_Moderna (Arms 1-2, 5-6)	(25, 8, 51, 59, 0, 0, 0) [84]<br>**All, BA.2, BA.4/BA.5 qualify for CoR**<br>**All, BA.4/BA.5 qualify for CoP vs. 1B**
1B. D15_7to91 COVID-19 endpoints mRNA_Pfizer-BioNTech (Arms 7-9, 12)	(2, 2, 17, 19, 0, 0, 0) [21]<br>**None qualify for CoR**<br>**All, BA.4/BA.5 qualify for CoP vs. 1A**
1B. D15_92to188 COVID-19 endpoints mRNA_Pfizer-BioNTech (Arms 7-9, 12)	(0, 0, 4, 4, 0, 0, 0) [4]<br>**None qualify for CoR**<br>**None qualify for CoP vs. 1A**
1B. D15_7to188 COVID-19 endpoints mRNA_Pfizer-BioNTech (Arms 7-9, 12)	(2, 2, 21, 23, 0, 0, 0) [25]<br>**All qualify for CoR**<br>**All, BA.4/BA.5 qualify for CoP vs. 1A**
2A. D15_7to91 COVID-19 endpoints mRNA_Prototype (Arms 1, 7)	(7, 2, 14, 16, 0, 0, 0) [23]<br>**None qualify for CoR**<br>**All, BA.4/BA.5 qualify for CoP vs. 2B**
2A. D15_92to188 COVID-19 endpoints mRNA_Prototype (Arms 1, 7)	(0, 1, 8, 9, 0, 0, 0) [9]<br>**None qualify for CoR**<br>**All qualifies for CoP vs. 2B**

2A. D15_7to188 COVID-19 endpoints mRNA_Prototype (Arms 1, 7)	(7, 3, 22, 25, 0, 0, 0) [32]<br><br>**All, BA.4/BA.5 qualify for CoR**<br>**All, BA.4/BA.5 qualify for CoP vs. 2B**
2B. D15_7to91 COVID-19 endpoints mRNA_Omicron_containing (Arms 2, 3-6, 8, 9, 12, 16, 17)	(27, 6, 37, 43, 1, 1, 1) [73]<br><br>**All, BA.4/BA.5 qualify for CoR**<br>**All, BA.4/BA.5 qualify for CoP vs. 2A**
2B. D15_92to188 COVID-19 endpoints mRNA_Omicron_containing (Arms 2, 3-6, 8, 9, 12, 16, 17)	(1, 5, 30, 35, 1, 0, 0) [37]<br><br>**All, BA.4/BA.5 qualify for CoR**<br>**All qualifies for CoP vs. 2A**
2B. D15_7to188 COVID-19 endpoints mRNA_Omicron_containing (Arms 2, 3-6, 8, 9, 12, 16, 17)	(28, 11, 67, 78, 2, 1, 1) [110]<br><br>**All, BA.4/BA.5 qualify for CoR**<br>**All, BA.4/BA.5 qualify for CoP vs. 2A**
3A. D15_7to91 COVID-19 endpoints mRNA_Bivalent_Omicron (Arms 2, 4, 6, 8, 12, 16, 17)	(25, 5, 32, 37, 1, 1, 1) [65]<br><br>**All, BA.4/BA.5 qualify for CoR**<br>**All qualify for CoP vs. 3B**
3A. D15_92to188 COVID-19 endpoints mRNA_Bivalent_Omicron (Arms 2, 4, 6, 8, 12, 16, 17)	(1, 5, 17, 22, 1, 0, 0) [24]<br><br>**None qualify for CoR**<br>**None qualify for CoP vs. 3B**
3A. D15_7to188 COVID-19 endpoints mRNA_Bivalent_Omicron (Arms 2, 4, 6, 8, 12, 16, 17)	(26, 10, 49, 59, 2, 1, 1) [89]<br><br>**All, BA.4/BA.5 qualify for CoR**<br>**All, BA.4/BA.5 qualify for CoP vs. 3B**
3B. D15_7to91 COVID-19 endpoints mRNA_Monovalent_Omicron (Arms 5, 9)	(2, 1, 5, 6, 0, 0, 0) [8]<br><br>**None qualify for CoR**<br>**All qualify for CoP vs. 3A**
3B. D15_92to188 COVID-19 endpoints mRNA_Monovalent_Omicron (Arms 5, 9)	(0, 0, 13, 13, 0, 0, 0) [13]<br><br>**None qualify for CoR**<br>**None qualify for CoP vs. 3A**
3B. D15_7to188 COVID-19 endpoints mRNA_Monovalent_Omicron (Arms 5, 9)	(2, 1, 18, 19, 0, 0, 0) [21]<br><br>**None qualify for CoR**<br>**All, BA.4/BA.5 qualify for CoP vs. 3A**

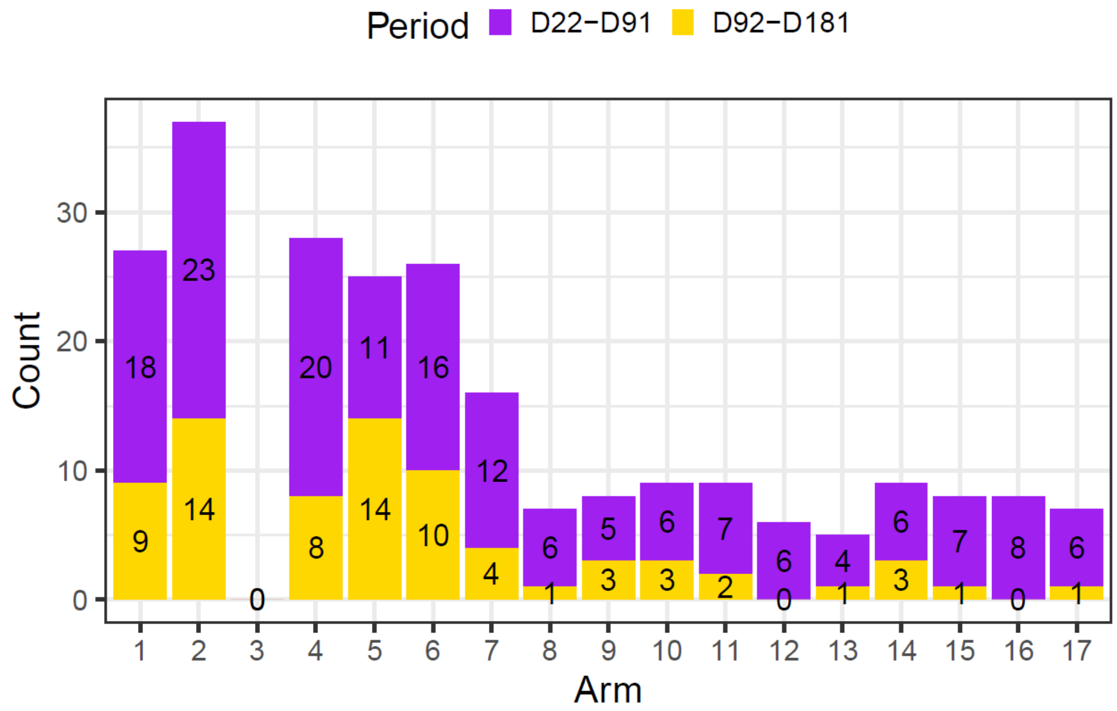

Figure 1. Numbers of COVID-19 primary endpoints evaluable for immune correlates analyses for the time periods D15_7to91 (label D22-D91) and D15_92to188 (label D92-D181) for capturing COVID-19 endpoints, where the time-period D15_7to188 has numbers of endpoints equal to sums from D15_7to91 and D15_92to188 (17 booster arms), although the 2-dose Arm 3 is excluded from immunogenicity and immune correlates analyses.

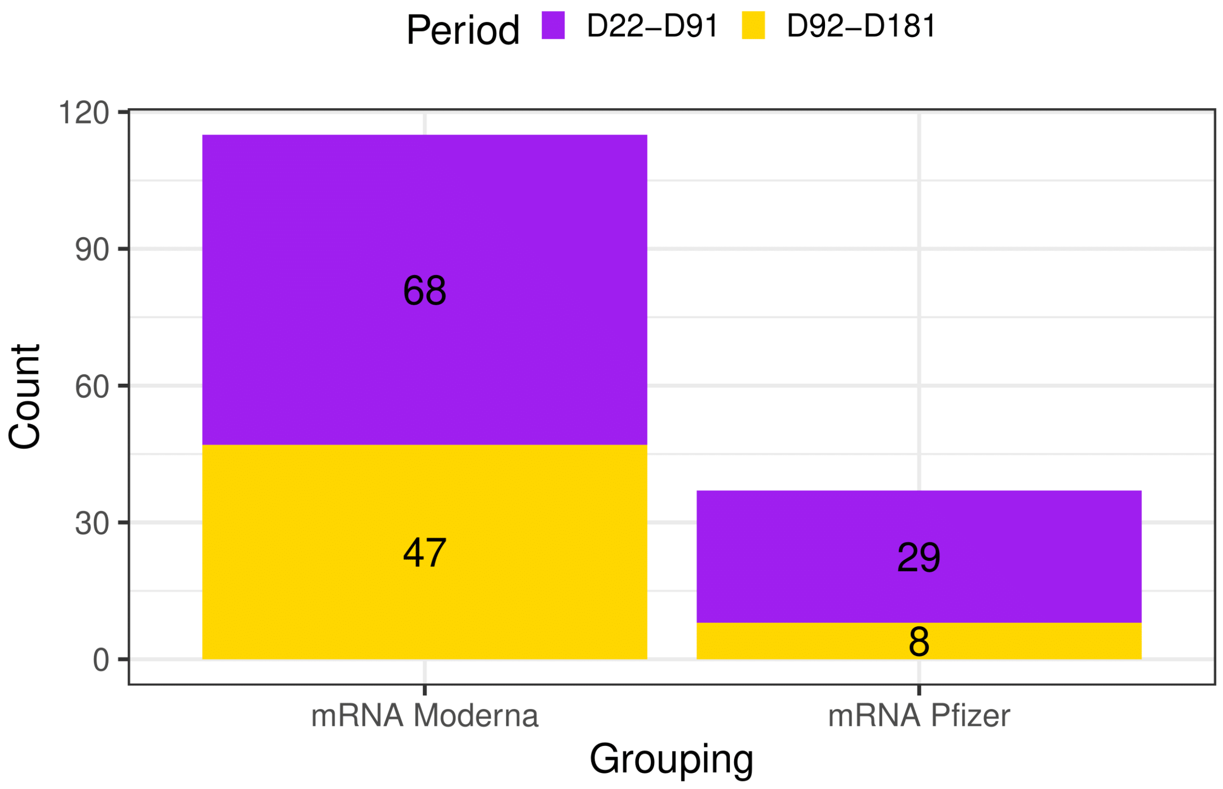

Figure 2. Same as Figure 1 for the booster arm groupings 1A. mRNA Moderna vaccines (Arms 1-2, 5-6) vs. 1B. mRNA Pfizer-BioNTech vaccines (Arms 7-9, 12). D22-D91 indicates the time period D15_7to91 and D92-D181 indicates the time period D15_92to188 for capturing COVID-19 endpoints.

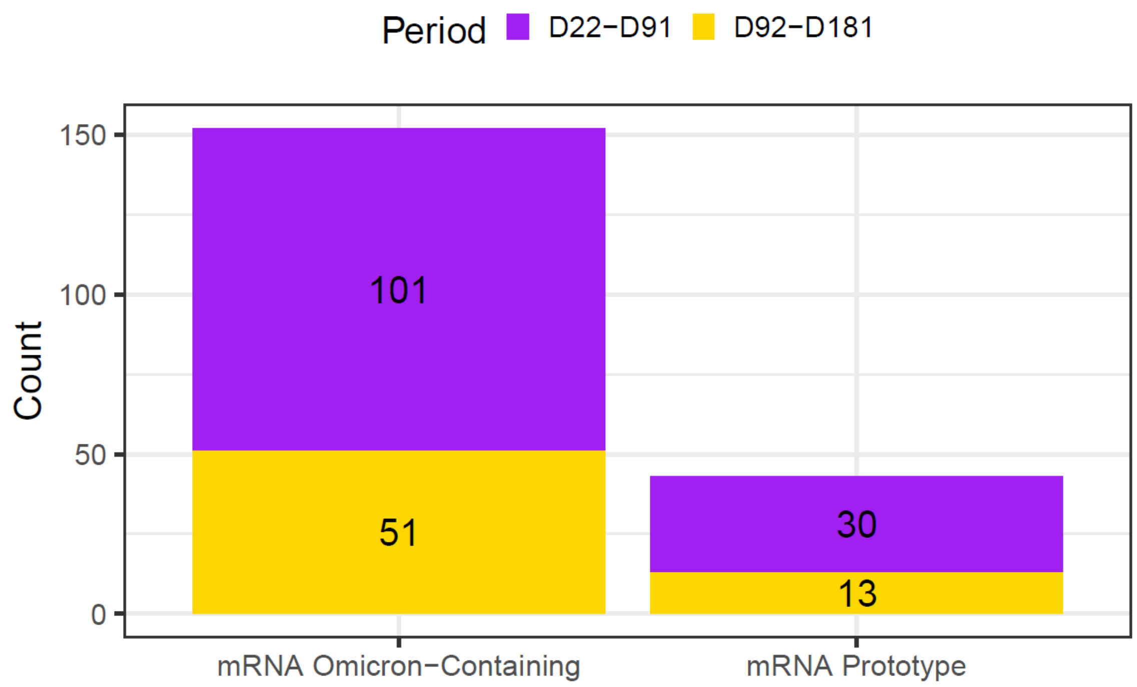

Figure 3. Same as Figure 1 for the booster arm groupings 2A. mRNA prototype vaccines (Arms 1, 7) vs. 2B. mRNA Omicron-containing vaccines (Arms 2, 4-6, 8, 9, 12, 16, 17). D22-D91 indicates the time period D15_7to91 and D92-D181 indicates the time period D15_92to188 for capturing COVID-19 endpoints.

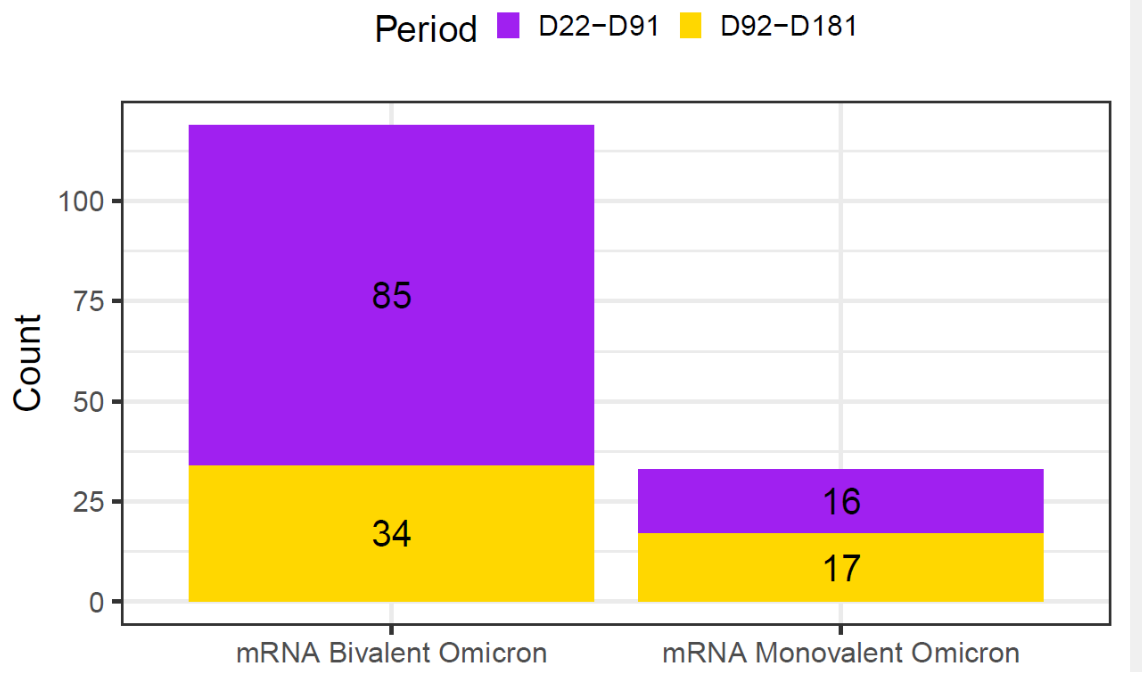

Figure 4. Same as Figure 1 for the booster arm groupings 3A. mRNA Bivalent Omicron-containing vaccines (Arms 2, 4, 6, 8, 12, 16, 17) vs. 3B. mRNA Monovalent Omicron-containing vaccines (Arms 5, 9). D22-D91 indicates the time period D15_7to91 and D92-D181 indicates the time period D15_92to188 for capturing COVID-19 endpoints.

6. Statistical analysis approaches to address the objectives

6.1 Baseline risk score development

A baseline risk score for best predicting occurrence of COVID-19 starting 7 days after the D15 visit will be built using cross-validated super learning pooling over all study participants, based on all demographic and vaccination history input variables, using a similar approach as taken for assessing immune correlates in the phase 3 trials through the US Government's COVID-19 Vaccine Correlates of Protection Program. In particular, the baseline risk score is defined as the logit of the predicted COVID-19 outcome

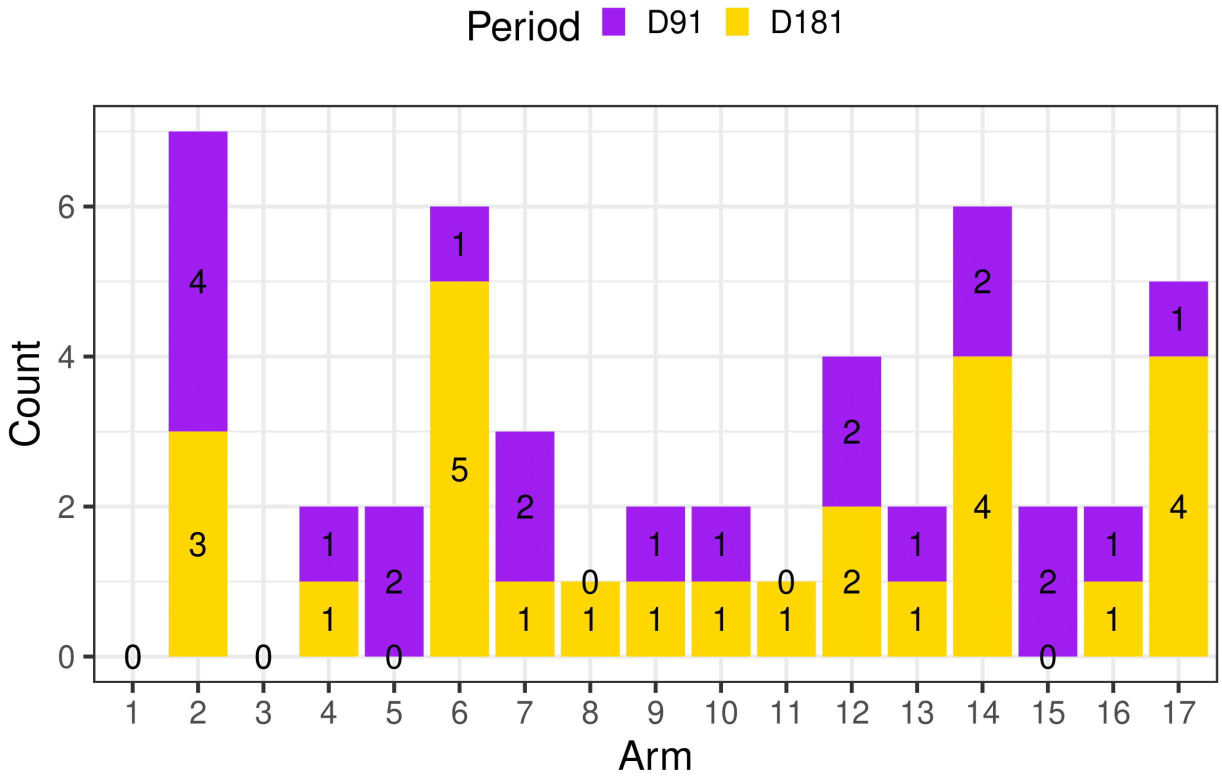

probability from a regression model estimated using the ensemble algorithm superlearner (i.e., stacking), where this logit predicted outcome is scaled to have empirical mean zero and empirical standard deviation one. The settings of superlearner (i.e., loss function, cross-validation technique, library of learners) that are used for implementation of superlearner for building a baseline risk score are described as follows.

The binary endpoint implementation of superlearner is used without accounting for right-censoring, with the needed details recapitulated as follows:

1. Pre-scale each quantitative and ordinal input variable to have empirical mean 0 and standard deviation 1.
2. Negative binomial log-likelihood loss is used.

3. Study participants lost to follow-up without ever experiencing a COVID-19 endpoint are excluded from the analysis, for the relevant time-period of analysis. For example, if analyses are done separately by time-period, then a participant lost to follow-up after 91 days post booster is included in the D15_7to91 analysis and excluded in the D15_7to188 and D15_92to188 analyses.
4. For analyses including all of follow-up, 5-fold cross-validation is used, with no more than floor(nv/6) input variables included in any given model/algorithm where nv is the number of evaluable COVID-19 endpoint cases. For analyses that restrict to time periods or otherwise restrict and the total number of COVID-19 endpoint cases with evaluable antibody marker data is less than 50, then leave-one-out cross-validation is used.
5. Learning algorithms (as listed in Table 2) are included with and without screening of variables. Screens used will be: 1) glmnet (lasso) pre-screening (with default tuning parameter selection), 2) logistic regression univariate 2-sided p-value screening (at level $p < 0.10$), and 3) high-correlation variable screening (described below). The adaptive algorithms (SL.xgboost, SL.gam, SL.gbm, SL.polymars) are only used with these screens, given that the limited number of endpoint cases may challenge use of these methods without variable screening. Moreover, the adaptive algorithms are not used if there are less than 50 endpoint cases. All the selected learners are coded into the SuperLearner R package available on CRAN. Table 6 lists the learner-screen combinations used as input algorithms to the Superlearner for development of the baseline risk score.
6. Include high-correlation variable screening, not allowing any pair of input variables to have Spearman rank correlation $r > 0.9$.
7. The superlearner is conducted averaging over 10 random seeds, to make results less dependent on random number generator seed.
8. Inverse probability of sampling (IPS) weights are not needed, given the measurement of PsV ID50 titer from all participants. If analyses include markers only measured in a participant subset, then IPS weights will be used.
9. Discrete-SL estimated models, derived using the learning algorithms specified in Table 2, will be used to compare the relative performance for each of the variable sets based off the estimated cross-validated area under the ROC curve (CV-AUC) with a 95% confidence interval.
10. Two levels of cross-validation are used:
    a. Outer level: CV-AUC computed over 5-fold cross-validation repeated 10 times to improve stability
    b. Inner level: 5-fold CV used to estimate ensemble weights, unless the number of COVID-19 endpoints is less than 50 in which case leave-one-out CV is used.
11. Results for comparing classification accuracy of different models are based on point and 95% confidence interval estimates of CV-AUC and difference in CV-AUC as a predictiveness metric.[14,15] Results are presented as forest plots of point and 95% confidence interval estimates similar to those used in Figure 3 of Neidich et al.[16] for HIV vaccines and in COVID-19 papers including Benkeser, Montefiori, McDermott et al.[7] CV-AUC is estimated using the R package *vimp* available on CRAN.

Table 6. All learner-screen combinations used as input to the Superlearner for development of the baseline risk score.

Learner	Screen*
SL.mean	All
SL.glm	All
	Glmnet
	univar_logistic_pvalue
	highcor_random
SL.glm.interaction	Glmnet
	univar_logistic_pvalue
	highcor_random
SL.glmnet	All
SL.gam	Glmnet
	univar_logistic_pvalue
	highcor_random
SL.xgboost	All
SL.ranger	All

***Screen details:**
all: includes all input variables
glmnet: includes variables with non-zero coefficients in the standard implementation of SL.glmnet that optimizes the lasso tuning parameter via cross-validation
univar_logistic_pval: Wald test 2-sided p-value in a logistic regression model < 0.10
highcor_random: If pairs of quantitative variables with Spearman rank correlation > 0.90, select one of the variables at random

The following baseline demographic input variables are included for building the risk scores: age in years, indicator of age ≥ 65, sex assigned at birth (Male/Female), ethnicity, race, the number of days from last prior vaccination until enrollment, the indicator that the number of days from last prior vaccination until enrollment is greater than the median value, and the type of last vaccine received prior to the booster.

The development of the baseline risk score involves training the superlearner including all study participants and making cross-validated (CV)-predictions. First-occurrence COVID-19 endpoints are included and counted starting 7 days post D15, with all COVID-19 endpoints counted ignoring lineage information. The CV-prediction performance of superlearner is characterized by point and 95% confidence interval estimates of the cross-validated area under the ROC curve (CV-AUC) and by point estimates of CV-ROC curves, which are calculated based on first occurrence COVID-19 endpoints starting 7 days post D15. The baseline risk score is defined as the logit of the predicted outcome probability from the superlearner model, where this logit predicted outcome is scaled to have empirical mean zero and empirical standard deviation one.

The baseline risk score, as long as it has some predictive capacity (defined as CV-AUC > 0.55), is adjusted for in all correlates analyses (both univariable marker and multivariable markers). SARS-CoV-2 naïve/non-naïve status, vaccination type information, and D1 ID50 marker data are not included as input

variables into the baseline risk score, as they are treated in a special way for various correlates analyses. The baseline risk score is included to capture exposure information or elevated risk information while allowing variables with a distinctive immunological meaning to be included separately, facilitating the ability to interpret associations/effects attached to these immunological variables.

In addition, to address the challenge that the four stages enrolled participants in different calendar periods, and incidence of COVID-19 can have secular trends, the study-time based correlates analyses also adjust for a force of infection score (FOI score) calculated with data from the Coronavirus Resource Center's database hosted by Johns Hopkins University (JHU): United States - COVID-19 Overview - Johns Hopkins (jhu.edu).

This database provides COVID-19 case numbers and incidence rates in the U.S. from 23 January 2020 through 09 March 2023. Each COVAIL study participant's FOI score is computed as the average of daily COVID-19 incidence rates (number of cases per 100,000 persons) over all days spanning from 7 days after their D15 visit through to 188 days post enrollment. The JHU database contains data at the geographic level of U.S. state and some territories, so the participants' geographic locations (at the level of state or the District of Columbia) were factored into the calculation.

FOI scores were not calculated for participants who missed their D15 visit, which does not impact correlates analyses given that D15 visit attendance is required for inclusion. Additionally, 200 participants had follow-up periods that extended beyond the JHU database's final day of 09 March 2023. (The latest 188 days post booster date for a COVAIL participant was 04 May 2023.) The case numbers for these missing dates were extrapolated by using the case numbers for those days from the prior year (10 March 2022 through 04 May 2022). In almost all geographic regions, these extrapolations were comparable with the preceding observed case counts, suggesting that they are a reasonably good fit.

The final FOI score used for covariate adjustment is standardized to have empirical mean 0 and empirical standard deviation 1.

6.2 Baseline covariates adjusted for in immune correlates analyses

Table 5 lists the baseline covariates that are adjusted for in all of the immune correlates analyses, where the FOI score is not adjusted for in calendar-time based exposure-proximal correlates analyses.

Table 5. Baseline covariates adjusted for in all CoR and CoP immune correlates analyses

B = Baseline risk score (if predictive with CV-AUC > 0.55, otherwise not adjusted for)<br>F = FOI score (adjusted for in study-time based survival analyses, not in calendar-time based survival analyses)<br>N = Naïve vs. non-naïve status defined by the ***Infstat*** variable that defines non-naïve as self-reported prior infection or positive N-Antibody test (adjusted for in analyses that pool over the naïve and non-naïve subgroups, not (obviously) for separate analyses of the naïve and non-naïve subgroups)

In addition, because the baseline risk score B is "black box" and may be challenging to interpret, we also conduct a sensitivity analysis that only adjusts for the single interpretable variable of whether age at enrollment is greater than or equal to 65 years of age. This sensitivity analysis is done for Cox model covariate-adjusted hazard ratios of COVID-19 per 10-fold increase in D15 BA.1 ID50 titer in naïve participants overall and by vaccine-platform designated subgroups, as well as in non-naïve participants overall and by vaccine-platform designated subgroups.

Descriptive figures for the confounders to adjust for listed in Table 5. The distribution of each variable in Table 5 will be displayed graphically for each of the study arms as well as for each group of study arms (1) all one-dose mRNA arms pooled and (2)-(7) groups 1A, 1B, 2A, 2B, 3A, 3C, with the latter plots arranged to compare 1A vs. 1B, 2A vs. 2B, 3A vs. 3B. Pooling over the 16 one-dose study arms, the distribution of the FOI score over calendar time will be plotted, with annotation by the four stages 1, 2, 3, 4 of enrollment.

6.3 Proportional hazards models for univariable correlates of risk

To assess whether a D15 log10 ID50 marker is a CoR, a proportional hazards regression model will be formulated. The analysis will be done using one of two operational time scales: (1) calendar time, with March 30, 2022, taken to be time zero; and (2) study time, with D15 visit date taken to be time zero. The study time analyses adjust for the variables listed in Table 5, whereas the calendar time analyses adjust for the variables listed in Table 5 excluding the FOI score (because the calendar time scale already provides partial confounding control due to secular trends in force of infection). Participants enter the risk set at D15 following their booster dose. For the purpose of Objective peak 1, the hazard for the model for participant *i* is

$$\lambda_i\ (t) = \lambda_0\ (t) \exp\{\boldsymbol{X}_i^T \boldsymbol{\beta} + \gamma_1 \log_{10}(Ab_i^{D15})\}\, I\{t > \tau_i^{D15}\},$$

where τ_i^{D15} is the calendar time of D15 post-booster for participant *i*, and $\boldsymbol{\beta}$ is a vector of parameters whose values correspond to log hazard ratios for baseline covariates that are adjusted for. For each D15 marker, we assess whether the marker is a CoR via a Wald test of the null that γ_1 is equal to 0. Uncertainty about model estimates will be quantified via 95% confidence intervals based on robust standard errors. If there are a substantial number of samples whose values are below the LoD, we will modify the model to split out the hazard for participants at or below the LoD from those whose titers were above the LoD,

$$\lambda_i\ (t) = \lambda_0\ (t) \exp\{\boldsymbol{X}_i^T \boldsymbol{\beta} + [\gamma_0 + \gamma_1 \log_{10}(Ab_i^{D15})] I\{Ab_i^{D15} > LoD\}\}\, I\{t > \tau_i^{D15}\},$$

Effect modification in Objectives peak 2.-4. is assessed by inclusion of an interaction term between the effect modifier of interest and D15 antibody marker. For instance, for Objective peak 2., we write the hazard as

$$\lambda_i\ (t) = \lambda_0\ (t) \exp\{\boldsymbol{X}_i^T \boldsymbol{\beta} + \gamma_1 \log_{10}(Ab_i^{D15}) + \gamma_2 Naive_i + \gamma_3 \log_{10}(Ab_i^{D15}) Naive_i\}\, I\{t > \tau_{i^{D15}}\},$$

and test for effect modification via a Wald test of the null hypothesis that γ_3 is equal to 0. We will also compute controlled risk curves at representative values of the effect modifier – naïve vs. non-naïve for

Objective peak 2., quartiles of D1 antibody value for Objective peak 3., vaccine booster type for Objective peak 4. – across the range of D15 antibody measurements.

If Objective peak 2. shows a significant interaction of Naïve status with D15 nAb titer, then the correlates analyses will be done separately in the Naïve and Non-naïve subgroups, and similarly for Objective exp-prox 2. In parallel, if Objective peak 3. shows a significant interaction of D15 nAb titer with D15 nAb titer, then correlates analyses will be done separately by D1 nAb titer marker tertiles subgroups, and similarly the Objective exp-prox 3. analyses will include modeling of how the correlate of risk depends on both D1 and D15 nAb titer.

Exploratory analyses may be conducted that assess D1, D15 nAb titer interactions separately in the Naïve and Non-naïve subgroups, as the correlation of Naïve status with D1 titer poses challenges to the simultaneous study of how Naïve status and D1 titer may modify the association of D15 nAb titer with COVID-19. Similarly, exploratory analyses may be conducted that assess D1, time-varying nAb titer interactions separately in the Naïve and Non-naïve subgroups, as the correlation of Naïve status with time-varying titer poses challenges to the simultaneous study of how Naïve status and D1 titer may modify the association of time-varying nAb titer with COVID-19.

6.4 Nonparametric analyses for univariable correlates of risk

The nonparametric methods are the same (or as similar as possible) as used in the US Government COVID-19 Vaccine Correlates of Protection Program's standard analysis of the phase 3 VE trials as specified in the correlates SAP (henceforth 'phase 3 correlates SAP'); the same methods are selected in order to aid interpretability of the results in a comparative fashion to the phase 3 results and especially to the Moderna COVE trial given the same type of vaccine administered in Stage 1 of COVAIL.

Objective peak 1. for the COVID-19 outcome is assessed using nonparametric threshold regression marginalizing over the baseline covariates specified in Table 5 with targeted minimum loss-based estimation, accounting for right-censoring of the COVID-19 failure time,[17] as implemented in the phase 3 correlates SAP. This analysis is done using *survtmle* assuming that the threshold-response regression is non-increasing in the marker threshold.

Objectives peak 2.-4. on effect modification are not assessed nonparametrically; the Cox modeling described above is used to address those objectives.

Objective peak 5. is assessed by implementing Objectives peak 1—4 separately for the two time periods D15_7to91 and D15_92to188, requiring at least 25 evaluable COVID-19 endpoints per time-period for Objectives peak 1—4. To evaluate whether a D15 marker CoR differs by time-period, a proportional hazards model is used to compare and assess interaction CoR slopes with COVID-19 events that occur in the D15_7to91 period versus events that occur in the D15_92to188 period. As a default, Objective peak 6 will only be done for the overall follow-up period D15_7to188, but if Objectives 1—4 provide clear evidence for differential CoRs by time-period then Objective peak 6 will be done separately for D15_7to91 and D15_92to188. This would provide a way to study how the correlate changes for outcomes over the first 3 months vs. for outcomes over 3 to 6 months.
Objective peak 7. is addressed for each of the D15 antibody markers using the semiparametric Cox-modeling and nonparametric controlled risk approach developed in Avi Kenny's PhD dissertation, as well as in Gilbert et al.,[13] that were used in the phase 3 correlates SAP. For D15 antibody marker subgroups defined by upper tertile vs. lower tertile, the semiparametric Cox-modeling method will be applied to

estimate E-values for the point estimate and of the 95% lower confidence limit for the controlled risk ratio (upper vs. lower tertile). Tertiles are defined for each nAb ID50 marker by the 1/3 and 2/3 percentile values pooling over the 13 one-dose mRNA vaccine arms (Arms 1-2, 4-12, 16-17), calculated separately for each marker defined by antigen and time point D1 or D15. E-values quantify robustness of controlled risk correlates of protection results to potential unmeasured confounding.

Objective peak 8. is addressed for each of the D15 antibody markers also using the semiparametric Cox and nonparametric controlled risk ratio modeling in the same way as used in the phase 3 immune correlates SAP for the analysis of a population with variability of the antibody marker in both the vaccine and placebo groups. For example, the statistical methods are harmonized with the statistical methods applied to the analysis of the VAT08 Sanofi phase 3 trial that predominantly enrolled SARS-CoV-2 non-naïve participants.

More specifically, the analyses for Objectives peak 7 and 8 will be conducted as follows. The controlled risk curve is of interest for each group of vaccine arms that is studied. Consider a comparison of the two vaccine groups, with the two groups denoted A=1 and A=0. For vaccine group A=1, consider the causal parameters the controlled risk curve and the controlled relative risk curve, defined as

$r_C(1, s) := P\{T(1, s) <= tF\}$

$RR_C(1, s) := P\{T(1, s) <= tF\} / P\{T(0) <= tF\}$.

Analogous parameters are defined for the other vaccine group for comparison,

$r_C(0, s) := P\{T(0, s) <= tF\}$

$RR_C(0, s) := P\{T(0, s) <= tF\} / P\{T(1) <= tF\}$.

Here $r_C(a, s)$ is the probability of endpoint occurrence by time tF in the hypothetical / counterfactual scenario in which all trial participants are assigned to (pooled) vaccine group A=a and have their immune marker level S set to s, for each of a=0 and a=1. Also, T(a) is the time to failure event for the hypothetical / counterfactual scenario in which all trial participants are assigned to vaccine group A=a (without assignment of the immune marker).

We describe the methodology for estimation of $r_C(1, s)$ and $RR_C(1, s)$, noting that the same methodology will be applied in the same way for $r_C(0, s)$ and $RR_C(0, s)$. Under our approach, the value of $RR_C(1, s)$ is assumed to be monotone non-decreasing in s; in other words, relative risk can only potentially be improved by setting greater marker levels. The extent to which the marker plays a role in determining risk and relative risk can be determined by the degree of flatness of the graph of $r_C(1, s)$ and $RR_C(1, s)$ versus s.

Now, $P(T(0) <= tF) = E_X[P(T <= tF | X, A=0)]$ whenever $I(T(0) <= tF)$ and A are independent given a vector X of baseline covariates, and $P(A=1 | X) > 0$ almost surely. Under this assumption, the controlled relative risk $RR_C(1, s)$ at level s can be identified using the fact that

$r_C(a, s) = risk1(tF|a, s) := E_X[P(T <= tF, J=1|S=s, X, A=1)]$

whenever $I(T(a, s) <= tF)$ and S are independent given A=a and a vector X of baseline covariates, and $P(S=s | A=a, X) > 0$ almost surely (for each a=0 and a=1).

For each D15 marker discretized into a binary marker High (S=1) vs. Low (S=0) (S =1 indicates the upper tertile vs. S =0 the lower tertile), the same approach as used in Gilbert et al.[13] is used to estimate, with a 95% CI,

$$RR_C(1; 0, 1) := RR_C(1, 1) / RR_C(1, 0).$$

The same analysis in Gilbert et al.[13] is conducted to compute an E-value for the point estimate and for the lower 95% confidence limit of $RR_C(1; 0, 1)$, which provide a sensitivity analysis to unmeasured confounding. This is repeated for A = 0: $RR_C(0; 0, 1) := RR(0, 1) / RR(0, 0)$. In addition, for each D15 marker treated as a quantitative marker, $RR_C(1, s)$ and $RR_C(0, s)$ are each estimated with a 95% CI and 95% estimated uncertainty interval.

For controlled relative risk, the parameter of interest is a surface, defined as

$$RR_C(1,0; s1, s0) := 1 - P\{T(1, s1) <= tF\} / P\{T(0, s0) <= tF\} = 1 - r_C(1, s1) / r_C(0, s0).$$

While of scientific interest to estimate the whole surface $RR_C(1,0; s1, s0)$, we restrict attention to assignments with s1=s0, in which case the surface simplifies to a curve $RR_C(1,0; s, s)$. The results for $RR_C(s, s)$ assess an average controlled direct effect where a value near zero for marker values in general supports the quality of the antibody marker as an immune correlate. The idea here, replete in the surrogate and mediation literature going back to Prentice,[18] is that for a good surrogate, if both treatment arms are assigned to have the same value of the intermediate outcome, then there is no effect of the treatment. To quantify this notion, the analysis will also estimate a summary measure quantifying nearness of the average controlled direct effects to zero:

$$ACD := \exp \{ \int [\log_{10}(RR_C(1,0; s, s))]^2 \}.$$

with the integral starting at the median of S in the group A=0 and increasing to the minimum of the 95th percentiles of S in the A=1 group and in the A=0 group. These ranges may be adjusted based on the distributions of the markers observed.

Plotting results for controlled risk and controlled relative risk analyses

For each immune marker at D1, D15, and fold-rise, the following output is planned:

- Cox-model method plotting of point, 95% CI, and 95% EUI estimates of each curve $r_C(1, s) = P\{T(1, s) <= tF\}$ and $r_C(0, s) = P\{T(0, s) <= tF\}$ in side by side panels or on the same panel
- Cox-model method plotting of point, 95% CI, and 95% EUI estimates of each curve $RR_C(1, s) = P\{T(1, s) <= tF\} / P\{T(0) <= tF\}$ and $RR_C(0, s) = P\{T(0, s) <= tF\} / P\{T(1) <= tF\}$ in side by side panels or on the same panel
- Cox-model method plotting of point and 95% CI estimates of the controlled direct effects curve $RR_C(1,0; s, s)$
- Cox-model method point and 95% CI estimates of the ACD (as a summary measure of surrogate endpoint value
- Nonparametric monotone-constrained method plotting of point, 95% CI, and 95% EUI estimates of each curve $r_C(1, s) = P\{T(1, s) <= tF\}$ and $r_C(0, s) = P\{T(0, s) <= tF\}$ in side by side panels or on the same panel

- Nonparametric monotone-constrained method plotting of point and 95% CI estimates of each curve $RR_C(1, s) = P\{T(1, s) <= tF\} / P\{T(0) <= tF\}$ and $RR_C(0, s) = P\{T(0, s) <= tF\} / P\{T(1) <= tF\}$ in side by side panels or on the same panel
- Nonparametric monotone-constrained method plotting of point and 95% CI estimates of the controlled direct effects curve $RR_C(1,0; s, s)$
- Nonparametric monotone-constrained method point and 95% CI estimates of ACD (as a summary measure of surrogate value)

Objective peak 9. is addressed for each of the D15 antibody markers using the nonparametric mediation analysis of Benkeser et al.[19] the same as specified in the phase 3 immune correlates SAP, where again a slightly different version of the analysis is needed to accommodate the fact that the antibody markers vary for both groups being compared. The mediation analyses are considered for each of the three study group contrasts defined in Section 2.1. As stated at the end of Section 2.3, the natural direct/indirect effect mediation analyses are only conducted for a given pair of groups over a given time-period D15_7to91, D15_92to188, and D15_7to188 if the point estimate of the COVID-19 hazard ratio indicates at least a 30% difference in hazard ratio and the 95% CI excluded unity. The mediation analysis yields point and 95% confidence interval estimates of the natural direct effect (the non-Marker-mediated vaccine effect), the natural indirect effect (the Marker-mediated vaccine effect), and the proportion of the vaccine effect mediated through the antibody marker.

6.5 Analysis of exposure-proximal correlates of risk

The analysis of exposure-proximal CoR takes a modular approach for assessing D15 antibody markers as CoRs in which we substitute the observed D15 marker value with a subject-specific time-varying predicted antibody curve for each participant. As such, the model for exposure-proximal CoR consists of linear mixed effects (LME) sub-model for antibody responses, and a semiparametric proportional hazards sub-model conditioned on a predictable time-varying antibody trajectory for each participant. We develop the modeling approach in the context of assessing whether a marker is an exposure-proximal CoR. Uncertainty about proportional hazards model estimates is quantified via 95% confidence intervals based on robust standard error estimates.

Hypothesis tests for goodness of proportional hazards model fit will be conducted using the Grambsch and Therneau method[20] as implemented in the R function *cox.zph* in the *survival* library.

Modeling antibody kinetics

We formulate a linear mixed effects (LME) model for longitudinal measurements of $\log_{10}$ antibody titers, which for most arms will be fit to measurements at days 14 (D15), 28 (D29), 90 (D91), and 180 (D181) post-vaccination. The model has fixed effects for days since D15, prior infection assessed by N-positivity at D1, and the interaction of prior infection and time since D15. We will only use each participant's data prior to a possible breakthrough infection or out of study vaccination (infection detection or COVID-19 endpoint) in fitting the model. Participants who are determined to have an asymptomatic infection between the D22 and D91 visits will only contribute their D15 measurement. Participants who are determined to have been asymptomatically infected between D91 and D181 will only contribute measurements through the earlier of D91 or their time of symptomatic infection. This restriction will be evaluated in simulations to assess risk of bias prior to data analysis. The model will be fit separately for

each study arm and each marker. In sensitivity analyses, we will also adjust for the interaction of D1 $\log_{10}$ antibody titer with time since D15. Other covariates may be included in sensitivity analyses. We will implement the following procedure to predict each participant's $\log_{10}$ antibody titer trajectory conditional on their measurements preceding breakthrough infection.

1. Fit the LME model to the $\log_{10}$ antibody titer measurements from all participants. Each participant only contributes antibody data as described above.
2. Compute the expected $\log_{10}$ antibody for participant at daily increments conditional on their $\log_{10}$ antibody titer measurements prior to any breakthrough infection. Here, we obtain the fitted values conditional on the random intercept for each participant. Participants who are determined to have been asymptomatically infected between D22 and D91 will have their trajectory from D92 onward predicted based on their D91 measurement and the rate of decay estimated based on baseline seropositive participants.

In the event more than 10% of measurements are below the limit of detection, we will modify the model to impute samples below the LoD. The modified model takes the form of a Tobit model, which integrates over the distribution of samples below the LoD under a Gaussian assumption. Clear outliers may be removed if it is deemed appropriate, with documentation of reasons for removal.

Proportional hazards sub-model

To assess whether a predicted log10 ID50 marker is a CoR, a second stage proportional hazards regression model will be formulated. The operational time scale will be calendar time, with March 30, 2022, taken to be time zero, with a second set of analyses using study time. Participants enter the risk set at D15 following their booster dose and are censored at D1 if they develop COVID-19 prior to D15. The hazard for the model for participant *i* is

$$\lambda_i\ (t) = \lambda_0\ (t) \exp\{\boldsymbol{X}_i^T \boldsymbol{\beta} + \gamma_1 \log_{10}(\widehat{Ab}_i(t - \tau_i^{D15}))\} I\{t > \tau_i^{D15}\},$$

where τ_i^{D15} is the calendar time of D15 post-booster for participant *i*, and $\boldsymbol{\beta}$ is a vector of parameters whose values correspond to log hazard ratios for baseline covariates, and $\widehat{Ab}_i(t - \tau_i^{D15})$ is the predicted antibody for participant *i* at time $(t - \tau_i^{D15})$ post-D15. The analyses adjust for the variables listed in Table 5, where study time analyses include adjustment for FOI score and calendar time analyses exclude adjustment for FOI score (because the calendar time scale already provides partial confounding control due to secular trends in force of infection). For each D15 marker, we assess whether the marker is an exposure-proximal CoR via a Wald test of the null that γ_1 is equal to 0 and plot the estimated hazard ratio over the range of observed titers.

6.6 Analysis of SARS-CoV-2 genotype-specific correlates of risk and protection

For each data analysis that studies lineage-specific or distance-specific correlates, if fewer than 10% of COVID-19 primary endpoint are missing sequence information, then simple complete-case analysis is done. For analyses that consider genotype as a lineage, GISAID single hard imputation will be used. For each COVID-19 event with a missing lineage, the modal GISAID data base lineage on the date of the COVID-19 onset event will be assigned.

Objectives peak gt 1.-2. are assessed with the Cox-model based methods treating the given lineage under consideration and all other lineages as two competing risks. The analysis is implemented as follows. We summarize how the marginalized Cox modeling approach is adapted when focusing on a specific COVID-19 genotype of interest (say J=1), where all other genotypes (pooled into a category J=2) are treated as a competing risk for J=1. For CoR analysis and an intermediate step of CVE analysis, now the parameter being estimated is the marginal conditional cumulative incidence function

risk1(tF, 1|s):= E_X[P(T <= tF, J=1|s, X, A=1)],

where T is the time until the first event of J=1 and J=2, tF is the final time point of interest, s is a fixed value of the marker, X are baseline covariates to adjust for, and A=1 indicates the vaccine arm or pooled set of vaccine arms included in a given data analysis. Now, risk1(t, 1|s) for given t can be written as

risk1(t, 1|s) = ò I(0 <= s <= t) lambda1(t', 1|s, x) S1(t'|s, x) dt' dF(x|s)

= {ò I(0 <= s <= t) lambda1(t', 1|s,x) S1(t'|s, x) dt' f1(s|x) dH(x)} / {ò f1(s|x)dH(x)}

where lambda1(t, 1|s, x) = lambda01(t|A=1) exp{beta1^T (s, x)} is the conditional cause-specific hazard function,[21] F(.|.) is the conditional distribution of X given S and A=1, f1(.|.) is the conditional density of S given X and A=1, H(.) is the distribution of baseline covariates X, and S1(t|s, x) = P(T>t|S=s, X=x, A=1).

Estimation of risk1(t, 1|s) could be done based on nonparametric kernel smoothing for estimation of lambda01(t|A=1). To avoid kernel smoothing (and bandwidth dependence), we will instead base estimation on the equation

risk1(t, 1|s) = ò risk_1(t, 1|s,x) f1(s|x)dH(x) / ò f1(s|x)dH(x).

Estimation of risk1(t, 1|s,x) can be done under the assumption of the Fine-Gray proportional subdistribution hazards model (implemented in the *cmprsk* R package available on CRAN); however, we prefer the interpretation of cause-specific hazards[21] to subdistribution hazards as in Fine and Gray[22] in a rare event situation such as in COVAIL. Therefore, we estimate risk1(t, 1|s, x) by modeling cause-specific hazard functions and combining them to get at the cumulative incidence function estimate (https://www.publichealth.columbia.edu/research/population-health-methods/competing-risk-analysis). Implementation of this approach can be found in the R packages *riskRegression*[23] and *kyotil::predictCompetingRisk2*, the latter of which offers additional support for a two-phase sampling design through inverse probability of sampling weights.

We then estimate risk1(t, 1|s) by G-computation

Est. risk1(t, 1|s) = (1/n1) $å_{i=1,…,n1}$ Est. risk1(t, 1|s, Xi).

As for the analyses without competing risks, the bootstrap is used for calculating 95% confidence intervals about the controlled risk curve r_C(1, s) = risk1(tF, 1|s).

Now, the controlled RR curve of interest for strain J=1 is

$RR_C^{\{J=1\}}$(s) = 1 – {P(T(1, s) <= tF, J(1, s)=1) / P(T(0) <= tF, J(0)=1)}

= 1 – {r_C(1, s) / P(T(0) <= tF, J(0)=1)}.

Under the causal identifiability assumptions this curve equals 1 – {risk1(tF, 1|s) / risk0(tF, 1)} where risk0(tF, 1) = E_X[P(T <= tF, J=1|X, A=0)]. The denominator risk0(tF, 1) is estimated by using the unbounded version of the targeted maximum likelihood estimator of Benkeser et al.,[11] implemented in the R package *survtmle*, and is implemented in the same way as described in Section 2.4.

7. Additional analysis notes

Accommodating missing nAb ID50 marker data at D29, D91, D181

If there are participants missing nAb ID50 marker data at D29, D91, and/or D181, imputation will be performed as follows. First, we will check if the missingness pattern is 'all-or-none' at a given time point, in terms of the full set of antigen-specific readouts being available or none of this set being available. Then he *mice* R package will be used to impute missing values according to the following plan, where 'at-risk' means no COVID-19 or asymptomatic infection event and not censored up to the time point under consideration:

- For imputing each D29 log10 ID50 missing value, perform predictive mean matching using the input variables the set of D15 log10 ID50 values against all antigens, vaccine booster arm, indicator of detectable D1 nAb ID50 (same antigen as the D29 value being imputed), age, sex assigned at birth, where the model is built based on the subset of ***Immunemarkerset***==1 participants still at risk at D29.
- For imputing each D91 log10 ID50 missing value, perform predictive mean matching using the input variables the set of D29 log10 ID50 values against all antigens, vaccine booster arm, indicator of detectable D1 nAb ID50 (same antigen as the D91 value being imputed), age, sex assigned at birth, where the model is built based on the subset of ***Immunemarkerset***==1 participants still at risk at D91.
- For imputing each D181 log10 ID50 missing value, perform predictive mean matching using the input variables the set of D91 log10 ID50 values against all antigens, vaccine booster arm, indicator of detectable D1 nAb ID50 (same antigen as the D181 value being imputed), age, sex

assigned at birth, where the model is built based on the subset of **Immunemarkerset**==1 participants still at risk at D181.

In reality, the data set has no such missingness, such that the imputations described above were not needed.

All p-values are 2-sided and no adjustments for multiple hypothesis testing are performed.

8. Metadata Required for Supporting the Data Analyses

The following metadata from the primary COVAIL data base are required for enabling the statistical analyses of immune correlates of protection, which is intended to include the data that were used in the Branche et al.[1,2] data analysis of COVAIL.

a. Information on randomized treatments and adherence to randomized treatments
   - Indicator of being enrolled
   - Treatment arm assignment
   - Dates of received vaccinations and treatment arms received on those dates (or dates re-coded as numbers of days from a reference date).
b. Information on participant retention and study visit attendance
   - Dates of last contact (or re-coded dates) that will be used to define right-censoring of failure times
   - Dates of all study visits (or re-coded dates)
c. Information on SARS-CoV-2 diagnostics
   - Dates of diagnostic tests and types of diagnostic tests administered
   - Results of diagnostic testing results, including information on whether a testing result is missing
   - These data are used to define SARS-CoV-2 infection study endpoints (occurrence and event times)
   - The "previous infection" variable used in previous COVAIL data analyses[1,2] that is defined based on anti-N antibody seropositivity at baseline and/or by self-reported past positive SARS-CoV-2 PCR or antigen testing.
d. Information on symptomatic COVID-19 events
   - Dates of a symptomatic COVID-19 failure event that will be used to define failure event times
   - Available information on COVID-19 symptoms with dates of occurrence of those symptoms
   - Viral load (copies/ml) from available SARS-CoV-2 NAAT/PCR testing values, including on whether a test was administered but the viral load is missing. The dates of viral load testing results should be clearly aligned with symptomatic COVID-19 failure event times.
   - Indicator of whether a lineage is available
   - Indicator of whether a SARS-CoV-2 sequence is available
   - SARS-CoV-2 lineage and SARS-CoV-2 amino acid sequence
   - Sequences of all of the vaccine-insert strains for the 17 vaccine booster arms
e. Information on baseline demographics at enrollment
   - Age, sex assigned at birth, race, ethnicity, geographic location information

f. Information on baseline demographics that are used in the modeling building a COVID-19 risk score
   - Additional variables collected at enrollment that potentially indicate elevated exposure to SARS-CoV-2 (e.g., health care worker status, CDC ILI region)
   - Additional variables collected at enrollment that potentially indicate elevated risk of COVID-19 (e.g., known co-morbidity prognostic variables)

g. Information on sampling and measurement of immunological variables
   - For each visit date with planned sera collection, an indicator of whether a sample was collected
   - For each visit date with planned sera collection and application of an immunological assay to the sera, an indicator of whether an assay value is available. Indicator variables are included for each type of immunological assay applied to sera.
   - For each visit date with planned PBMC collection, an indicator of whether a sample was collected
   - For each visit date with planned PBMC collection and application of an immunological assay to the PBMC, an indicator of whether an assay value is available. Indicator variables are included for each type of immunological assay applied to sera.
   - Indicator of whether a participant was sampled for the 25% subset of each study arm for measurement of ID50 titer against Omicron BA.2.12.1.

h. Information on values of immunological variable readouts
   - For each visit date with planned sera or PBMC collection and application of an immunological assay to the sample, the value of an assay readout, with a missing data code if not available. The value may be < LOD or < LLOQ if an LOD or LLOQ is used, or > ULOQ if a ULOQ is used, although there is a preference for including actual readouts > ULOQ.
   - For each assay type that is used and each assay limit that is used for an assay (e.g., LOD, LLOQ, ULOQ, variables indicating the values of the limits

9. Construction of Derived Variables for Data Analysis Based on the COVAIL Primary Data Set Received from Emmes 12-11-23

In the following description of derived variables, variable names from the original data set are placed in italics.

Treatment variables.

All of the following treatment variables are based on *treatment_actual* or *stage*.

TrtmRNA: Indicator a participant is in one of the mRNA vaccine groups (Arms 1-12, 16-17). TrtmRNA has no missing values.

TrtonedosemRNA: Indicator a participant is in one of the one-dose mRNA vaccine groups (Arms 1-2, 4-12, 16-17). TrtonedosemRNA has no missing values.

TrtA: For participants defined by [Moderna vaccines (arms 1-2, 5-6 pooled) vs. Pfizer-BioNTech vaccines (arms 7-9, 12 pooled)], indicator of whether a participant is in the Moderna vaccine group. TrtA is NA if a participant is in neither group.

TrtB: For participants defined by [mRNA Prototype vs. mRNA Omicron-Containing] Prototype vaccines (arms 1, 7 pooled) vs. Omicron-containing vaccines (2, 4-6, 8, 9, 12, 16, 17 pooled), indicator of whether a participant is in the mRNA Prototype group. TrtB is NA if a participant is in neither group.

TrtC: For participants defined by [mRNA Bivalent vs. mRNA Monovalent] Bivalent Omicron-containing vaccines (arms 2, 4, 6, 8, 12, 16, 17 pooled) vs. Monovalent Omicron-containing vaccines (arms 5, 9 pooled), indicator of whether a participant is in the mRNA Bivalent group. TrtC is NA if a participant is in neither group.

TrtA==1 is coded as *treatment_actual* equal to any of the 5 values (Arms 1-2, 5-6 respectively) "1 Dose Prototype (Moderna)", "1 Dose Beta + Omicron (Moderna)", "1 Dose Omicron (Moderna)", "1 Dose Omicron + Prototype (Moderna)"

TrtA==0 is coded as *treatment_actual* equal to any of the 4 values (Arms 7-9, 12 respectively) "Wildtype/Prototype (Pfizer-BioNTech 1)", "Beta + Omicron (Pfizer-BioNTech 1)", "Omicron (Pfizer-BioNTech 1)", "Omicron + Wildtype/Prototype (Pfizer-BioNTech 1)"

TrtB==1 is coded as *treatment_actual* equal to either of the 2 values (Arms 1, 7 respectively) "1 Dose Prototype (Moderna)", "Wildtype/Prototype (Pfizer-BioNTech 1)"

TrtB==0 is coded as *treatment_actual* equal to any of the values (Arms 2, 4-6, 8, 9, 12, 16, 17 respectively) "1 Dose Beta + Omicron (Moderna)", "1 Dose Delta + Omicron (Moderna)", "1 Dose Omicron (Moderna)", "1 Dose Omicron + Prototype (Moderna)", "Beta + Omicron (Pfizer-BioNTech 1)", "Omicron (Pfizer-BioNTech 1)", "Omicron + Wildtype/Prototype (Pfizer-BioNTech 1)", "Omicron BA.1 + Wildtype/Prototype (Pfizer-BioNTech 2)", "Omicron BA.4/BA.5 + Wildtype/Prototype (Pfizer-BioNTech 2)"

TrtC==1 is coded as *treatment_actual* equal to any of the values (Arms 2, 4, 6, 8, 12, 16, 17 respectively) "1 Dose Beta + Omicron (Moderna)", "1 Dose Delta + Omicron (Moderna)", "1 Dose Omicron + Prototype (Moderna)", "Omicron (Pfizer-BioNTech 1)", "Omicron + Wildtype/Prototype (Pfizer-BioNTech 1)", "Omicron BA.1 + Wildtype/Prototype (Pfizer-BioNTech 2)", "Omicron BA.4/BA.5 + Wildtype/Prototype (Pfizer-BioNTech 2)"

TrtC==0 is coded as *treatment_actual* equal to either of the values (Arms 5, 9 respectively) "1 Dose Omicron (Moderna)", "Omicron (Pfizer-BioNTech 1)"

TrtmRNA==1 is coded as *stage* equal to 1, 2, or 4

TrtmRNA==0 is coded as *stage* equal to 3.

TrtonedosemRNA==1 is coded as *stage* equal to 1, 2, or 4 and *treatment_actual* not equal to "2 Dose Beta + Omicron (Moderna)"

TrtonedosemRNA==0 is coded as Else (TrtonedosemRNA not equal to 1)

Eligibility for inclusion in immunogenicity and immune correlates analyses variables.

Immunemarkerset: Indicator of whether a participant is included in immunogenicity and immune correlates analyses. This indicator is a requirement for all immunogenicity analyses, Day 15 marker correlates analyses, and exposure-proximal correlates analyses. While this indicator is independent of vaccine arm, note that all analyses restrict to mRNA vaccine arms except multivariable superlearning CoR that includes all 16 one-dose vaccine arms. Immunemarkerset==1 requires all the following conditions:

- No eligibility deviation (eligibility_deviation==FALSE)
- nAb ID50 data measured against D614G at the D1 and D15 visits
  (having both the first 2 conditions is coded as perprotocol==1 in the derived variables in Section 10)
- No COVID-19 endpoint after booster receipt and by 6 days post D15 visit (as coded by EarlyendpointD15==1)

Note: All immunogenicity and immune correlates analyses are done in the cohort with Immunemarkerset==1

ph1.D15: an alias for Immunemarkerset with the added condition that treatment arm!=3

ImmunemarkersetD92toD181: Indicator of whether a participant is included in immune correlates analyses for the time-period D15_92to188 for capturing COVID-19 endpoints. The subset of participants with a COVID-19 event >= 7 days post D15 visit and up to 91 days post D15 visit are excluded from the analysis, such that ImmunemarkersetD92toD181 is the same as Immunemarkerset except this subset of participants has ImmunemarkersetD92toD181==0 & Immunemarkerset==1.

ph1.D92: an alias for ImmunemarkersetD92toD181 with the added condition that treatment arm!=3

COVID-19 endpoint failure time variables.

COVIDtimeD22toD91: Minimum of the number of days from the D15 visit to the first COVID-19 endpoint or right-censoring, whichever occurs first. The first COVID-19 endpoint must occur >= 7 days post D15 visit through to 91 days post D15 visit. Right-censoring is defined by the first event of (1) reaching 91 days post D15 visit without a COVID-19 event, (2) receipt of a second dose [applicable for study arm 3 only, *treatment_actual*=="2 Dose Beta + Omicron (Moderna)"], (3) early termination, (4) receiving an out-of-study boost, and (5) the data cut date of 2023-07-13.

COVIDIndD22toD91: Indicator of whether COVIDtimeD22toD91 represents a COVID-19 endpoint (>= 7 days post D15 visit through to 91 days post D15 visit).

COVIDtimeD92toD181: Minimum of the number of days from the D15 visit to the first COVID-19 endpoint or right-censoring, whichever occurs first. The first COVID-19 endpoint must occur >= 92 days post D15 visit through to 188 days post D15 visit. Right-censoring is defined by the first event of (1)

reaching 188 days post D15 visit without a COVID-19 event, (2) receipt of a second dose (applicable for study arm 3 only), (3) early termination, (4) receiving an out-of-study boost, and (5) the data cut date of 2023-07-13. Participants with a COVID-19 event >= 7 days post D15 visit and up to 91 days post D15 visit are excluded from the analysis.

COVIDIndD92toD181: Indicator of whether COVIDtimeD22toD91 represents a COVID-19 endpoint (>= 92 days post D15 visit through to 188 days post D15 visit). Analyses that use (COVIDtimeD92toD181, COVIDIndD92toD181) restrict to ImmunemarkersetD92toD181==1.

COVIDtimeD22toD181 (aka COVIDtime_7to188): Minimum of the number of days from the D15 visit to the first COVID-19 endpoint or right-censoring, whichever occurs first. The first COVID-19 endpoint must occur >= 7 days post D15 visit through to 188 days post D15 visit. Right-censoring is defined by the first event of (1) reaching 188 days post D15 visit without a COVID-19 event, (2) receipt of a second dose (applicable for study arm 3 only), (3) early termination, (4) receiving an out-of-study boost, and (5) the data cut date of 2023-07-13.

COVIDIndD22toD181: Indicator of whether COVIDtimeD22toD181 represents a COVID-19 endpoint (>= 7 days post D15 visit through to 188 days post D15 visit). The following identity holds: COVIDIndD22toD181== (COVIDIndD22toD91 | COVIDIndD92toD181).

COVIDtimeD22toend: Minimum of the number of days from the D15 visit to the first COVID-19 endpoint or right-censoring, whichever occurs first. The first COVID-19 endpoint must occur >= 7 days post D15 visit through to the end of follow-up. Right-censoring is defined by the first event of (1) date of last contact, (2) receipt of a second dose (applicable for study arm 3 only), (3) early termination, (4) receiving an out-of-study boost, and (5) the data cut date of 2023-07-13. This variable is not analyzed according to the SAP, but it is included for a final data set.

COVIDIndD22toend: Indicator of whether COVIDtimeD22toend represents a COVID-19 endpoint (>= 7 days post D15 through to the end of follow-up).

SARS-CoV-2 lineage/sequence variables for COVID-19 endpoints.

COVIDlineage: The lineage causing a COVID-19 endpoint, which is the observed lineage (variable *TRUNCLIN_SWAB1*) if available and is the PANGOLIN-imputed lineage if not available. All COVID endpoints have an observed value for COVIDlineage.

COVIDlineageObserved: Indicator of whether the original variable *TRUNCLIN_SWAB1* had an observed value.

Baseline risk score variable.

Baselineriskscore: Calculated as described in Section 6.1.

FOI score variables.

FOIoriginal: Calculated as described in Section 6.1, the variable before standardizing to have mean 0 and standard deviation 1, for descriptive plotting purposes.

FOIstandardized: Calculated as described in Section 6.1, which is the variable F that is adjusted for in most immune correlates analyses.

Neutralization titer ID50 variables post dose one for the antigens measured by Monogram D614G, Delta, Beta, Omicron BA.1, and Omicron BA.4/BA.5 *(all arms but arm 3).*

Bpseudoneutid50_D614G

Bpseudoneutid50_Delta

Bpseudoneutid50_Beta

Bpseudoneutid50_BA.1

Bpseudoneutid50_BA.4.BA.5

Bpseudoneutid50_MDW

Day15pseudoneutid50_D614G

Day15pseudoneutid50_Delta

Day15pseudoneutid50_Beta

Day15pseudoneutid50_BA.1

Day15pseudoneutid50_BA.4.5

Day15pseudoneutid50_MDW

Day29pseudoneutid50_D614G

Day29pseudoneutid50_Delta

Day29pseudoneutid50_Beta

Day29pseudoneutid50_BA.1

Day29pseudoneutid50_BA.4.BA.5

Day29pseudoneutid50_MDW

Day91pseudoneutid50_D614G

Day91pseudoneutid50_Delta

Day91pseudoneutid50_Beta

Day91pseudoneutid50_BA.1

Day91pseudoneutid50_BA.4.BA.5

Day91pseudoneutid50_MDW

Neutralization ID50 titer variables for the 2-dose arm 3 (not analyzed in this SAP).

The Baseline (B) variables are the same as for the other arms. The post baseline variables are post-dose 2, at 14, 28, and 91 days post-dose 2 and labeled as Day 71, Day 85 and Day 147, respectively.

Bpseudoneutid50_D614G: log10 nAb ID50 titer against D614G (other variables below similar)

Bpseudoneutid50_Delta

Bpseudoneutid50_Beta

Bpseudoneutid50_BA.1

Bpseudoneutid50_BA.4.BA.5

Bpseudoneutid50_MDW

Day71pseudoneutid50_D614G

Day71pseudoneutid50_Delta

Day71pseudoneutid50_Beta

Day71pseudoneutid50_BA.1

Day71pseudoneutid50_BA.4.BA.5

Day71pseudoneutid50_MDW

Day85pseudoneutid50_D614G

Day85pseudoneutid50_Delta

Day85pseudoneutid50_Beta

Day85pseudoneutid50_BA.1

Day85pseudoneutid50_BA.4.BA.5

Day85pseudoneutid50_MDW

Day147pseudoneutid50_D614G

Day147pseudoneutid50_Delta

Day147pseudoneutid50_Beta

Day147pseudoneutid50_BA.1

Day147pseudoneutid50_BA.4.BA.5

Day147pseudoneutid50_MDW

NumberdaysD15toD29: Number of days between the D15 visit and the D29 visit

Notes aiding calculation of derived variables.

- The Baseline (D1) visit date is coded by *primary_vax1_date*
- The first COVID-19 event failure time information is based on *infect_date1* and *symptomatic_infect1*
- The anti-N serotesting data for determining asymptomatic infection is based on *NAntibody*
- Pseudovirus nAb ID50 titer data is derived from *ASSAY=="PsV ID50", RESULT, RESULT_limit*
- The D15 neutralization variables have missing values for BA4BA5. These are imputed based on a linear model with 2 input variables D29 log10 nAb ID50 against BA4BA5 and the number of days between D15 and D29 (variable NumberdaysD15toD29). As noted at the end of Section 1, a small number of participants are missing a D29 BA.4/BA.5 nAb ID50 value, for which this imputation approach will not work. However, these participants have an observed D15 BA.1 nAb ID50 value. For this small subset, the imputation of D15 BA.4/BA.5 nAb titer will be done based on a linear regression with sole predictor D15 log10 nAb ID50 against BA.1. These imputations are only done for arms other than Arm 3.
- The set of ptids included in immunogenicity and immune correlates analyses requires the following condition: Immunemarkerset==1, equivalently coded as ph1.D15==1
  The derived data set uses the variable *naive* to code for *Infstat* (the variable name in the data set received from Emmes).

10. Changes to the original Statistical Analysis Plan (12-22-2023 version)

10.1 Changes to the original Statistical Analysis Plan (12-22-2023 version) That are Valid Based on Not Being Influenced by Results

1. Section 2.4 of the SAP specified use of the Benkeser et al.[11] method implemented in the R package *survtmle* available at CRAN to study overall cumulative incidence curves and ratios of curves. However, upon data analysis it was discovered that the *survtmle* package on CRAN had bugs that could not be repaired by the Interim Report deliverable date January 24, 2023. Therefore, the analysis was done with a similar method and R package: Westling et al.[24] implemented in the R packages *CFsurvival* (https://github.com/tedwestling/CFsurvival) and *survSuperLearner* (https://github.com/tedwestling/ survSuperLearner). This decision was made based only on technical ability of the *survtmle* R package, a decision made free from influence of any results.

2. On 1-10-24 it was discovered that treatment grouping 1A had been defined as Arms 1-2, 4-6; yet Arm 4 should have been excluded because 1B does not have a counterpart Delta + Omicron insert vaccine. Therefore, the SAP was updated to re-define grouping 1A as Arms 1-2, 5-6. This decision was the intended design, free from influence of results.

3. 1-29-24: Added implementation of Obj. peak 1., Obj. peak 7., and Obj. exp-prox. 1. for the Stage 3 vaccine arms (pooled), where Obj. peak 1. and Obj. peak 7. are only implemented for the D15_7to188

period for capturing COVID-19 endpoints. These additions are described at the end of Sections 3.2 and 4.

4. 1-29-24: For the 13 one-dose mRNA vaccine arms pooled, text was added in Section 6.3 elaborating that for Obj. peak 2. and Obj. exp-prox 2. that assess whether the D15 nAb titer correlate of risk is modified by Naïve/Non-Naïve status includes separate correlates analyses in the Naïve and Non-naïve subgroups if interaction tests show evidence of interaction. For both of these interaction objectives, there was significant evidence of interaction for the D15_7to91 and D15_7to188 periods for capturing COVID-19 endpoints, but not for the D15_92to188 period, implying that the subgroup analyses are warranted for the D15_7to91 and D15_7to188 periods. Similarly, in the same section text was added about adding subgroup analyses based on D1 nAb titer if Obj. peak 3. / Obj. exp-prox 3. show evidence of interaction of D1 and D15 nAb titer in their association with COVID-19.
5. 2-16-24: In Section 6.5, added the sentence "Hypothesis tests for goodness of proportional hazards model fit will be conducted using the Grambsch and Therneau[20] method as implemented in the R function *cox.zph* in the *survival* library."
6. 3-12-24: Throughout the SAP, clarified the three time periods for capturing COVID-19 endpoints that were analyzed, and updating the notation to be clearer. In particular, language in Section 2.2 was updated as follows:

   There are three groups of COVID-19 cases that are considered in data analyses: Cases occurring >= 7 days after the D15 visit and diagnosed with COVID-19 by 188 days post D15 visit ("D15_7to188 Cases"), Cases occurring >= 7 days after the D15 visit and diagnosed with COVID-19 by 91 days post D15 visit ("D15_7to91 Cases"), and cases occurring between 92 days post D15 visit and diagnosed with COVID-19 by 188 days post D15 visit ("D15_92to188 Cases").

10.2 Post-hoc exploratory analyses after the original Statistical Analysis Plan (12-22-23) specification

1-6-24: Repeated the overall COVID-19 cumulative incidence comparison analyses specified in Section 2.4 in vaccine arms defined by vaccine-insert to enhance understanding of the results. Conducted a sensitivity analysis adding D1 nAb ID50 titer against BA.1 to the set of covariates adjusted for.

1-10-24: The exploratory finding that the prototype vs. omicron insert vaccine effect is present for Pfizer-BioNTech and absent for Moderna generates interest in assessing a correlate of protection for the Stage 2 Pfizer-BioNTech prototype vs. Stage 2 Pfizer-BioNTech Omicron. Controlled relative vaccine efficacy analyses for the Stage 2 Pfizer-BioNTech arms (all of which are randomized) are conducted based on this finding.

2-16-24: At the end of Section 6.3 added the following text:
"Exploratory analyses may be conducted that assess D1, D15 nAb titer interactions separately in the Naïve and Non-naïve subgroups, as the correlation of Naïve status with D1 titer poses challenges to the simultaneous study of how Naïve status and D1 titer may modify the association of D15 nAb titer with COVID-19. Similarly, exploratory analyses may be conducted that assess D1, time-varying nAb titer interactions separately in the Naïve and Non-naïve subgroups, as the correlation of Naïve status with time-varying titer poses challenges to the simultaneous study of how Naïve status and D1 titer may modify the association of time-varying nAb titer with COVID-19."

2-16-24: Added an exploratory analysis to estimate hazard ratios for subgroups of the 13 one-dose mRNA participants in the 9 categories defined by (D1 nAb titer tertiles x D15 nab titer tertiles). The analyses are considered both overall and for the Naïve subgroup, to aid intepretability.

10.3 Changes made in response to reviewer comments

The following changes were made to the exposure-proximal correlates of risk analyses in response to reviewer comments:

- Left censoring of antibody values below the limit of detection was handled using mixed effects tobit models for the antibody decay models, instead of using imputation with ½ the limit of detection.
- The linear mixed effects antibody decay models were replaced with piecewise linear mixed effects antibody decay models with a bend at 76 days after day 15 due to improved model fit for many of the markers/arms.
- Confidence intervals and p-values for hazard ratios estimated in the exposure-proximal analyses were determined based on the bootstrap procedure described in the Supplementary Material in order to account for uncertainty in the predicted titers.

Additional peak correlates analyses restricted to female and male participants in the baseline naive participants were conducted in response to editor's comments.

The following sensitivity analyses were conducted in response to editors' comments:

1. Additional covariate adjustment strategies including (1) adjusting for an age ≥ 65 indicator and the FOI score in the peak correlates analyses; (2) not adjusting for any covariate in the peak and exposure-proximal analyses.
2. Additional covariate adjustment strategies (as above) when conducting the interaction test.
3. Analyses restricted to the COVE endpoints.

References

1. Branche AR, Rouphael NG, Diemert DJ, et al. Comparison of bivalent and monovalent SARS-CoV-2 variant vaccines: the phase 2 randomized open-label COVAIL trial. *Nat Med* 2023; **29**(9): 2334-46.
2. Branche AR, Rouphael NG, Losada C, et al. Immunogenicity of the BA.1 and BA.4/BA.5 Severe Acute Respiratory Syndrome Coronavirus 2 Bivalent Boosts: Preliminary Results From the COVAIL Randomized Clinical Trial. *Clin Infect Dis* 2023; **77**(4): 560-4.
3. Gilbert PB, Montefiori DC, McDermott AB, et al. Immune correlates analysis of the mRNA-1273 COVID-19 vaccine efficacy clinical trial. *Science* 2022; **375**(6576): 43-50.
4. Fong Y, Huang Y, Benkeser D, et al. Immune Correlates Analysis of the PREVENT-19 COVID-19 Vaccine Efficacy Clinical Trial *Nature Communications* 2023; **14**(1): 331.
5. Fong Y, McDermott AB, Benkeser D, et al. Immune Correlates Analysis of the ENSEMBLE Single Ad26.COV2.S Dose Vaccine Efficacy Clinical Trial. *Nature Microbiology* 2022; **7**(12): 1996-2010.
6. Benkeser D, Fong Y, Janes HE, et al. Immune correlates analysis of a phase 3 trial of the AZD1222 (ChAdOx1 nCoV-19) vaccine. *NPJ Vaccines* 2023; **8**(1): 36.

7. Benkeser D, Montefiori DC, McDermott AB, et al. Comparing antibody assays as correlates of protection against COVID-19 in the COVE mRNA-1273 vaccine efficacy trial *Science Translational Medicine* 2023; **15**(692): eade9078.
8. Fong Y, Dang L, Zhang B, et al. Neutralizing Antibody Immune Correlates for a Recombinant Protein Vaccine in the COVAIL Trial (Submitted). 2024.
9. He Z, Fong Y. Maximum diversity weighting for biomarkers with application in HIV-1 vaccine studies. *Stat Med* 2019; **38**(20): 3936-46.
10. Newcombe RG. Interval estimation for the difference between independent proportions: comparison of eleven methods. *Statistics in medicine* 1998; **17**(8): 873-90.
11. Benkeser D, Gilbert PB, Carone M. Estimating and Testing Vaccine Sieve Effects Using Machine Learning. *J Am Stat Assoc* 2019; **114**(527): 1038-49.
12. Price BL, Gilbert PB, van der Laan MJ. Estimation of the optimal surrogate based on a randomized trial. *Biometrics* 2018; **74**(4): 1271-81.
13. Gilbert PB, Fong Y, Kenny A, Carone M. A Controlled Effects Approach to Assessing Immune Correlates of Protection. *Biostatistics* 2023; **24**(4): 850–65.
14. Hubbard AE, Kherad-Pajouh S, van der Laan MJ. Statistical Inference for Data Adaptive Target Parameters. *Int J Biostat* 2016; **12**(1): 3-19.
15. Williamson BD, Gilbert PB, Simon NR, Carone M. A General Framework for Inference on Algorithm-Agnostic Variable Importance. *Journal of the American Statistical Association* 2023; **118**(543): 1645-58.
16. Neidich SD, Fong Y, Li SS, et al. Antibody Fc effector functions and IgG3 associate with decreased HIV-1 risk. *J Clin Invest* 2019; **129**(11): 4838-49.
17. van der Laan L, Zhang W, Gilbert PB. Nonparametric estimation of the causal effect of a stochastic threshold-based intervention. *Biometrics* 2023; **79**(2): 1014-28.
18. Prentice RL. Surrogate endpoints in clinical trials: definition and operational criteria. *Stat Med* 1989; **8**(4): 431-40.
19. Benkeser D, Díaz I, Ran J. Inference for natural mediation effects under case-cohort sampling with applications in identifying COVID-19 vaccine correlates of protection. arXiv:2103.02643 [stat.ME] Access date: 29 Apr 2022. 2021.
20. Grambsch PM, Therneau TM. Proportional hazards tests and diagnostics based on weighted residuals. *Biometrika* 1994; **81**(3): 515-26.
21. Prentice RL, Kalbfleisch JD, Peterson AV, Jr., Flournoy N, Farewell VT, Breslow NE. The analysis of failure times in the presence of competing risks. *Biometrics* 1978; **34**(4): 541-54.
22. Fine JP, Gray RJ. A proportional hazards model for the subdistribution of a competing risk. *Journal of the American statistical association* 1999; **94**(446): 496-509.
23. Ozenne B, Sørensen AL, Scheike T, Torp-Pedersen C, Gerds TA. riskRegression: predicting the risk of an event using Cox regression models. *The R Journal* 2017; **9**(2): 440-60.
24. Westling T, Luedtke A, Gilbert PB, Carone M. Inference for treatment-specific survival curves using machine learning. *Journal of the American Statistical Association* 2023; **119**(546): 1541-53.
