# Supplementary material for: Neutralizing antibody immune correlates in COVAIL trial recipients of an mRNA second COVID-19 vaccine boost
Source: Nat Commun. 2025 Jan 17;16:759. doi: 10.1038/s41467-025-55931-w (PMC11748719; doi:10.1038/s41467-025-55931-w)
Supplement: Supplementary file 3 — Description of Additional Supplementary Files [file 41467_2025_55931_MOESM3_ESM.pdf]

Description of Additional Supplementary Files

File Name: Supplementary Software 1
Description: R code for conducting the cumulative incidence analysis, controlled risk curve analysis, controlled relative vaccine efficacy analysis, and all exposure-proximal correlates of risk analyses.
