# Supplementary material for: Neutralizing antibody immune correlates in COVAIL trial recipients of an mRNA second COVID-19 vaccine boost
Source: Nat Commun. 2025 Jan 17;16:759. doi: 10.1038/s41467-025-55931-w (PMC11748719; doi:10.1038/s41467-025-55931-w)
Supplement: Supplementary file 5 — Reporting Summary [file 41467_2025_55931_MOESM5_ESM.pdf]

nature portfolio

Corresponding author(s): Dean Follmann

Last updated by author(s): Jan 3, 2025

Reporting Summary

Nature Portfolio wishes to improve the reproducibility of the work that we publish. This form provides structure for consistency and transparency in reporting. For further information on Nature Portfolio policies, see our Editorial Policies and the Editorial Policy Checklist.

Statistics

For all statistical analyses, confirm that the following items are present in the figure legend, table legend, main text, or Methods section.

n/a	Confirmed	
☐	☒	The exact sample size (n) for each experimental group/condition, given as a discrete number and unit of measurement
☐	☒	A statement on whether measurements were taken from distinct samples or whether the same sample was measured repeatedly
☐	☒	The statistical test(s) used AND whether they are one- or two-sided<br>*Only common tests should be described solely by name; describe more complex techniques in the Methods section.*
☐	☒	A description of all covariates tested
☒	☐	A description of any assumptions or corrections, such as tests of normality and adjustment for multiple comparisons
☐	☒	A full description of the statistical parameters including central tendency (e.g. means) or other basic estimates (e.g. regression coefficient) AND variation (e.g. standard deviation) or associated estimates of uncertainty (e.g. confidence intervals)
☐	☒	For null hypothesis testing, the test statistic (e.g. F, t, r) with confidence intervals, effect sizes, degrees of freedom and P value noted<br>*Give P values as exact values whenever suitable.*
☒	☐	For Bayesian analysis, information on the choice of priors and Markov chain Monte Carlo settings
☒	☐	For hierarchical and complex designs, identification of the appropriate level for tests and full reporting of outcomes
☐	☒	Estimates of effect sizes (e.g. Cohen's d, Pearson's r), indicating how they were calculated

Our web collection on statistics for biologists contains articles on many of the points above.

Software and code

Policy information about availability of computer code

Data collection	Luminescence, measured in relative light units, was measured using a Luminoskan luminometer.

Data analysis

For the neutralizing antibody assay, data analysis (inhibition curve fitting and ID50 determinations) was done using Monogram proprietary analysis software.
All analyses were done reproducibly on the basis of publicly available R scripts hosted on the GitHub collaborative programming platform (https://github.com/CoVPN/correlates_reporting2) as well as in Supplementary Software 1. The analyses described in the correlates_reporting2 module (Cox proportional hazards modeling of risk) were conducted in R (version 4.0.4) and used over 100 R packages (https://github.com/CoVPN/correlates_reporting2/blob/master/renv.lock). The analyses described in the "README.txt" file (cumulative incidence analysis, controlled risk curve analysis, and controlled relative vaccine efficacy analysis) were conducted in R (version 4.2.1) and used the R packages vaccine (version 1.2.1) and CFsurvival (version 0.1.0). The analyses described in the "README.md" file (all exposure-proximal correlates of risk analyses) were conducted in R (version 4.3.1) and used nearly 100 R packages, listed in the README.md file.
Code references:
52 Fong, Y. et al. Neutralizing antibody immune correlates in COVAIL trial recipients of an mRNA second COVID-19 vaccine boost. Zenodo. doi: 10.5281/zenodo.14324535. (2024).
53 R Core Team. R: A Language and Environment for Statistical Computing. R Foundation for Statistical Computing; Vienna, Austria. https://www.R-project.org. (2023).
54 Kenny, A. vaccine: Statistical Tools for Immune Correlates Analysis of Vaccine Clinical Trial Data. R package version 1.2.1. https://CRAN.R-project.org/package=vaccine. (2024).
55 Westling, T. CFsurvival. R package for nonparametric estimation of counterfactual survival functions and survival contrasts from right-censored data with an observational baseline treatment. https://github.com/tedwestling/CFsurvival/.

For manuscripts utilizing custom algorithms or software that are central to the research but not yet described in published literature, software must be made available to editors and reviewers. We strongly encourage code deposition in a community repository (e.g. GitHub). See the Nature Portfolio guidelines for submitting code & software for further information.

Data

Policy information about availability of data

All manuscripts must include a data availability statement. This statement should provide the following information, where applicable:

- Accession codes, unique identifiers, or web links for publicly available datasets
- A description of any restrictions on data availability
- For clinical datasets or third party data, please ensure that the statement adheres to our policy

The trial dataset will be available to appropriate academic parties on request from the corresponding author, in accordance with the data sharing policies of the Division of Microbiology and Infectious Diseases of NIAID with input from the investigator group subject to submission of a suitable study protocol and analysis plan. Requests should be directed to the corresponding author, Dr. Dean Follmann (dfollmann@niaid.nih.gov), and will be responded to within a month. Source data are provided with this paper.

Research involving human participants, their data, or biological material

Policy information about studies with human participants or human data. See also policy information about sex, gender (identity/presentation), and sexual orientation and race, ethnicity and racism.

Reporting on sex and gender

Cell lines: The pseudovirus neutralization assay used the HEK 293 cell line. The HEK 293 cell line was derived from a human fetus, with Lin et al. (https://doi.org/10.1038/ncomms5767) having reported evidence that HEK 293 cells are of female provenance (i.e. the complete absence of any Y-chromosome-derived sequence in high-coverage genomic sequencing data). It is unknown whether alternative neutralization assays would provide different results if based on a HEK cell line derived from a male donor.
In the COVAIL trial, sex reporting was based on sex assigned at birth and collected by participant self report.
Sex assigned at birth (male/female) was among the baseline demographic input variables used to build the risk score [see section 6.1 in the Statistical Analysis Plan (SAP), included in the supplementary material].
Of the 1006 participants eligible for cumulative incidence analyses, 648 (64.4%) were naïve (N=341 female, mean age 50.4 years and N=307 male, mean age 51.8 years). The remaining 358 (35.6%) were non-naïve (N=201 female, mean age 40.4 years and 157 male, mean age 41.2 years). Full demographic (including age and sex) and clinical information for all enrolled participants, naïve participants, and non-naïve participants is presented by stage and boost arm in Supplementary Tables 1-3.
Supplementary Figure 7 presents violin box plots of BA.1 ID50 titers at Day 1 (booster visit) and at Day 15, shown by non-cases and COVID-19 cases and stratified by booster-proximal cases, booster-distal-cases, and proximal+distal cases, presented separately for SARS-CoV-2 naïve and non-naïve participants, as well as presented separately for males and for females. Data points are shown for the following numbers of participants: A) Naïve male: 214 non-cases; 62 booster-proximal cases, 21 booster-distal cases, 83 total cases; Naïve female: 234 non-cases; 60 booster-proximal cases, 38 booster-distal cases, 98 total cases; Non-Naïve male: 147 non-cases; 7 booster-proximal cases, 3 booster-distal cases, 10 total cases; Non-Naïve female: 177 non-cases; 15 booster-proximal cases, 7 booster-distal cases, 22 total cases. B) A) Naïve male: 214 non-cases; 62 booster-proximal cases, 21 booster-distal cases, 83 total cases; Naïve female: 234 non-cases; 60 booster-proximal cases, 38 booster-distal cases, 98 total cases; Non-Naïve male: 147 non-cases; 7 booster-proximal cases, 3 booster-distal cases, 10 total cases; Non-Naïve female: 177 non-cases; 15 booster-proximal cases, 7 booster-distal cases, 22 total cases.
Supplementary Figure 18 presents the results of a post hoc analysis that estimated Cox model covariate-adjusted hazard ratios of COVID-19 per 10-fold increase in Day 15 ID50 titer for each marker defined by antigen BA.1, Beta, Delta, BA.4/BA.5, D614G, or weighted average, in SARS-CoV-2 naïve participants, separately among males and among females. It also presents the results of a post hoc analysis that estimated Cox model covariate-adjusted hazard ratios of COVID-19 per 10-fold increase in D15 BA.1 titer in designated subgroups of SARS-CoV-2 naïve participants, separately among males and among females (p-values are not included for this analysis because it was not pre-specified in the Statistical Analysis Plan).
Due to the lower numbers of COVID-19 cases with antibody data in non-naïve participants (N=22 booster-proximal, 10 booster-distal) compared to in naïve participants (N=122 booster-proximal, 59 booster-distal), post hoc sex-based Cox

	modeling analyses were not performed in non-naïve participants, as the case numbers would likely have been too low to enable meaningful conclusions.
Reporting on race, ethnicity, or other socially relevant groupings	Relevant terms for race and ethnicity were provided by the researchers, and participants could select mutiple choices of boxes to tick. Participants were classified into the different categories by self report.<br>Race and ethnicity were each included among the baseline demographic variables that were included for building the risk score (see section 6.1 in the SAP).
Population characteristics	Supplementary Tables 1 to 3 provide demographic and clinical information for all study participants eligible for cumulative incidence analyses (1), baseline SARS-CoV-2 naive study participants eligible for cumulative incidence analyses (2), and baseline SARS-CoV-2 non-naive study participants eligible for cumulative incidence analyses (3).
Recruitment	As described in Branche et al. Nat Med 2023, participants were recruited from 22 geographically diverse sites in the US with a goal of ensuring at least 25% of the total cohort would be non-white race or Hispanic ethnicity. Sites independently created a recruitment plan which may have included but were not limited to social media strategies, print media ads, radio and television ads, institutional news letters and the use of database registries. These specific methods were not tracked centrally by the sponsor but overseen by the central IRB. There may be inherent biases related to site specific recruitment strategies which might impact which participants were enrolled that could not be accounted for within the scope of the study.
Ethics oversight	The trial (NCT05289037) was reviewed and initially approved by the Advarra central institutional review board on March 22, 2022 and overseen by an independent Data and Safety Monitoring Board. All participants provided written informed consent before enrollment. A stipend was provided for participation in the study, which was determined by each enrolling site.

Note that full information on the approval of the study protocol must also be provided in the manuscript.

Field-specific reporting

Please select the one below that is the best fit for your research. If you are not sure, read the appropriate sections before making your selection.

☒ Life sciences ☐ Behavioural & social sciences ☐ Ecological, evolutionary & environmental sciences

For a reference copy of the document with all sections, see nature.com/documents/nr-reporting-summary-flat.pdf

Life sciences study design

All studies must disclose on these points even when the disclosure is negative.

Sample size	The SAP contains information on the prespecified numbers of evaluable COVID-19 endpoints for analyses to be conducted. Section 3.2 defines three time periods of follow-up and specifies that for each analysis for each time-period, if there a total of fewer than 25 evaluable COVID-19 endpoints available to include in the analysis, then the analysis will be canceled. However, some of the Super Learner analyses required a larger number of endpoints (at least 50) to be conducted, as shown in Table 2 of the SAP.<br>Further, subgroup analyses were only conducted when when the number of endpoints was equal to or exceeded 20, to ensure sufficient precision (Figures 4, 6, 7).
Data exclusions	The following sections of the SAP provide details on data exclusions:<br><br>SAP 2.1 Vaccine groups of interest for studying vaccine efficacy and correlates of protection:<br>•Pfizer-BioNTech vaccine arms 10 and 11 are excluded in comparison 1. because the Beta and Beta+Widtype/Prototype vaccines were not studied for the Moderna vaccine. In addition, Pfizer-BioNTech vaccine arms 16 and 17 are excluded in the mRNA Moderna vs. mRNA Pfizer-BioNTech booster comparison to retain a closer head-to-head comparison of vaccine-insert content.<br><br>SAP 2.2 Definitions of three time periods for capturing COVID-19 outcome cases included in correlates analyses::<br>•For D15 immune marker correlates analyses, follow-up for COVID-19 is right-censored at the date of receipt of the second dose (in all analyses), to avoid the complexity of a systematic perturbation in antibody response partway through follow-up for one study arm but not for others, which could complicate the interpretability of results. Descriptives show that zero COVID-19 endpoints are evaluable forrelates analyses for Arm 3 participants. Therefore Arm 3 participants contribute essentially no information about correlates. Accordingly, Arm 3 is excluded from the correlates analyses.<br><br>SAP 2.3 Figures and tables to describe the log10 ID50 markers for COVID-19 cases vs. non-cases<br>Tables of nAb ID50 titer results at each time point<br>•As noted, the sole 2-dose arm, Arm 3, is excluded from the tables given the different measurement schedule and the fact that right-censoring at the second dose implies inclusion of this arm would not provide significant information for the correlates analyses.<br><br>SAP 6.1 Baseline risk score development<br>•Study participants lost to follow-up without ever experiencing a COVID-19 endpoint are excluded from the analysis, for the relevant time-period of analysis.<br><br>SAP 9 Construction of Derived Variables for Data Analysis Based on the COVAIL Primary Data Set Received from Emmes 12-11-23<br>Eligibility for inclusion in immunogenicity and immune correlates analyses variables.<br>•The subset of participants with a COVID-19 event >= 7 days post D15 visit and up to 91 days post D15 visit are excluded from the analysis, such that ImmunemarkersetD92toD181 is the same as Immunemarkerset except this subset of participants has ImmunemarkersetD92toD181==0 & Immunemarkerset==1.

	COVID-19 endpoint failure time variables.<br>•Participants with a COVID-19 event >= 7 days post D15 visit and up to 91 days post D15 visit are excluded from the analysis.
Replication	For the neutralizing antibody assay, test samples were assayed in singlicate titrations per plate. Each plate included a SARS-CoV-2 positive control, a SARS-CoV-2 negative control, a daily positive control, and a specificity control.
Randomization	In th COVAIL trial, eligible participants were stratified by age (18-64 and >= 65 years) and history of confirmed SARS-CoV-2 infection, and randomly assigned across arms within each stage in an equal ratio. Randomization was stratified by previous infection status determined by self-report only, due to the delay in N-antibody testing.
Blinding	As reported in Branche et al. 2023 Nat Med, no blinding was performed as the immunogenicity endpoints did not require blinding to maintain rigor.

Reporting for specific materials, systems and methods

We require information from authors about some types of materials, experimental systems and methods used in many studies. Here, indicate whether each material, system or method listed is relevant to your study. If you are not sure if a list item applies to your research, read the appropriate section before selecting a response.

Materials & experimental systems

n/a	Involved in the study	
☒	☐	Antibodies
☐	☒	Eukaryotic cell lines
☒	☐	Palaeontology and archaeology
☒	☐	Animals and other organisms
☐	☒	Clinical data
☒	☐	Dual use research of concern
☒	☐	Plants

Methods

n/a	Involved in the study	
☒	☐	ChIP-seq
☒	☐	Flow cytometry
☒	☐	MRI-based neuroimaging

Eukaryotic cell lines

Policy information about cell lines and Sex and Gender in Research

Cell line source(s)	The pseudovirus neutralization assay used the HEK 293 cell line, sourced from the Master Cell Bank (LC0027490) established by Monogram Biosciences in 2001.
Authentication	No formal authentication was performed. The HEK293 cell line has been in continuous use at Monogram Biosciences since 1996.
Mycoplasma contamination	Mycoplasma testing is routinely performed per Monogram Standard Operating Procedure, and the HEK293 cell line is confirmed to test negative for mycoplasma contamination.
Commonly misidentified lines (See ICLAC register)	None.

Clinical data

Policy information about clinical studies

All manuscripts should comply with the ICMJE guidelines for publication of clinical research and a completed CONSORT checklist must be included with all submissions.

Clinical trial registration	ClinicalTrials.gov ID NCT05289037
Study protocol	The full study protocol for the COVAIL trial is provided in the supplemental methods of Branche et al. 2023 Nature Medicine.
Data collection	Participants were recruited at 22 US sites comprised of research clinics associated with academic medical institutions. These sites are listed in the Supplementary Material. All clinical data was collected during scheduled visits. Recruitment for Stages 1-3 occurred from March 2022-June 2022. Data collection was completed for these stages in August 2023. Recruitment for Stage 4 occurred October 2022 and data collection was completed in November 2023.
Outcomes	Branche et al. 2023 Nat Med and Branche et al. 2023 CID describe the primary and secondary outcomes of the COVAIL trial.<br>In the present analysis, the COVID-19 endpoint was a self-reported positive SARS-CoV-2 test (RT-PCR or antigen test) or study-conducted positive SARS-CoV-2 test (nasal swab and subsequent nucleic acid amplification test at an unscheduled illness visit) with onset date the earliest positive test date. Almost all (208/213) COVID-19 endpoints met the CDC clinical criteria and supportive laboratory criteria for a COVID-19 surveillance case (Supplementary Table 4).

Plants

Seed stocks	N/A
Novel plant genotypes	N/A
Authentication	N/A
